# Supplementary material for: JHY enables the transition from switchable to fixed ciliary waveforms in metazoan evolution
Source: EMBO Rep. 2025 Dec 10;27(5):1161–79. doi: 10.1038/s44319-025-00671-7 (PMC12979858; doi:10.1038/s44319-025-00671-7)
Supplement: Supplementary file 8 — Movie EV1 [file 44319_2025_671_MOESM8_ESM.zip › Movie EV1/Movie legend.docx]

**Movie legend**

**Movie EV1. Ciliary motilities in representative regions of WT and *Jhy* KO mEPCs.**

Motilities of multicilia in WT and *Jhy* KO mEPCs were stained with SiR-tubulin and live imaged. Image sequences are played back at 5 fps.
