# Supplementary figures and images for: JHY enables the transition from switchable to fixed ciliary waveforms in metazoan evolution
Source: EMBO Rep. 2025 Dec 10;27(5):1161–79. doi: 10.1038/s44319-025-00671-7 (PMC12979858; doi:10.1038/s44319-025-00671-7)

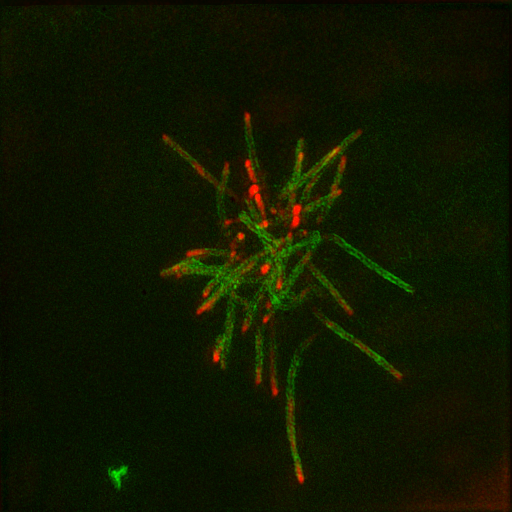

Supplement: Supplementary file 9 — Source data Fig. 1 [file 44319_2025_671_MOESM9_ESM.zip › SD figure 1/1B APEX-WDR47 Biotin-Phenol.tif]

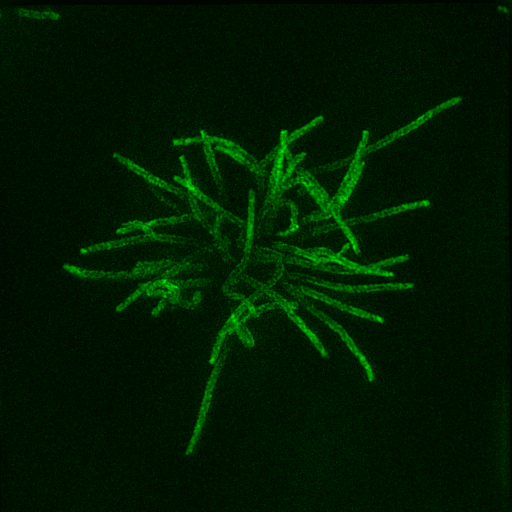

Supplement: Supplementary file 9 — Source data Fig. 1 [file 44319_2025_671_MOESM9_ESM.zip › SD figure 1/1B APEX-WDR47 DMSO.tif]

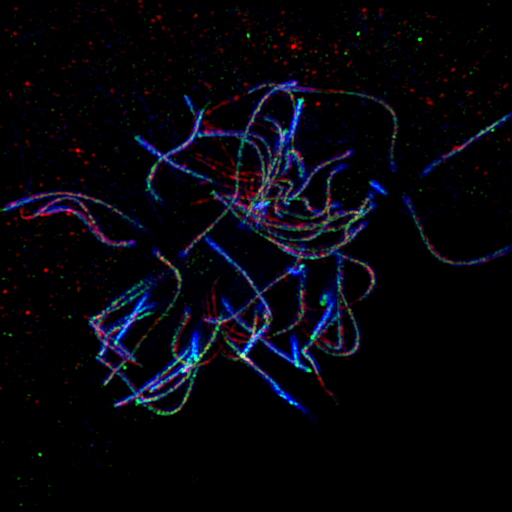

Supplement: Supplementary file 10 — Source data Fig. 2 [file 44319_2025_671_MOESM10_ESM.zip › SD figure 2/2B GFP-JHY.tif]

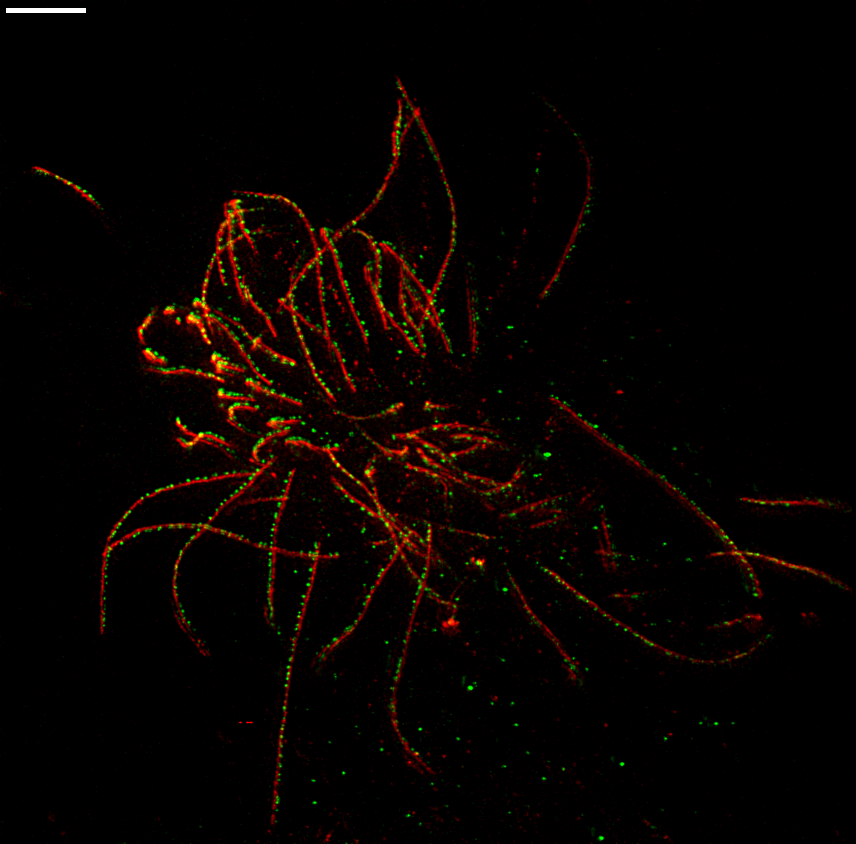

Supplement: Supplementary file 10 — Source data Fig. 2 [file 44319_2025_671_MOESM10_ESM.zip › SD figure 2/2D GFP-ENO4 Kif9.tif]

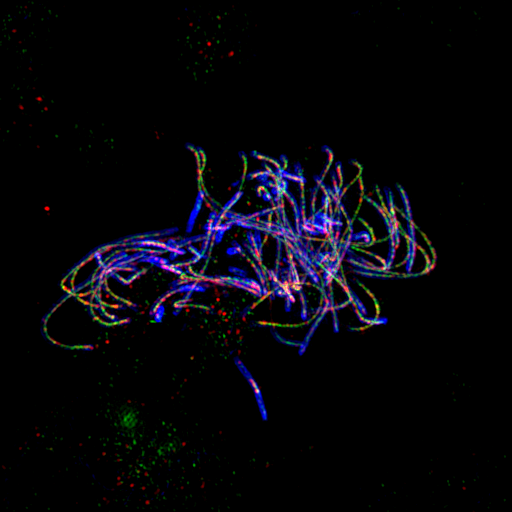

Supplement: Supplementary file 10 — Source data Fig. 2 [file 44319_2025_671_MOESM10_ESM.zip › SD figure 2/2A GFP-MYCBPAP.tif]

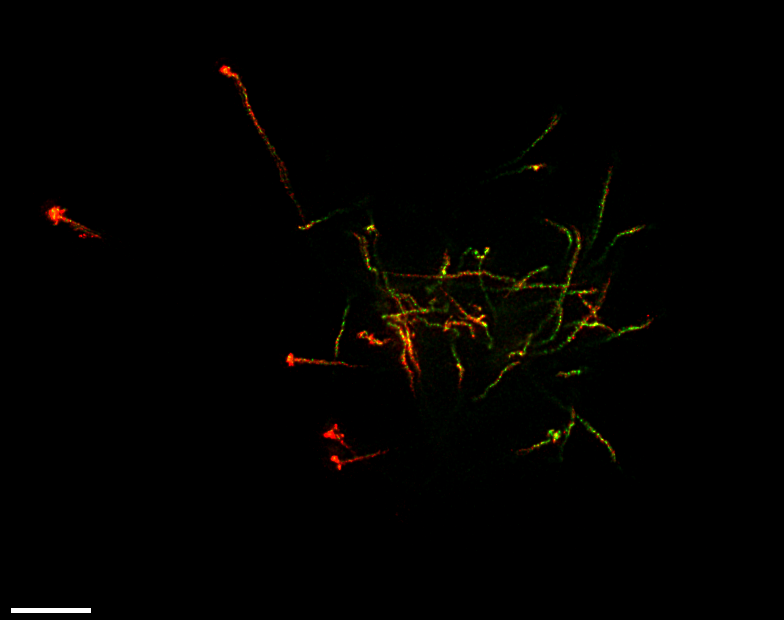

Supplement: Supplementary file 10 — Source data Fig. 2 [file 44319_2025_671_MOESM10_ESM.zip › SD figure 2/2D GFP-JHY Kif9.tif]

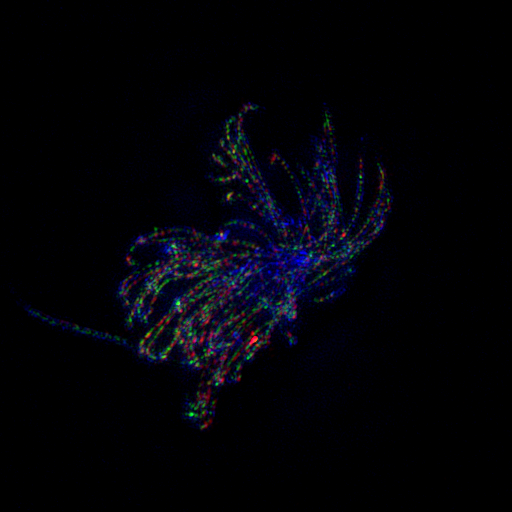

Supplement: Supplementary file 10 — Source data Fig. 2 [file 44319_2025_671_MOESM10_ESM.zip › SD figure 2/2A GFP-LRGUK.tif]

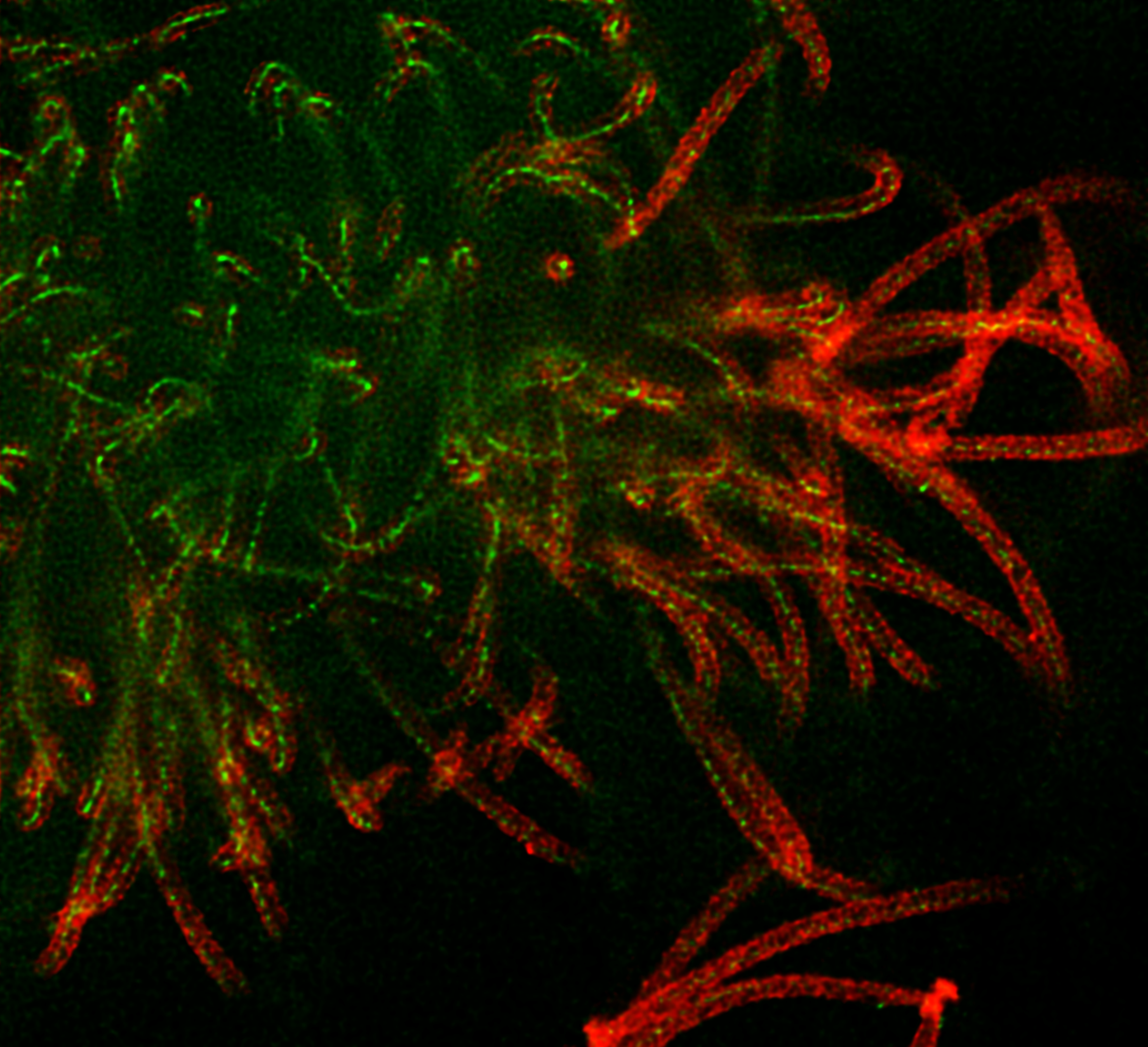

Supplement: Supplementary file 10 — Source data Fig. 2 [file 44319_2025_671_MOESM10_ESM.zip › SD figure 2/2C GFP-ENO4.tif]

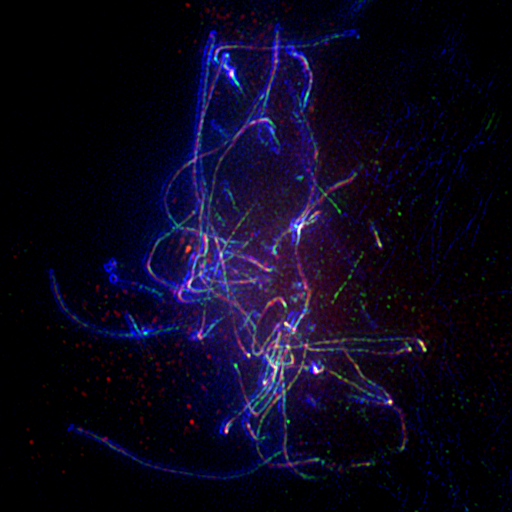

Supplement: Supplementary file 10 — Source data Fig. 2 [file 44319_2025_671_MOESM10_ESM.zip › SD figure 2/2B GFP-C2ORF81.tif]

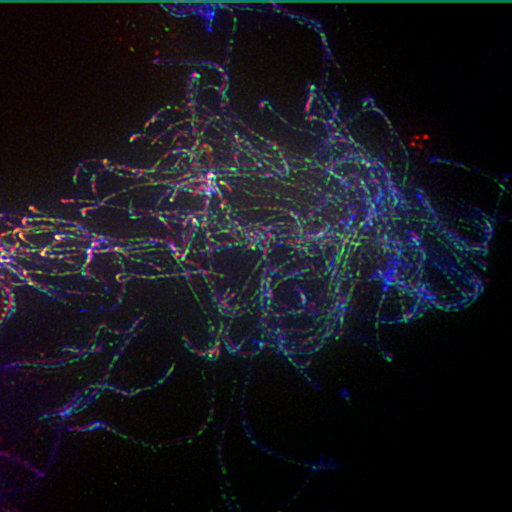

Supplement: Supplementary file 10 — Source data Fig. 2 [file 44319_2025_671_MOESM10_ESM.zip › SD figure 2/2A GFP-ENO4.tif]

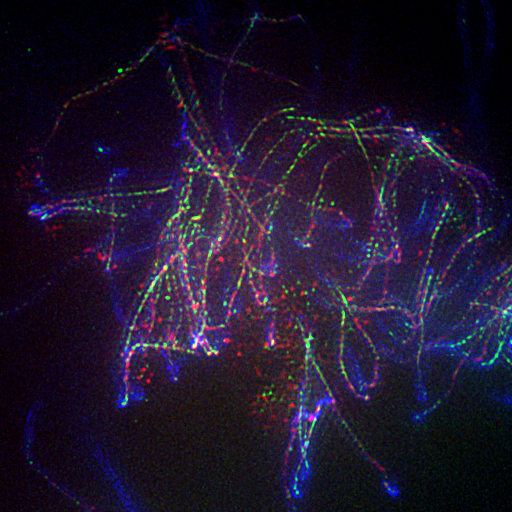

Supplement: Supplementary file 10 — Source data Fig. 2 [file 44319_2025_671_MOESM10_ESM.zip › SD figure 2/2B GFP-LRRC71.tif]

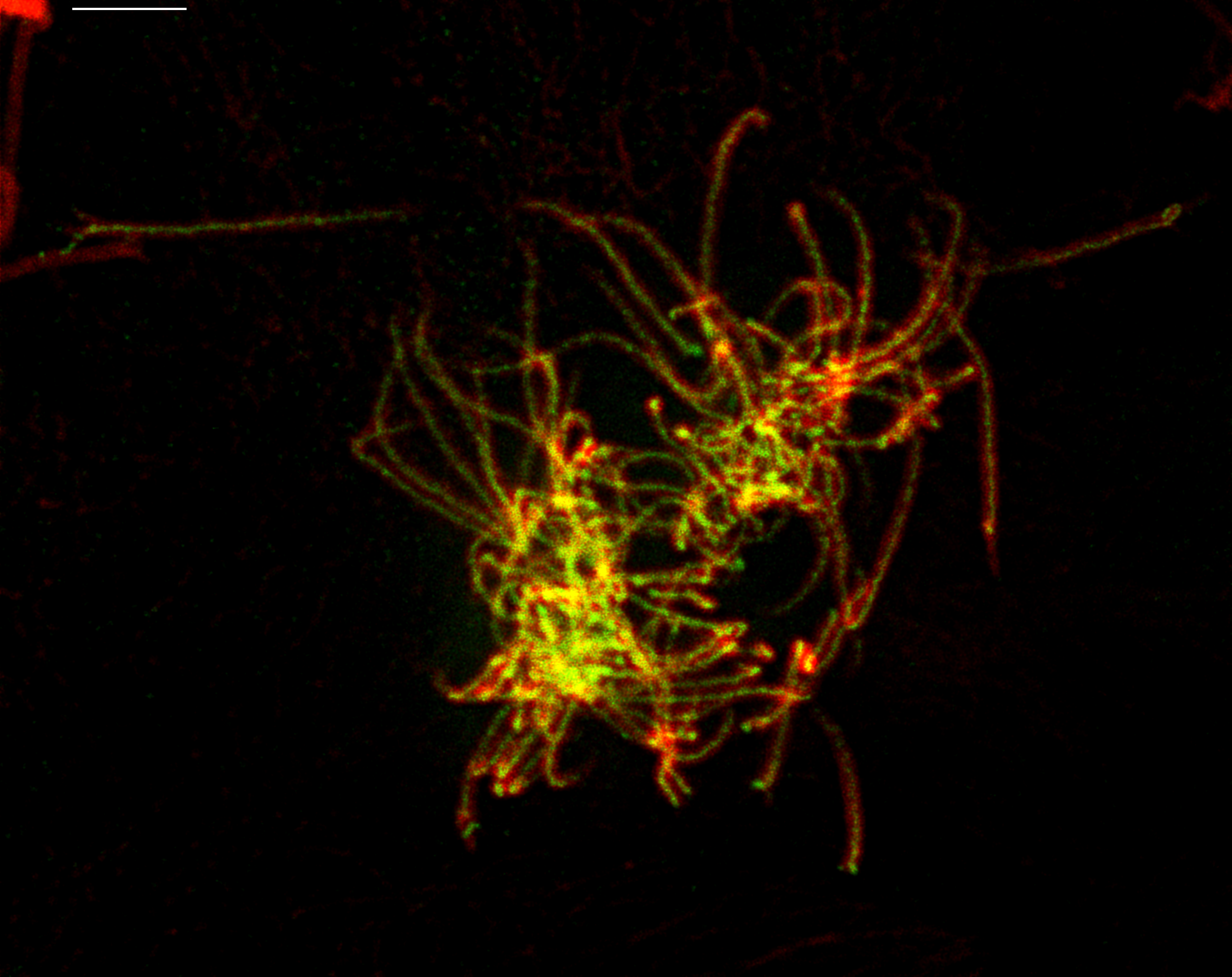

Supplement: Supplementary file 10 — Source data Fig. 2 [file 44319_2025_671_MOESM10_ESM.zip › SD figure 2/2C GFP-JHY.tif]

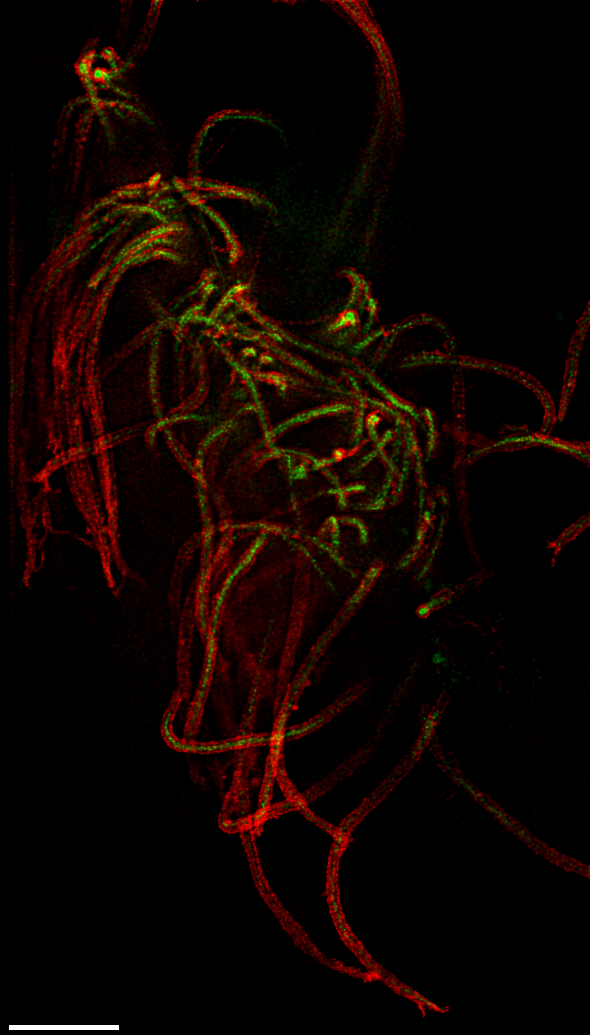

Supplement: Supplementary file 10 — Source data Fig. 2 [file 44319_2025_671_MOESM10_ESM.zip › SD figure 2/2C GFP-CCDC13.tif]

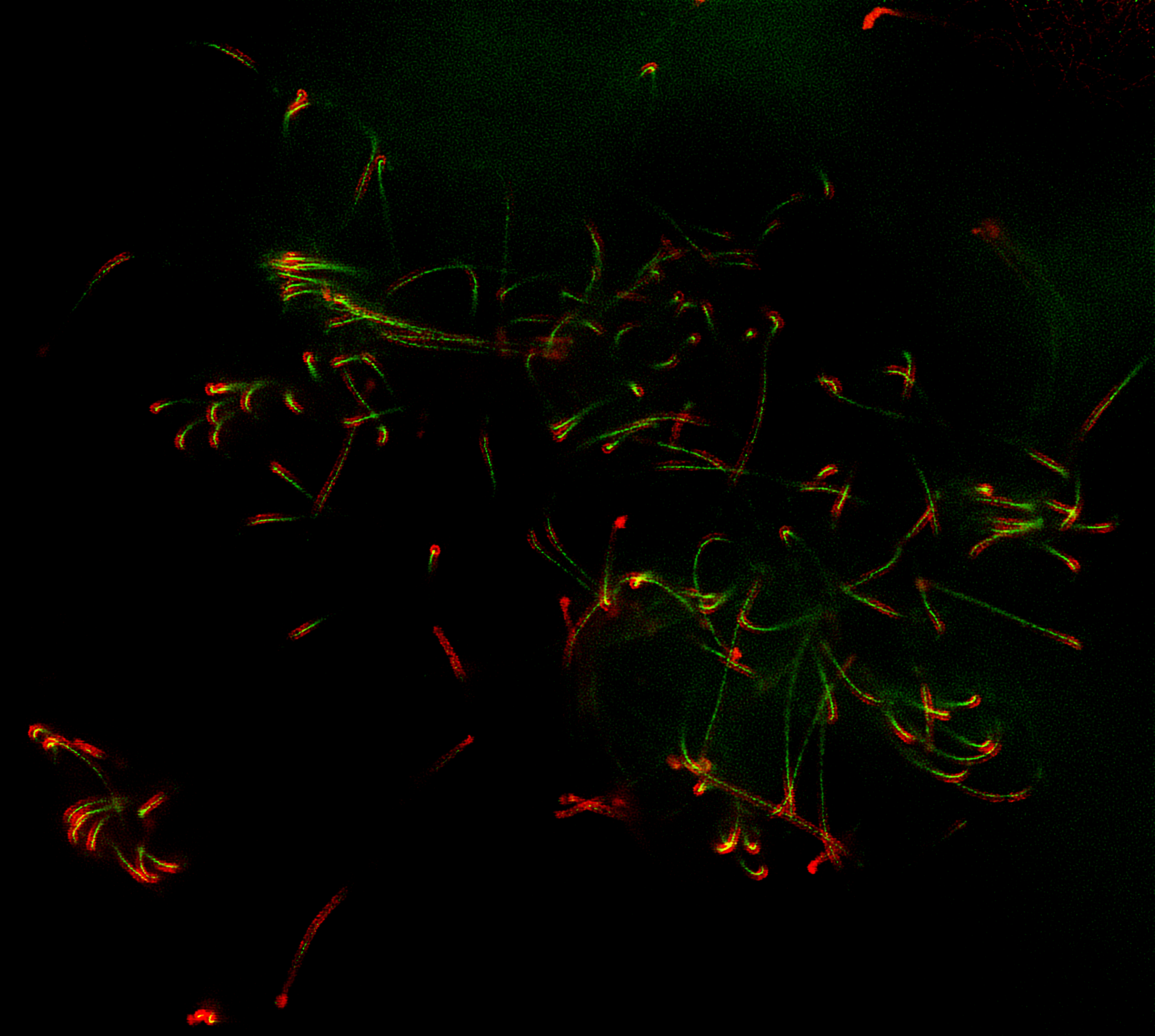

Supplement: Supplementary file 10 — Source data Fig. 2 [file 44319_2025_671_MOESM10_ESM.zip › SD figure 2/2C GFP-C2orf81.tif]

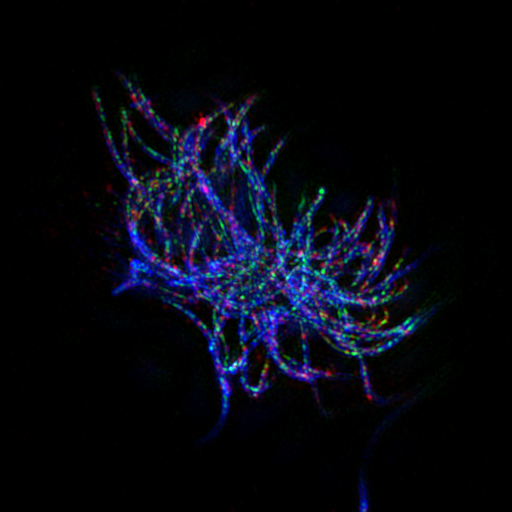

Supplement: Supplementary file 10 — Source data Fig. 2 [file 44319_2025_671_MOESM10_ESM.zip › SD figure 2/2B GFP-CCDC13.tif]

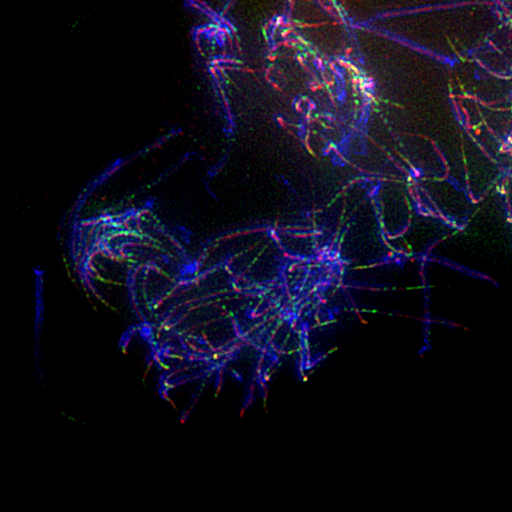

Supplement: Supplementary file 10 — Source data Fig. 2 [file 44319_2025_671_MOESM10_ESM.zip › SD figure 2/2A GFP-CCDC108.tif]

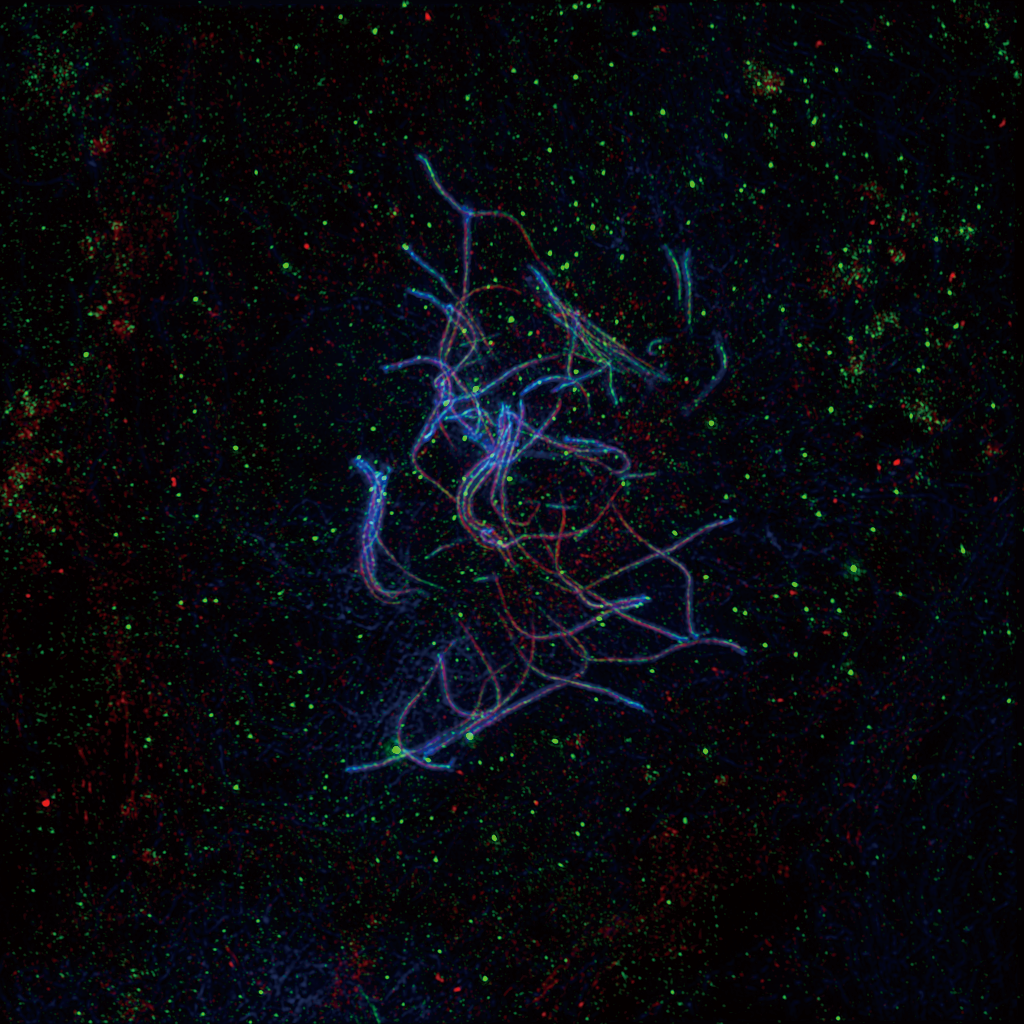

Supplement: Supplementary file 11 — Source data Fig. 3 [file 44319_2025_671_MOESM11_ESM.zip › SD figure 3/3A JHY HYDIN.tif]

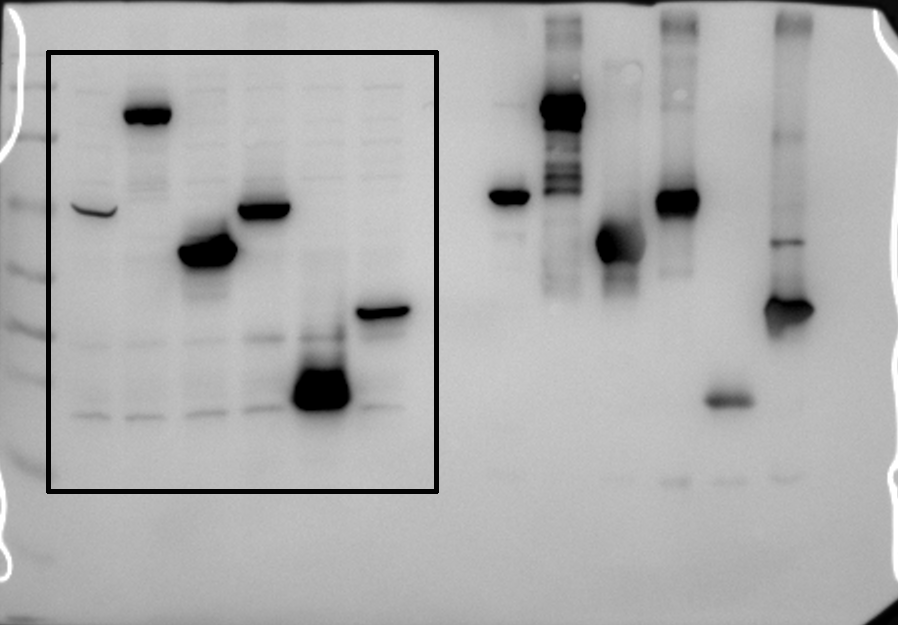

Supplement: Supplementary file 11 — Source data Fig. 3 [file 44319_2025_671_MOESM11_ESM.zip › SD figure 3/3F FLAG input.tif]

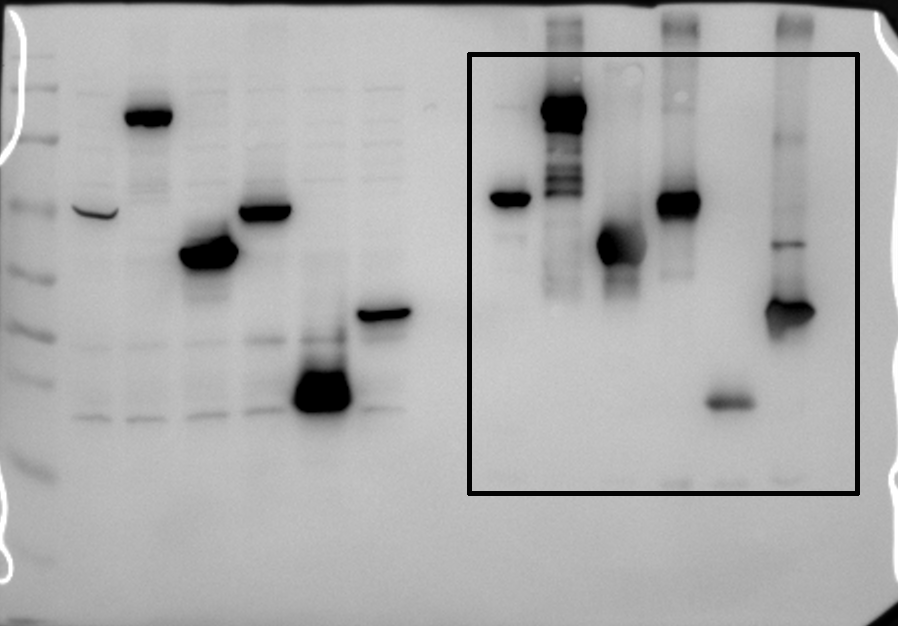

Supplement: Supplementary file 11 — Source data Fig. 3 [file 44319_2025_671_MOESM11_ESM.zip › SD figure 3/3F FLAG IP.tif]

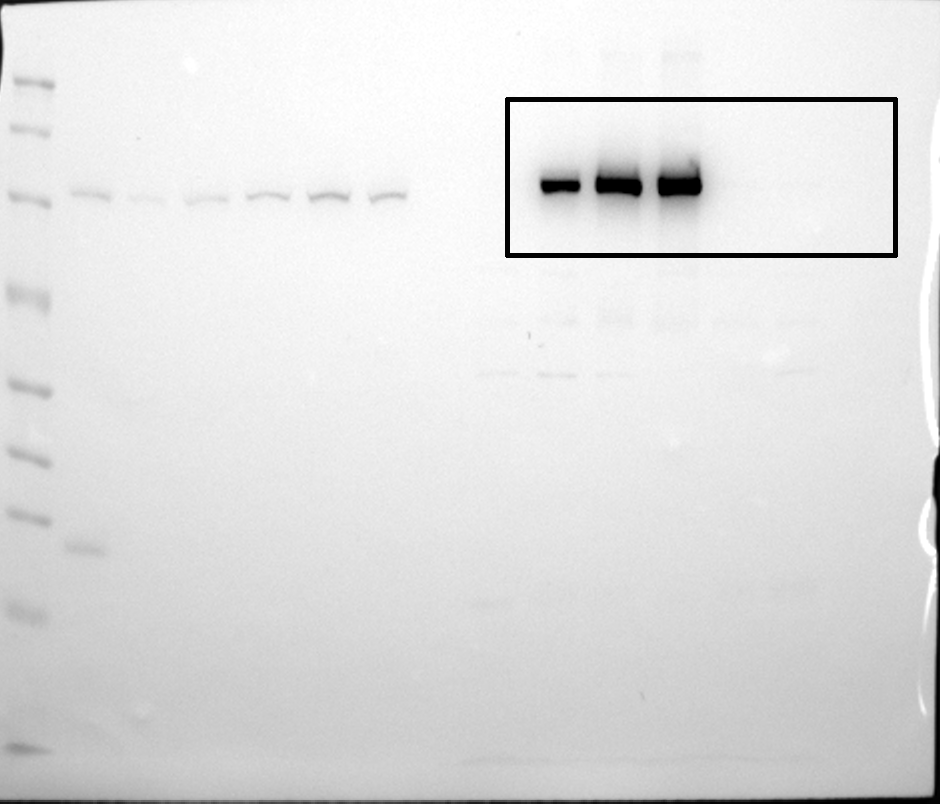

Supplement: Supplementary file 11 — Source data Fig. 3 [file 44319_2025_671_MOESM11_ESM.zip › SD figure 3/3E WDR47 IP.tif]

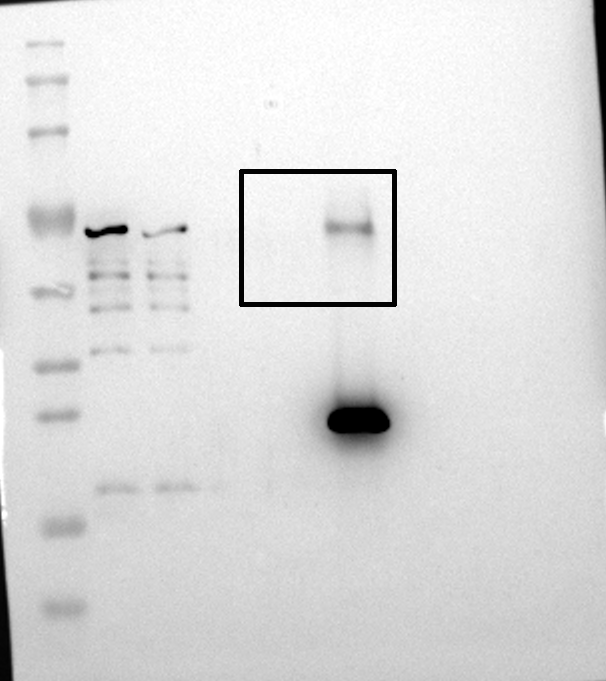

Supplement: Supplementary file 11 — Source data Fig. 3 [file 44319_2025_671_MOESM11_ESM.zip › SD figure 3/3H His GST-trap.tif]

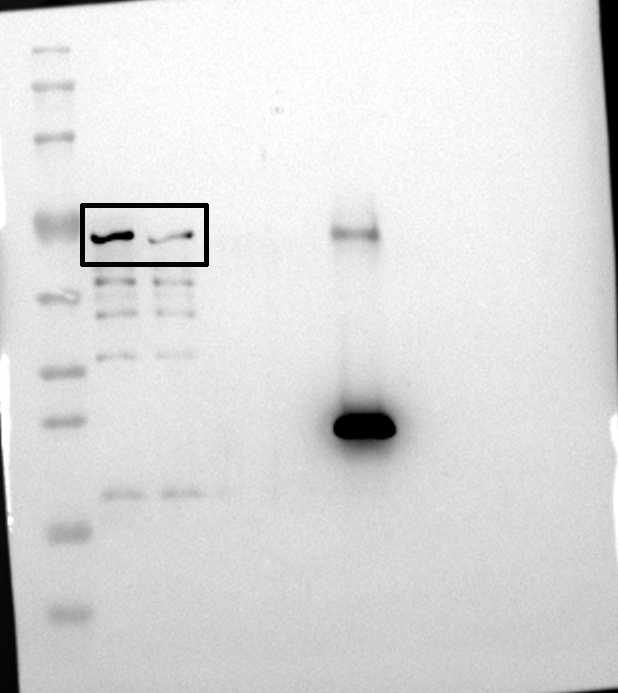

Supplement: Supplementary file 11 — Source data Fig. 3 [file 44319_2025_671_MOESM11_ESM.zip › SD figure 3/3H His input.tif]

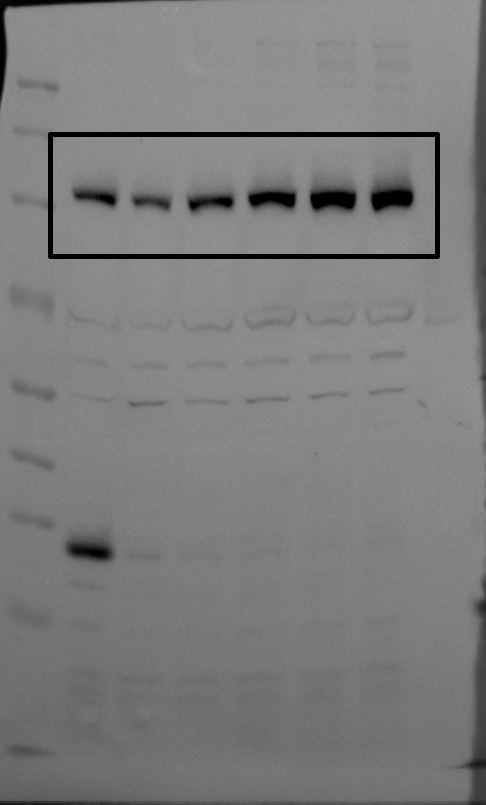

Supplement: Supplementary file 11 — Source data Fig. 3 [file 44319_2025_671_MOESM11_ESM.zip › SD figure 3/3E WDR47 input.tif]

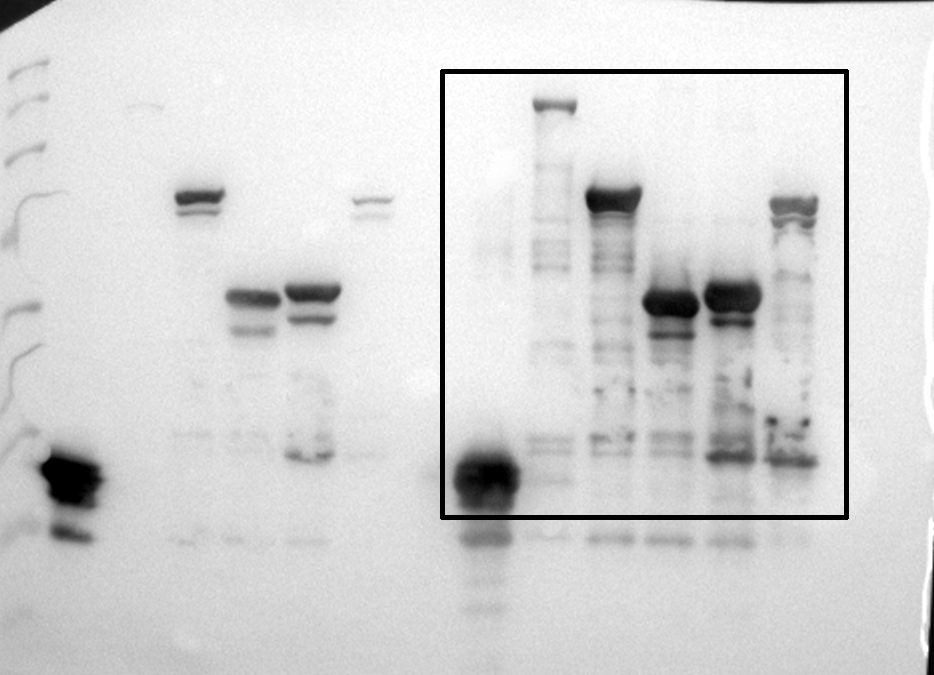

Supplement: Supplementary file 11 — Source data Fig. 3 [file 44319_2025_671_MOESM11_ESM.zip › SD figure 3/3E GFP IP.tif]

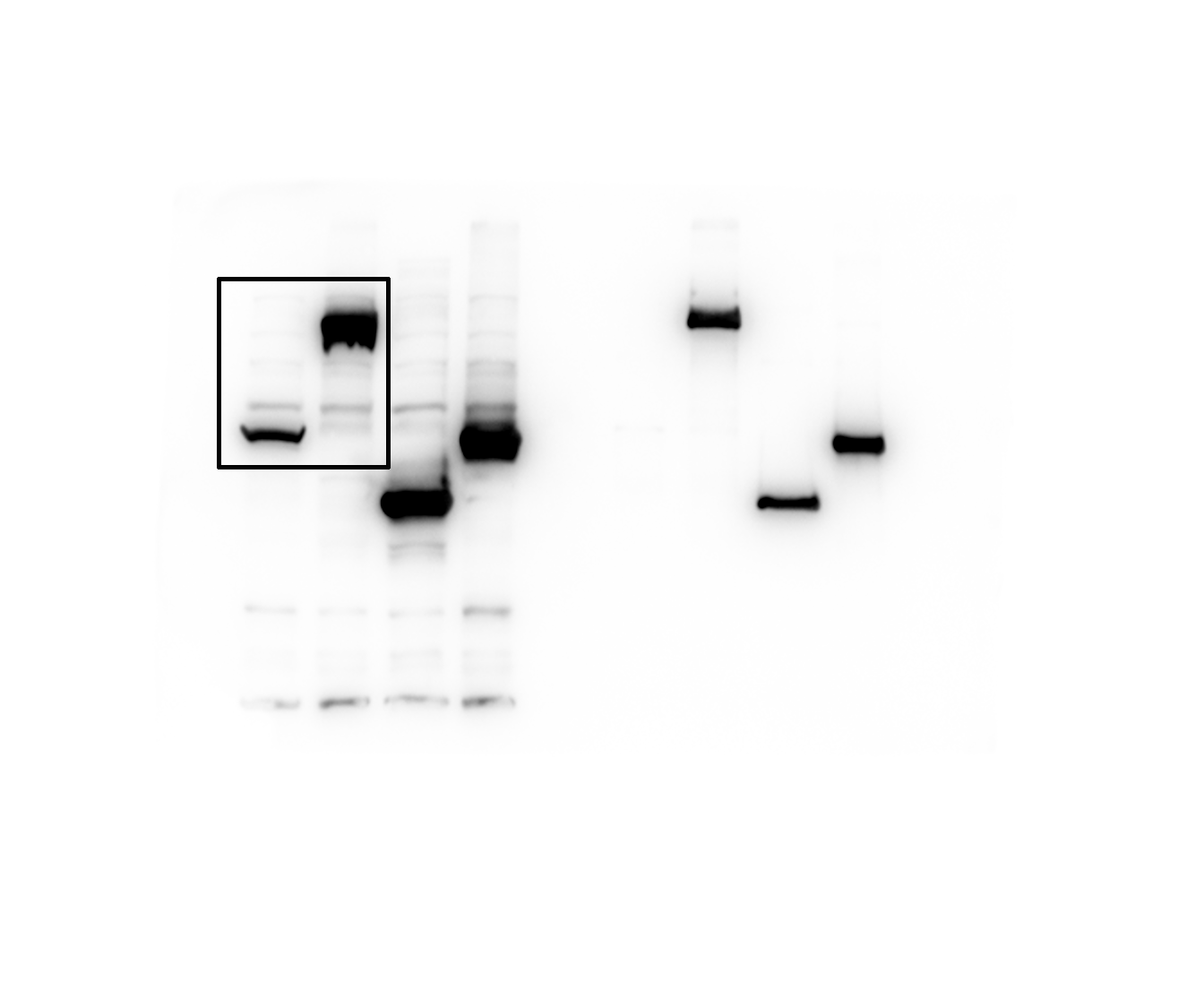

Supplement: Supplementary file 11 — Source data Fig. 3 [file 44319_2025_671_MOESM11_ESM.zip › SD figure 3/3D FLAG input.tif]

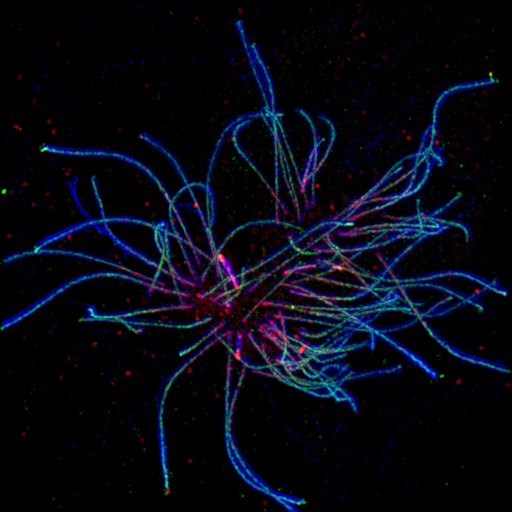

Supplement: Supplementary file 11 — Source data Fig. 3 [file 44319_2025_671_MOESM11_ESM.zip › SD figure 3/3B GFP-JHY WDR47 bottom.tif]

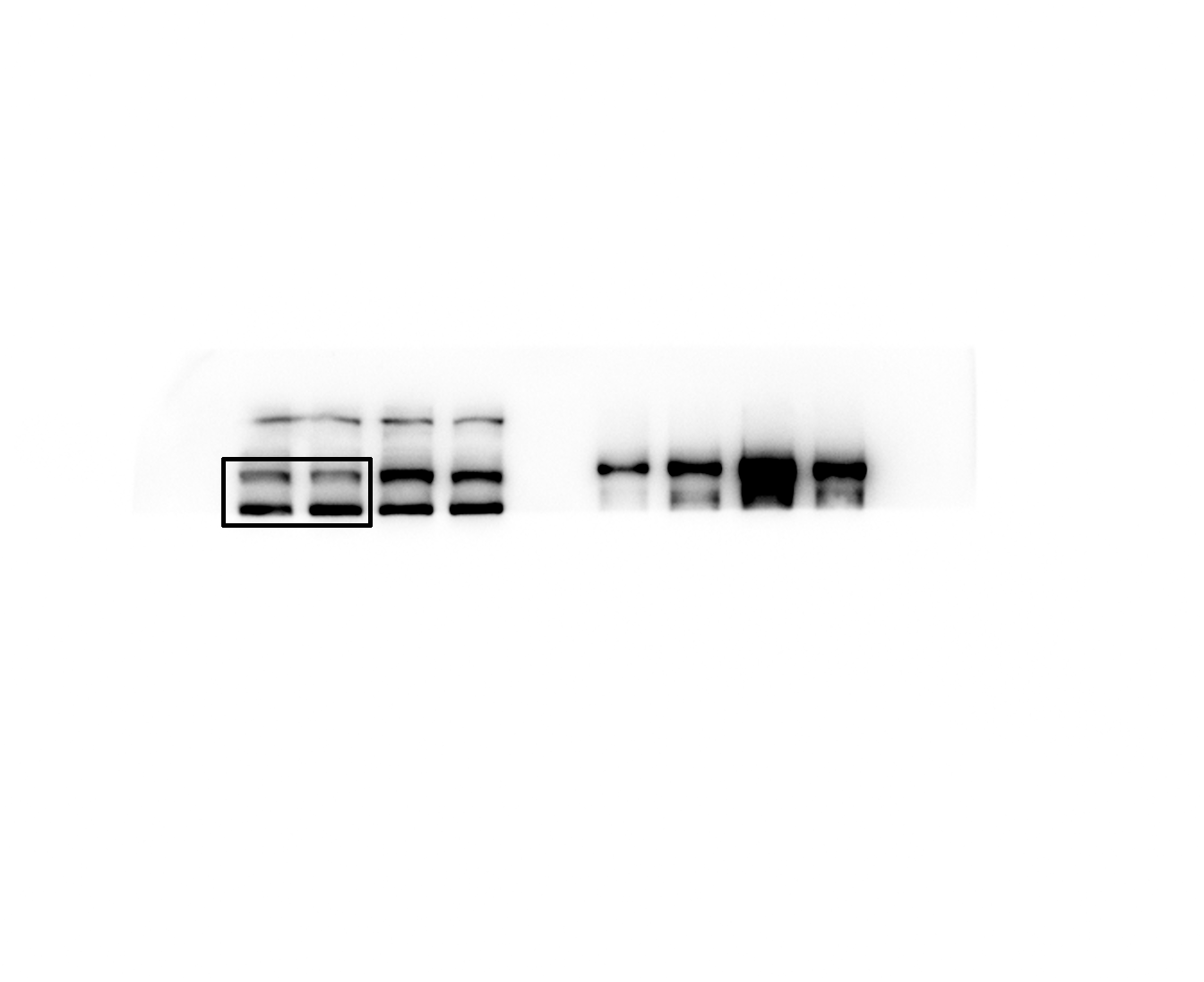

Supplement: Supplementary file 11 — Source data Fig. 3 [file 44319_2025_671_MOESM11_ESM.zip › SD figure 3/3D GFP input.tif]

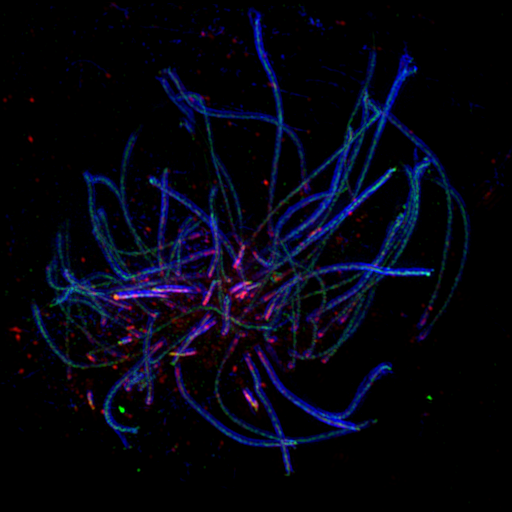

Supplement: Supplementary file 11 — Source data Fig. 3 [file 44319_2025_671_MOESM11_ESM.zip › SD figure 3/3B GFP-JHY WDR47 upper.tif]

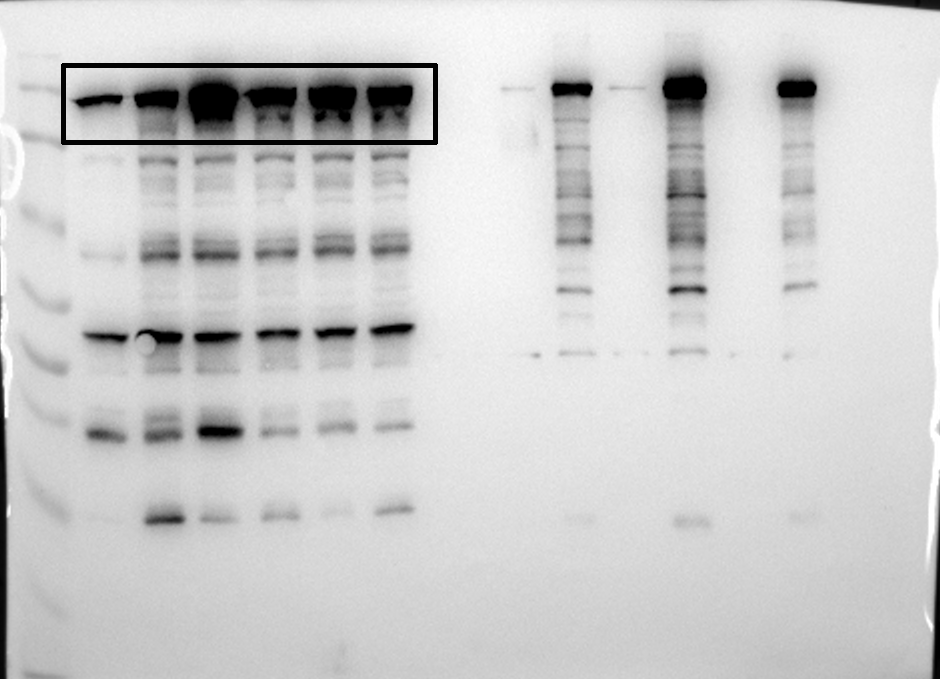

Supplement: Supplementary file 11 — Source data Fig. 3 [file 44319_2025_671_MOESM11_ESM.zip › SD figure 3/3F GFP input.tif]

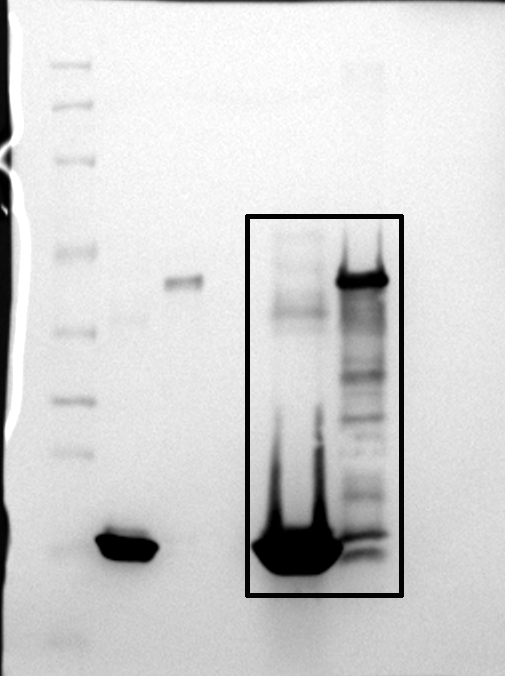

Supplement: Supplementary file 11 — Source data Fig. 3 [file 44319_2025_671_MOESM11_ESM.zip › SD figure 3/3H GST GST-trap.tif]

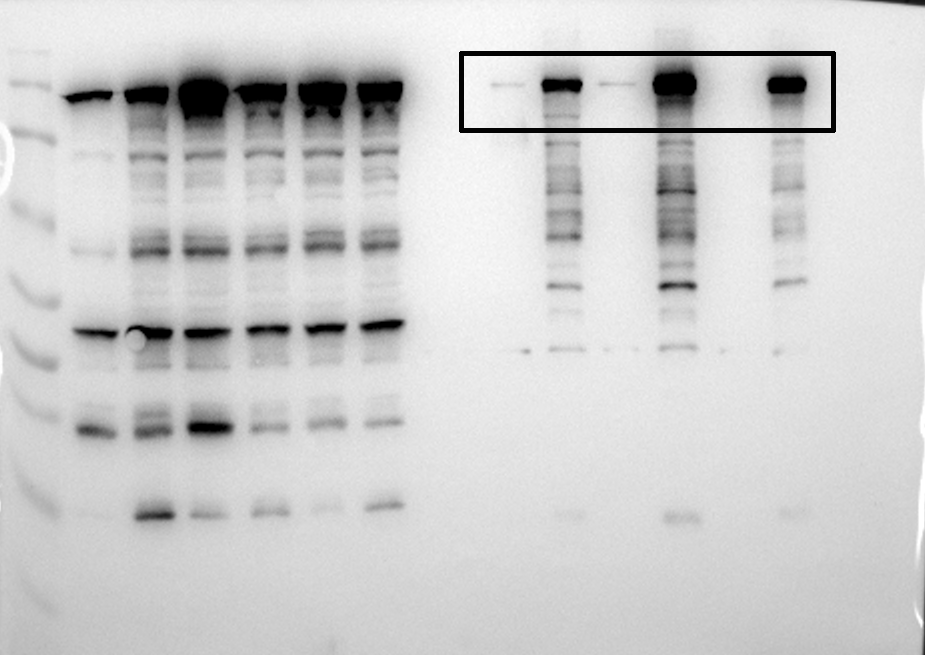

Supplement: Supplementary file 11 — Source data Fig. 3 [file 44319_2025_671_MOESM11_ESM.zip › SD figure 3/3F GFP IP.tif]

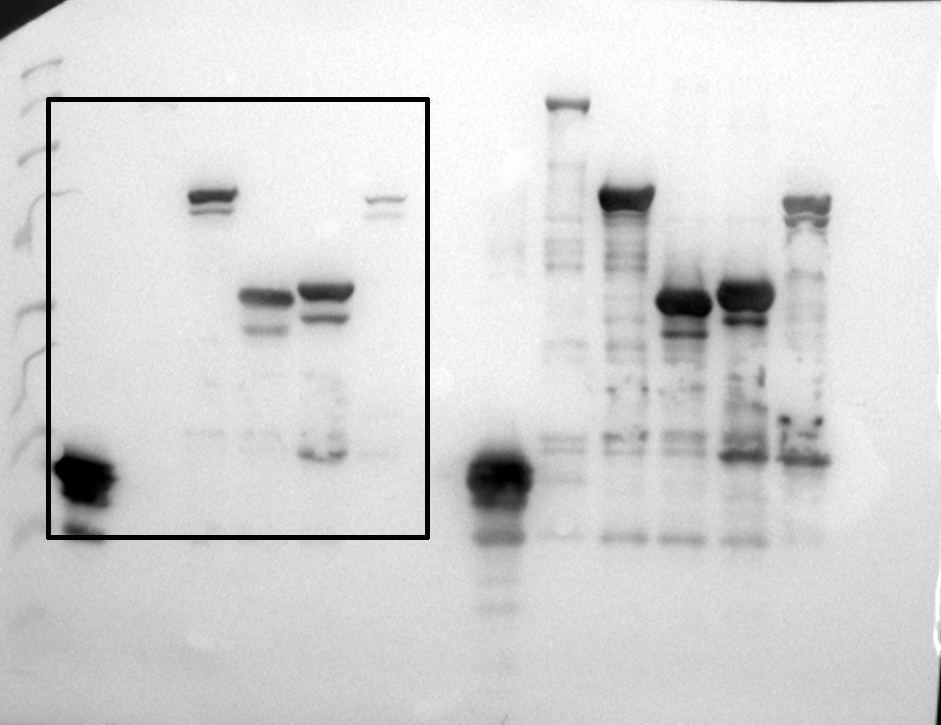

Supplement: Supplementary file 11 — Source data Fig. 3 [file 44319_2025_671_MOESM11_ESM.zip › SD figure 3/3E GFP input.tif]

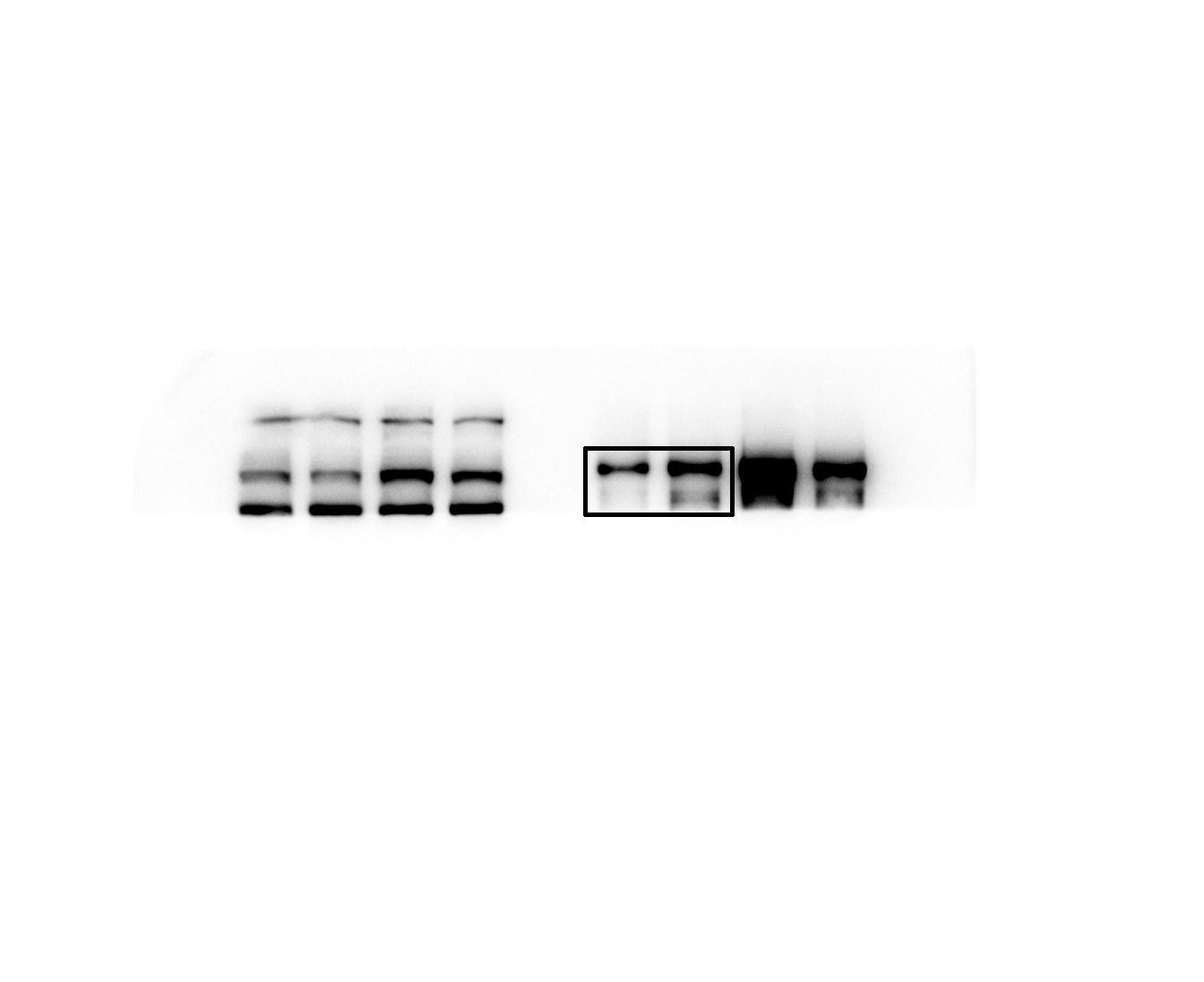

Supplement: Supplementary file 11 — Source data Fig. 3 [file 44319_2025_671_MOESM11_ESM.zip › SD figure 3/3D GFP IP.tif]

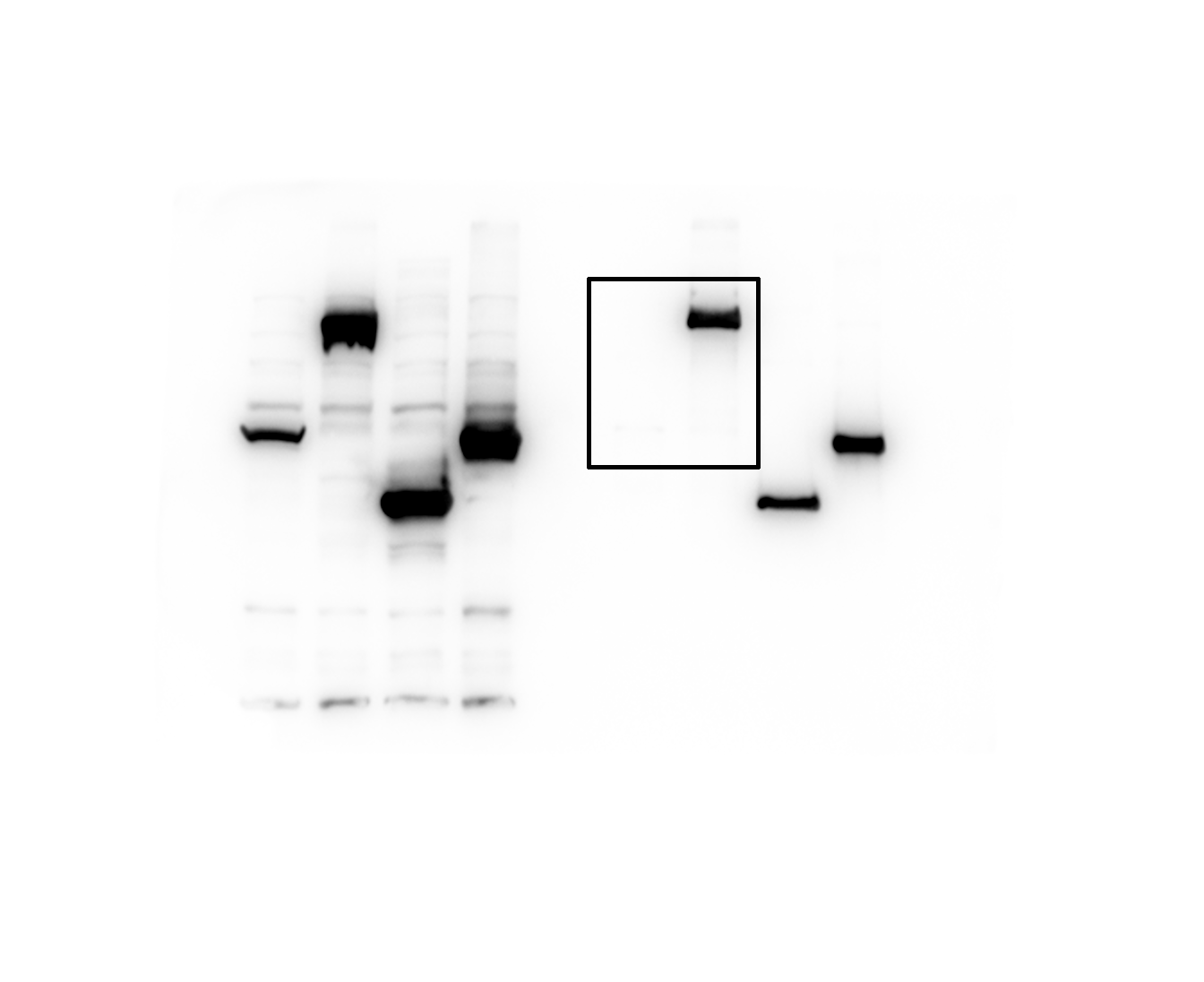

Supplement: Supplementary file 11 — Source data Fig. 3 [file 44319_2025_671_MOESM11_ESM.zip › SD figure 3/3D FLAG IP.tif]

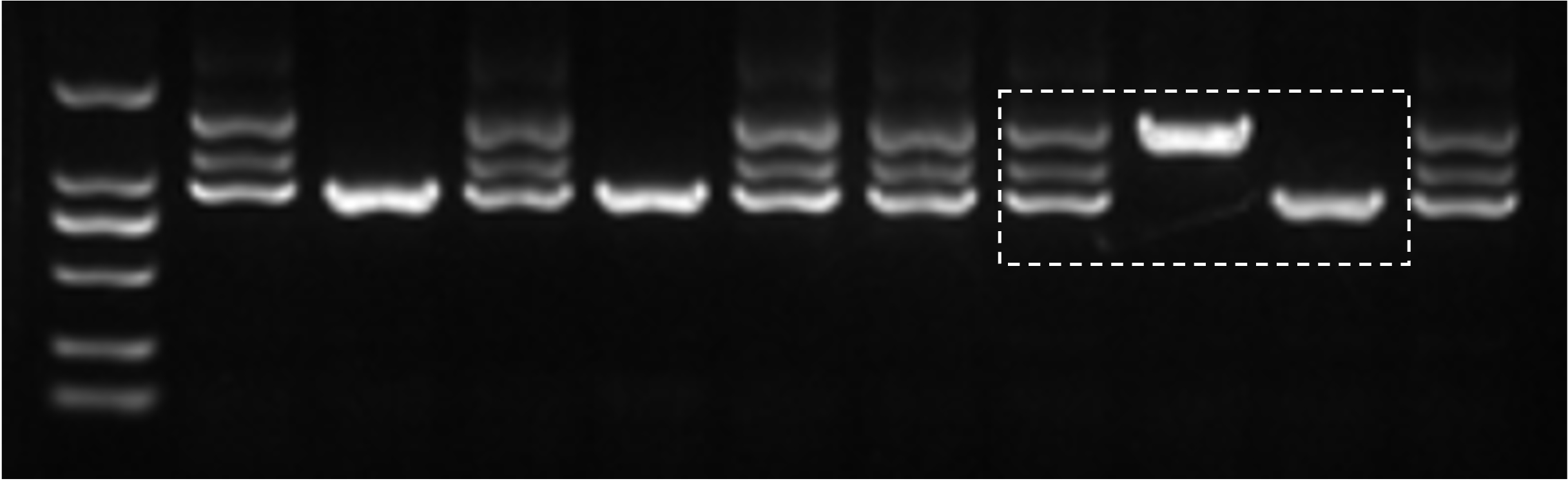

Supplement: Supplementary file 12 — Source data Fig. 4 [file 44319_2025_671_MOESM12_ESM.zip › SD figure 4/4C F1R1.jpg]

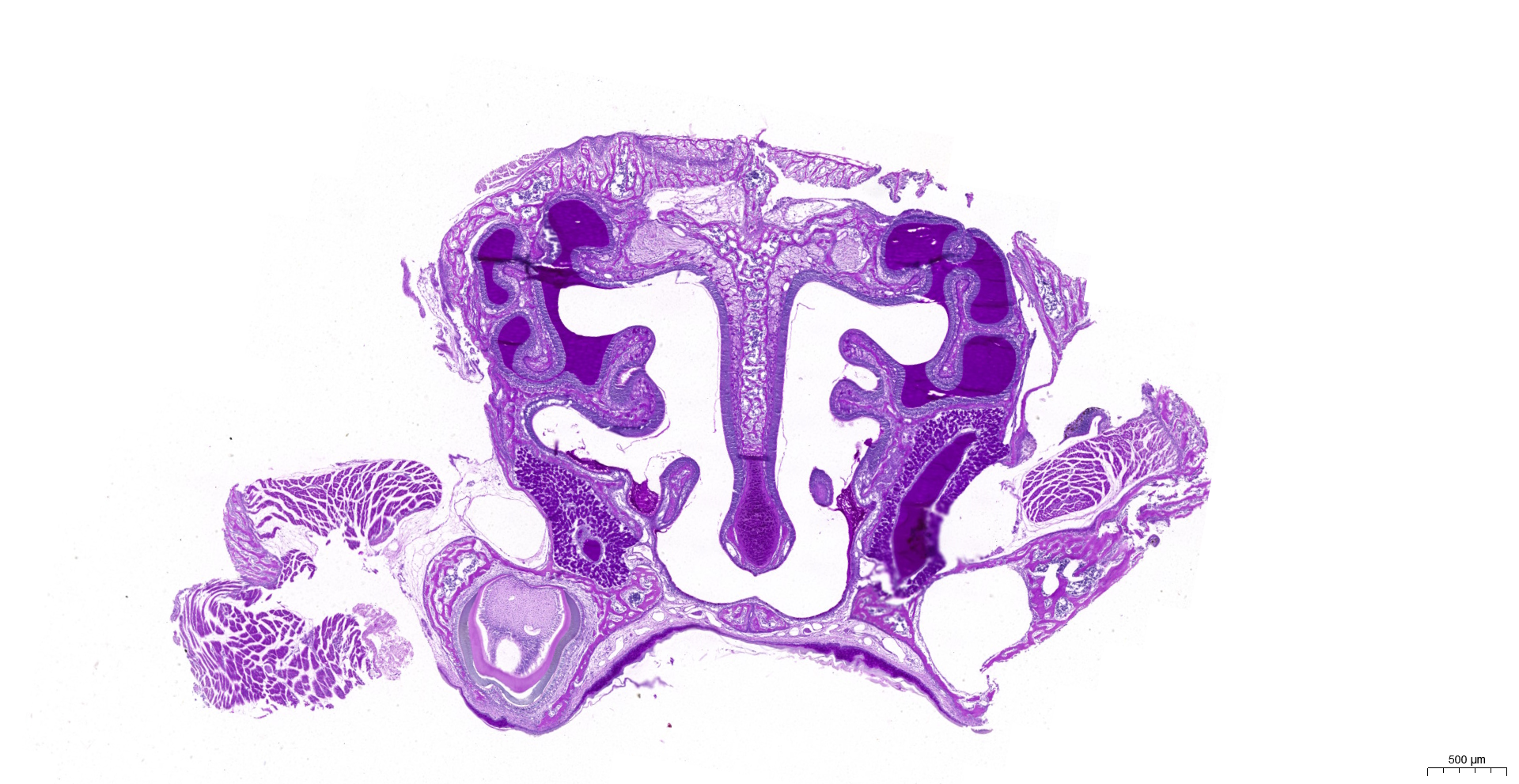

Supplement: Supplementary file 12 — Source data Fig. 4 [file 44319_2025_671_MOESM12_ESM.zip › SD figure 4/4H JHY-ko-40_2.tif]

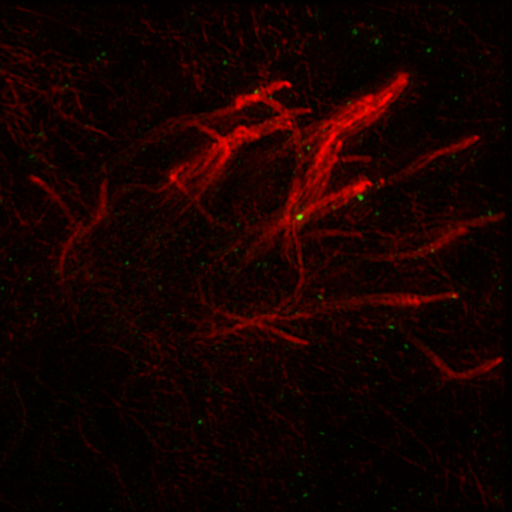

Supplement: Supplementary file 12 — Source data Fig. 4 [file 44319_2025_671_MOESM12_ESM.zip › SD figure 4/4D KO JHY.tif]

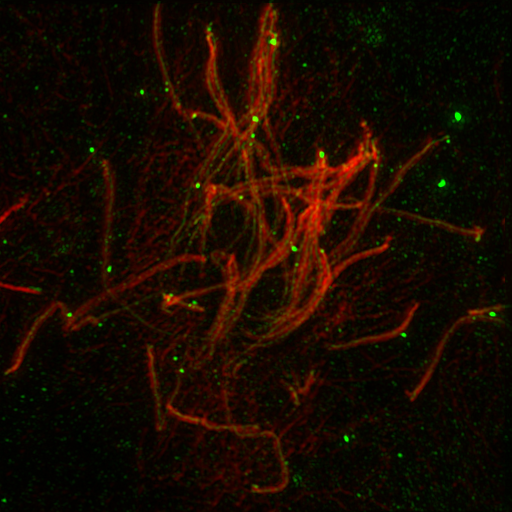

Supplement: Supplementary file 12 — Source data Fig. 4 [file 44319_2025_671_MOESM12_ESM.zip › SD figure 4/4D WT JHY.tif]

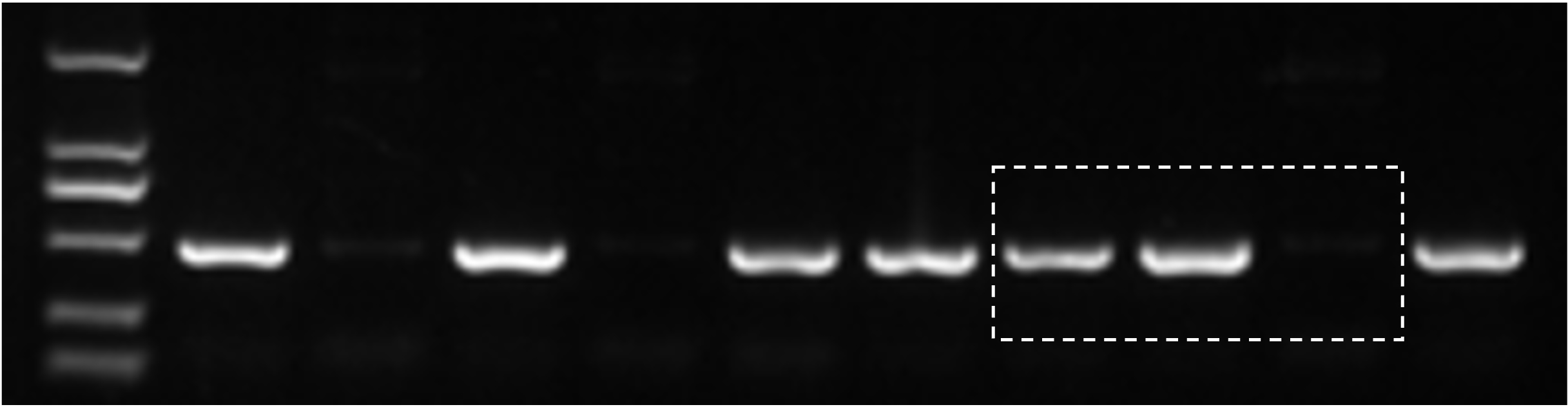

Supplement: Supplementary file 12 — Source data Fig. 4 [file 44319_2025_671_MOESM12_ESM.zip › SD figure 4/4C F2R2.jpg]

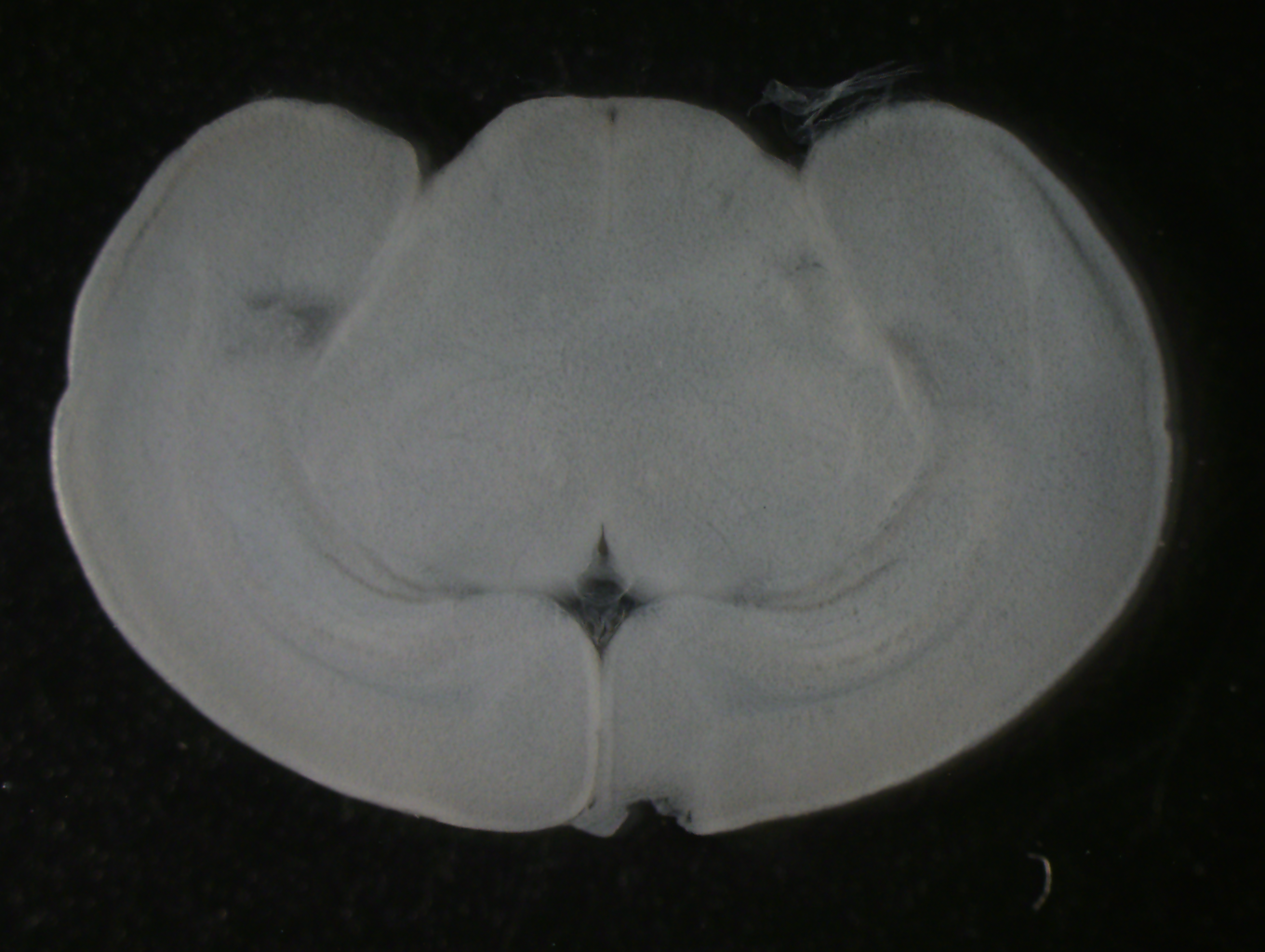

Supplement: Supplementary file 12 — Source data Fig. 4 [file 44319_2025_671_MOESM12_ESM.zip › SD figure 4/4G WT 4.tif]

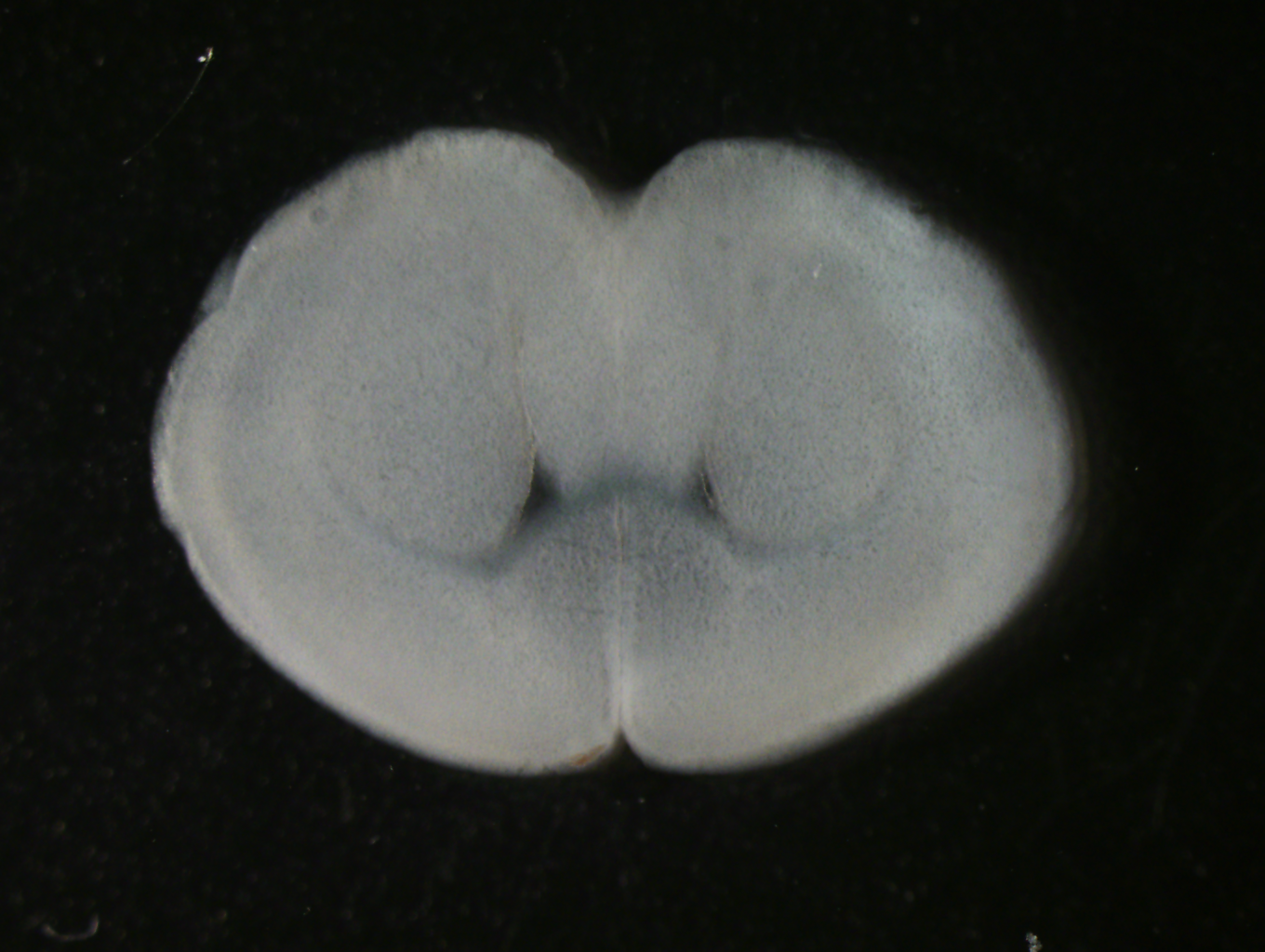

Supplement: Supplementary file 12 — Source data Fig. 4 [file 44319_2025_671_MOESM12_ESM.zip › SD figure 4/4G WT 1.tif]

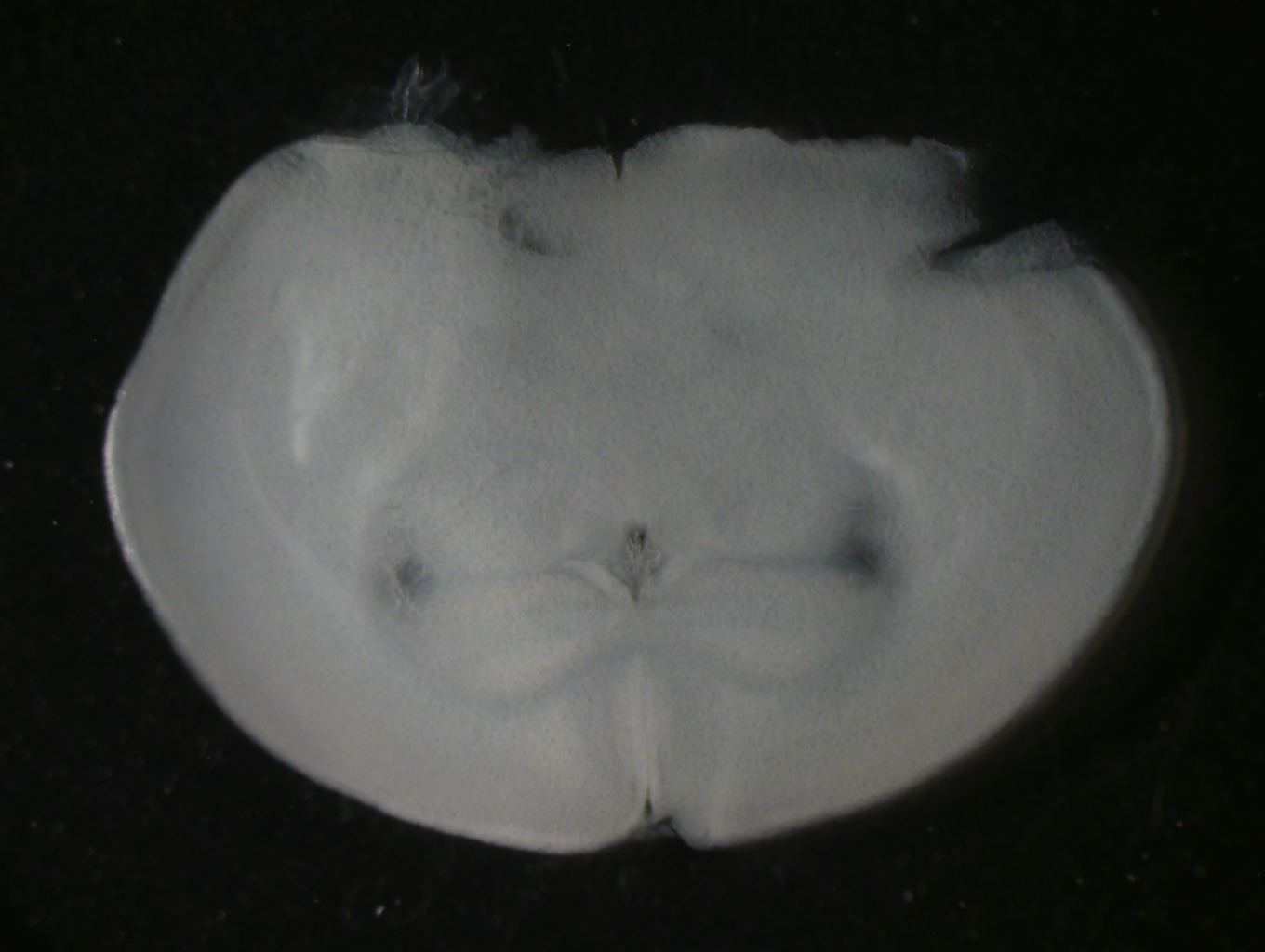

Supplement: Supplementary file 12 — Source data Fig. 4 [file 44319_2025_671_MOESM12_ESM.zip › SD figure 4/4G WT 3.tif]

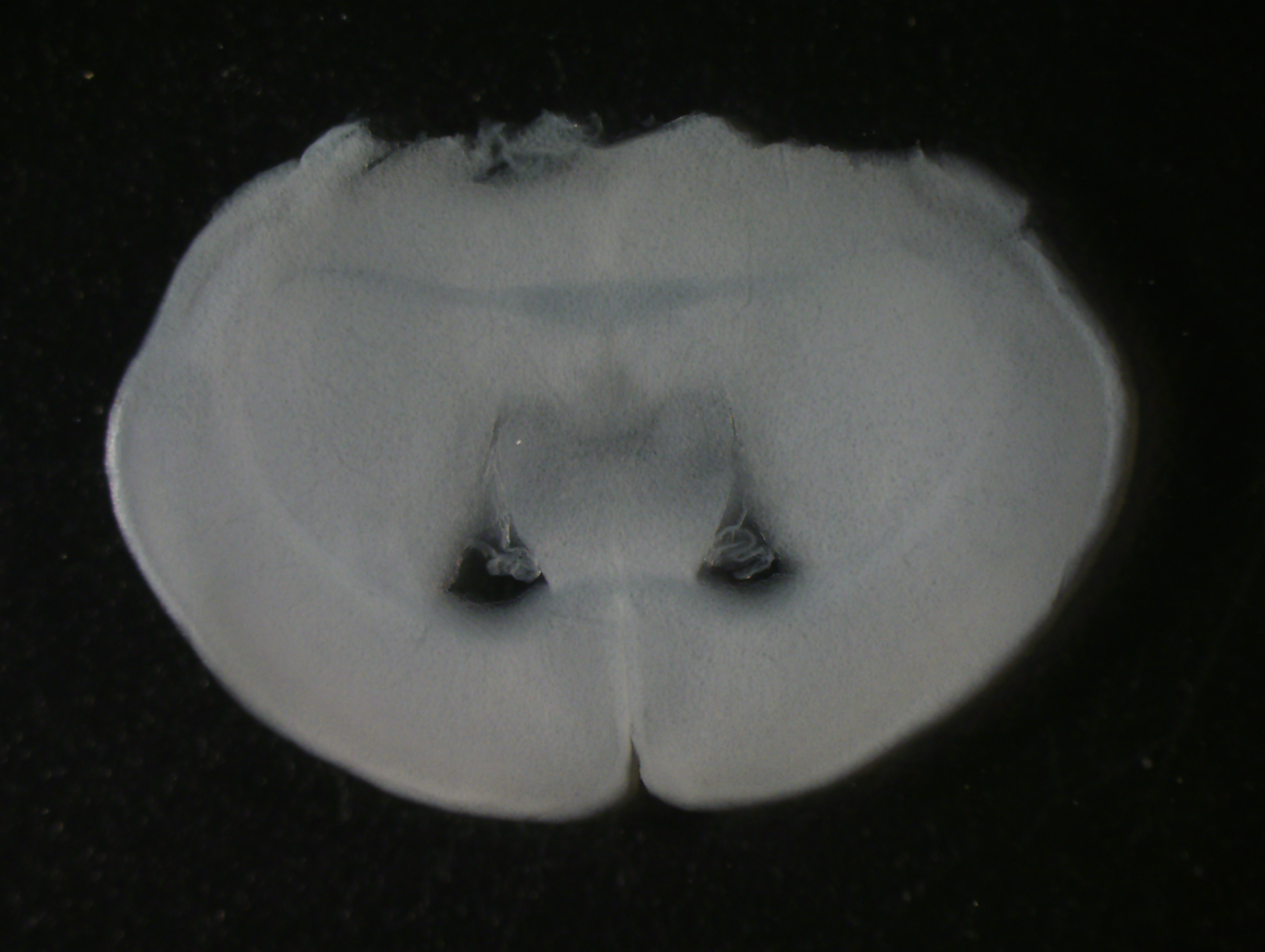

Supplement: Supplementary file 12 — Source data Fig. 4 [file 44319_2025_671_MOESM12_ESM.zip › SD figure 4/4G WT 2.tif]

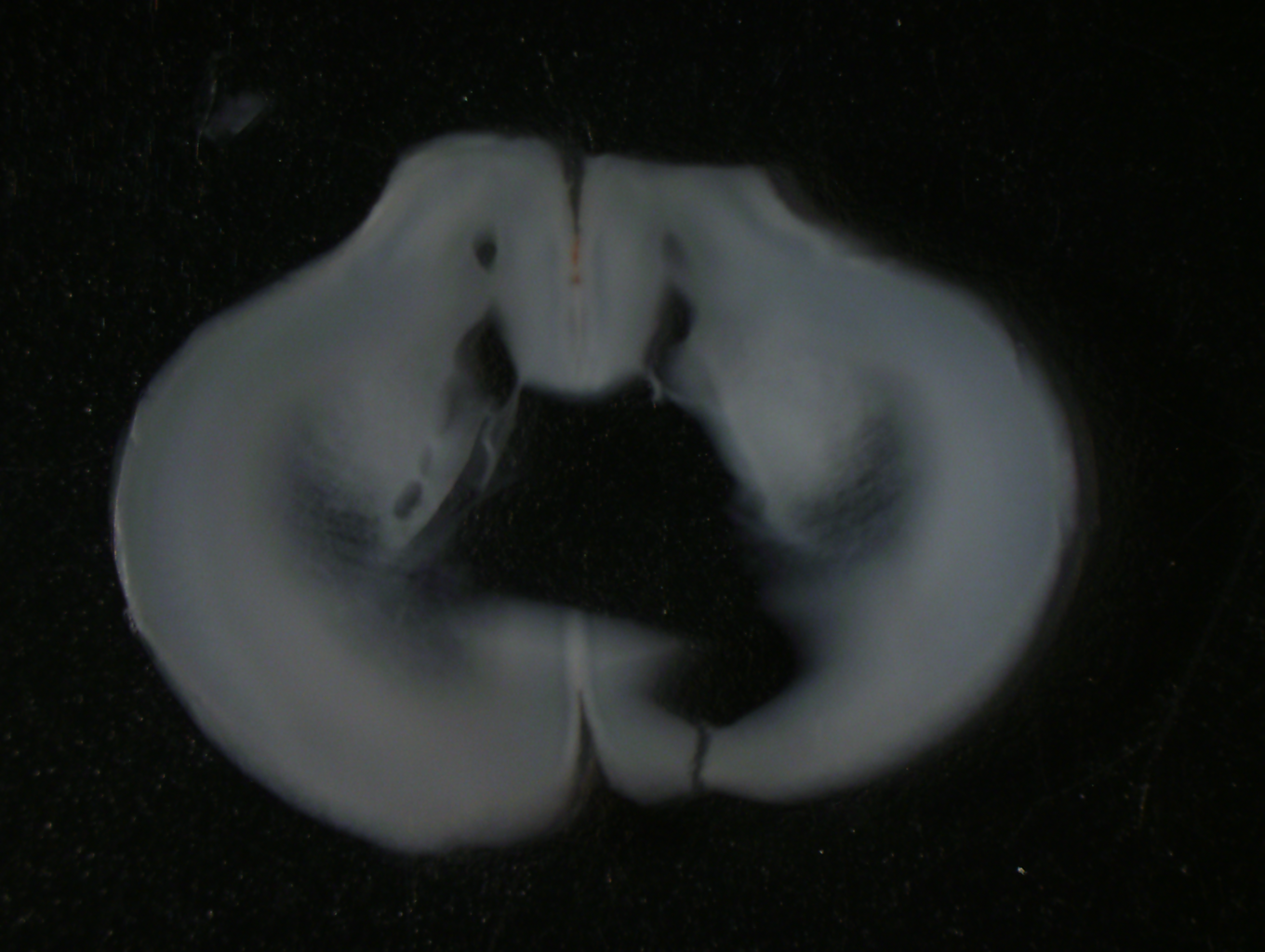

Supplement: Supplementary file 12 — Source data Fig. 4 [file 44319_2025_671_MOESM12_ESM.zip › SD figure 4/4G KO 1.tif]

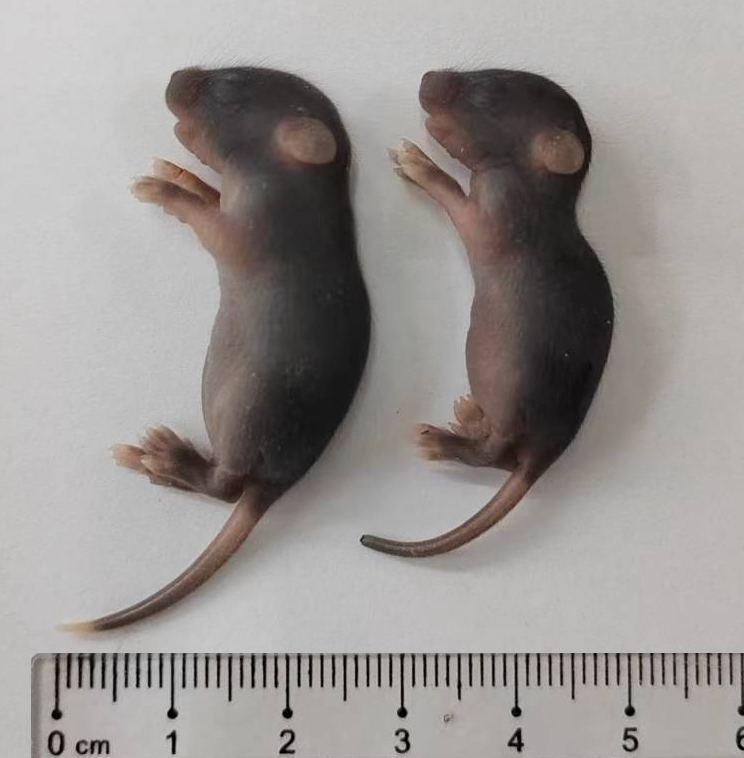

Supplement: Supplementary file 12 — Source data Fig. 4 [file 44319_2025_671_MOESM12_ESM.zip › SD figure 4/4E σ░ÅΘ╝á.tif]

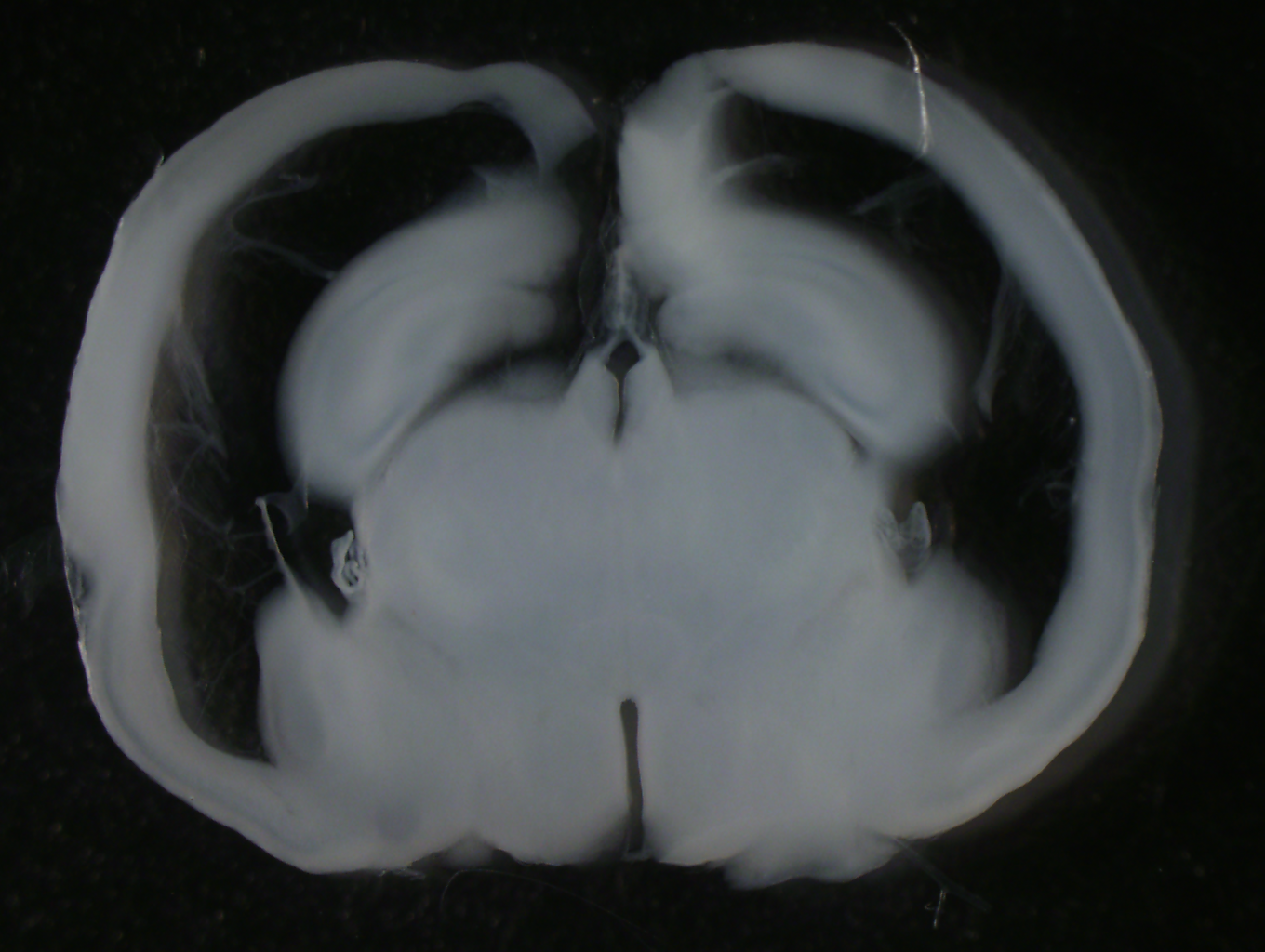

Supplement: Supplementary file 12 — Source data Fig. 4 [file 44319_2025_671_MOESM12_ESM.zip › SD figure 4/4G KO 3.tif]

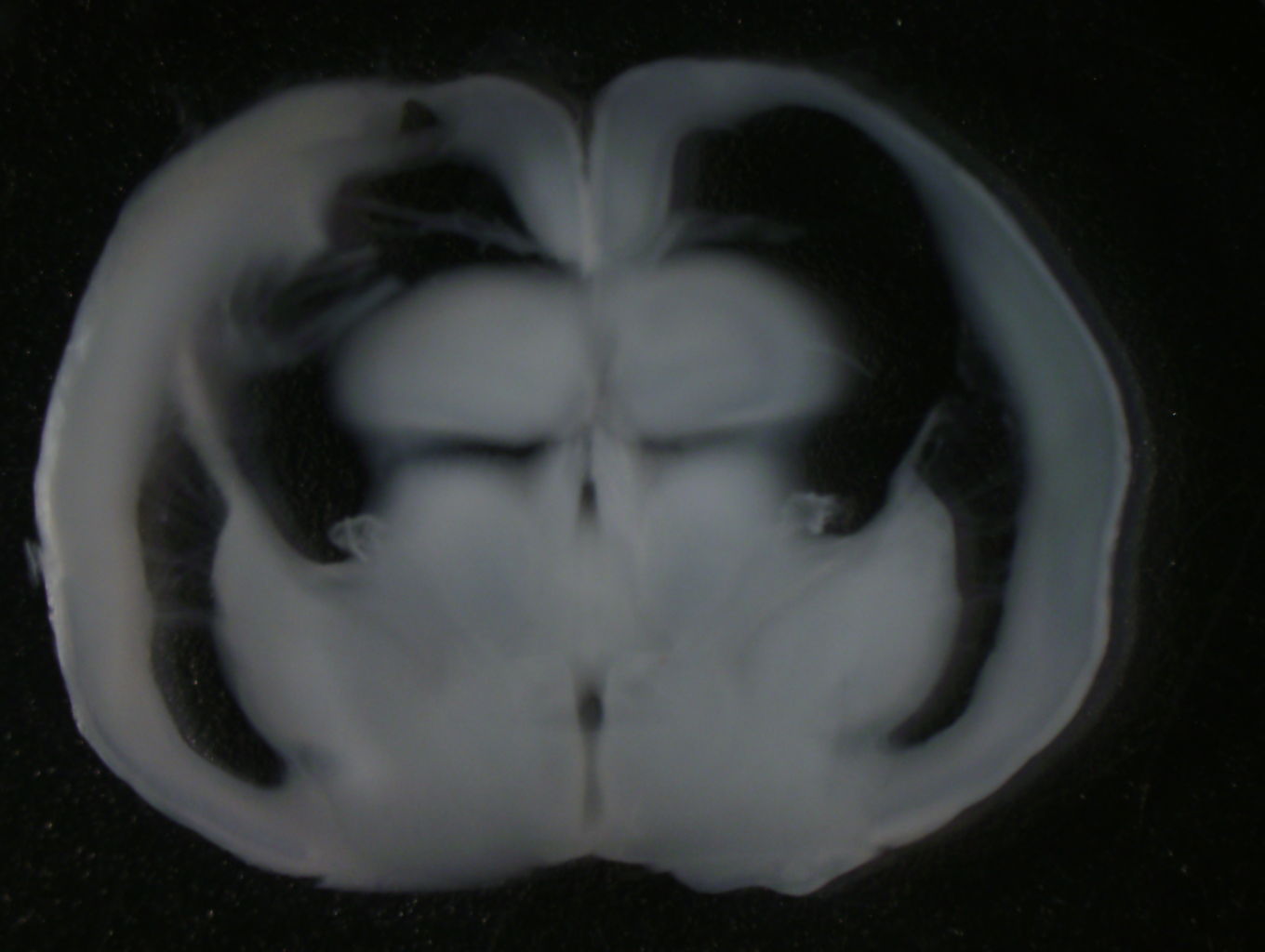

Supplement: Supplementary file 12 — Source data Fig. 4 [file 44319_2025_671_MOESM12_ESM.zip › SD figure 4/4G KO 2.tif]

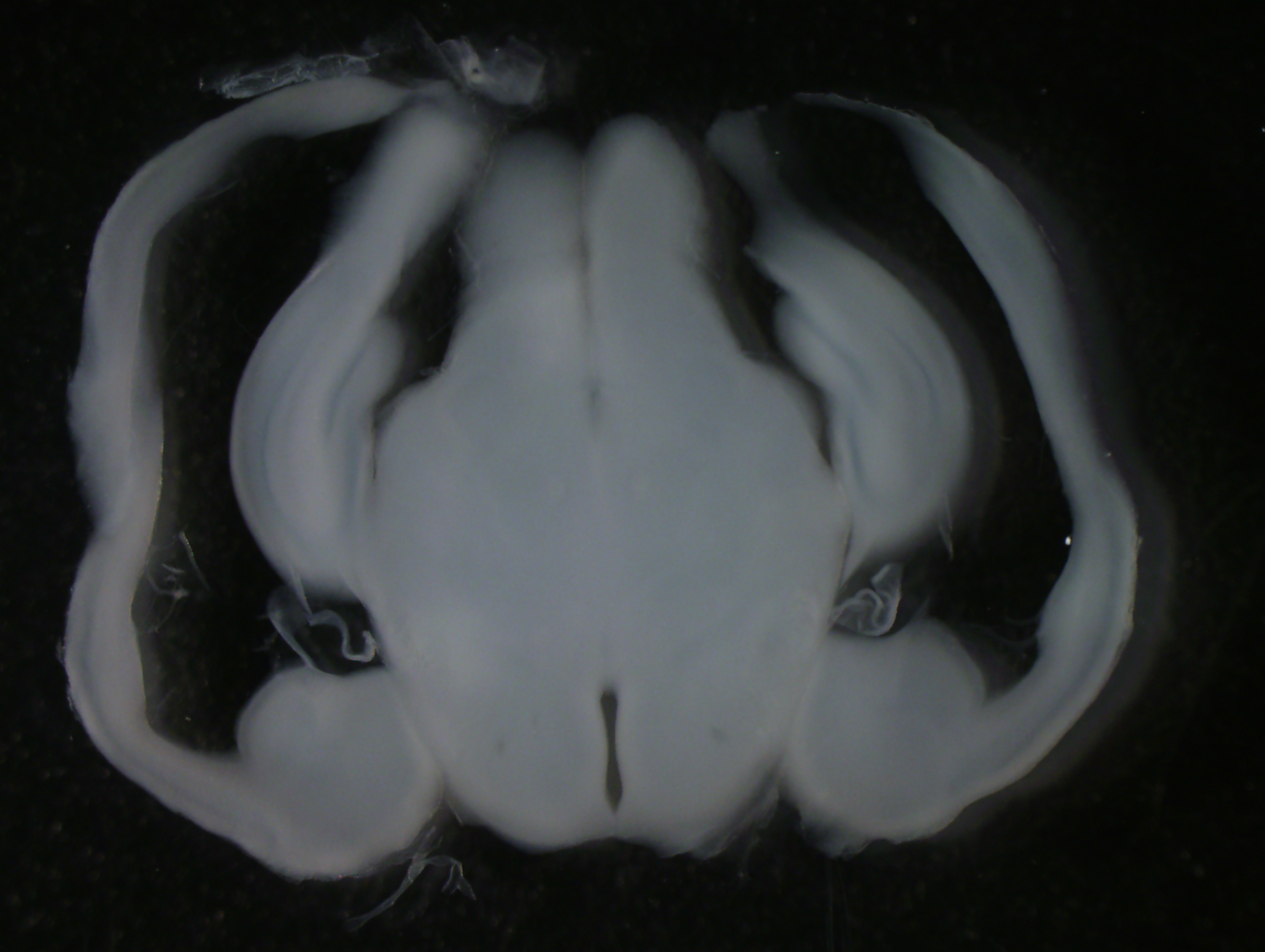

Supplement: Supplementary file 12 — Source data Fig. 4 [file 44319_2025_671_MOESM12_ESM.zip › SD figure 4/4G KO 4.tif]

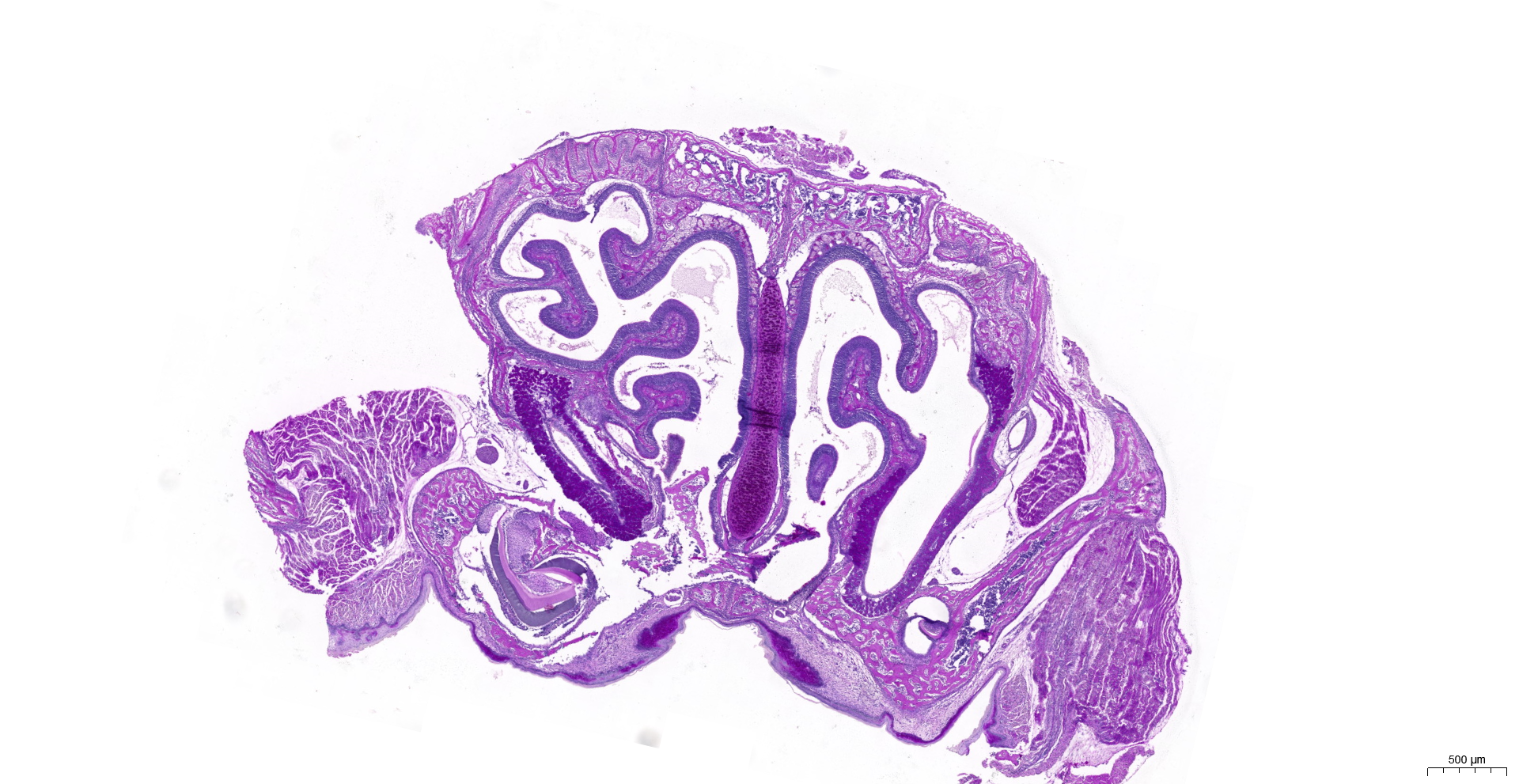

Supplement: Supplementary file 12 — Source data Fig. 4 [file 44319_2025_671_MOESM12_ESM.zip › SD figure 4/4H JHY-wt-40-2_2.tif]

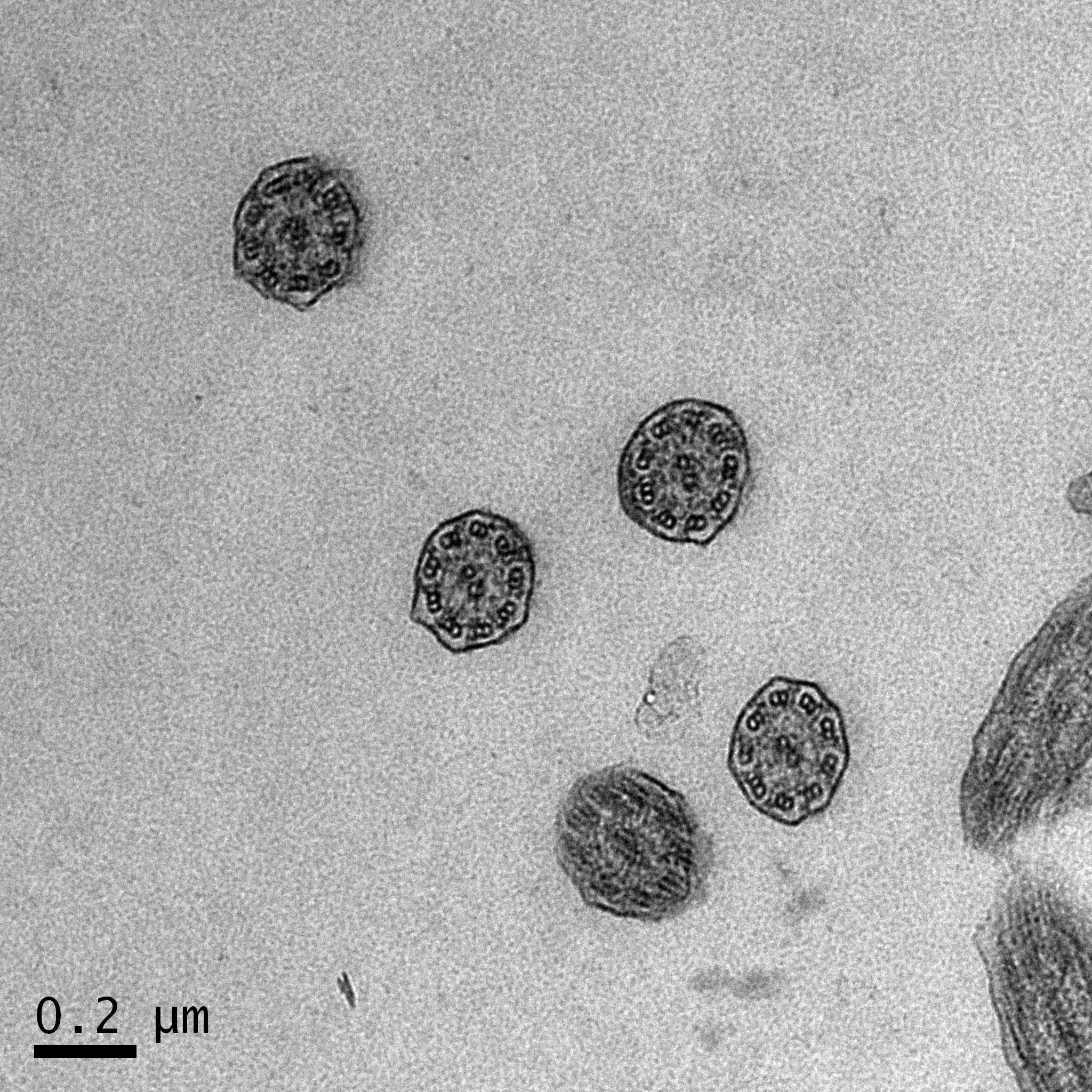

Supplement: Supplementary file 13 — Source data Fig. 5 [file 44319_2025_671_MOESM13_ESM.zip › SD figure 5/5G WT 9+2 upper.tif]

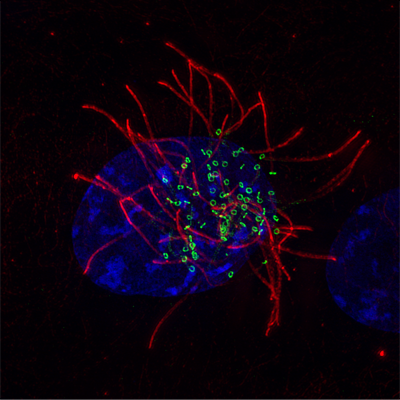

Supplement: Supplementary file 13 — Source data Fig. 5 [file 44319_2025_671_MOESM13_ESM.zip › SD figure 5/5B KO CEP164.tif]

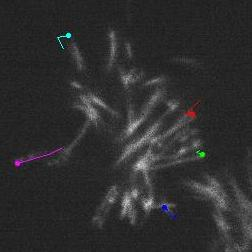

Supplement: Supplementary file 13 — Source data Fig. 5 [file 44319_2025_671_MOESM13_ESM.zip › SD figure 5/5D WT 20ms.tif]

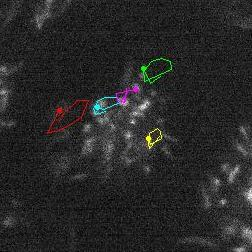

Supplement: Supplementary file 13 — Source data Fig. 5 [file 44319_2025_671_MOESM13_ESM.zip › SD figure 5/5D KO 80ms.tif]

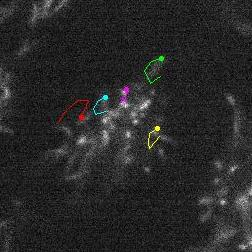

Supplement: Supplementary file 13 — Source data Fig. 5 [file 44319_2025_671_MOESM13_ESM.zip › SD figure 5/5D KO 40ms.tif]

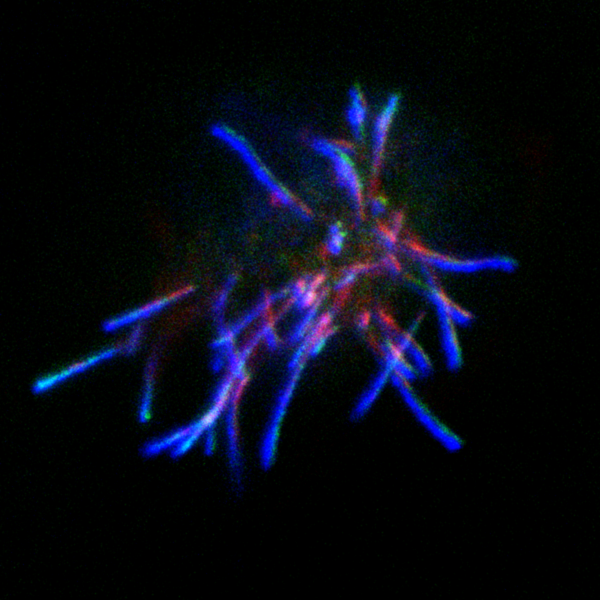

Supplement: Supplementary file 13 — Source data Fig. 5 [file 44319_2025_671_MOESM13_ESM.zip › SD figure 5/5F WT JHY HYDIN.tif]

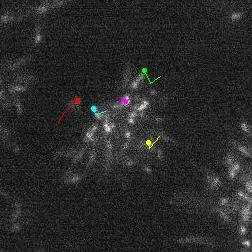

Supplement: Supplementary file 13 — Source data Fig. 5 [file 44319_2025_671_MOESM13_ESM.zip › SD figure 5/5D KO 20ms.tif]

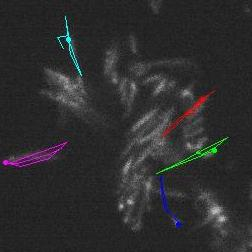

Supplement: Supplementary file 13 — Source data Fig. 5 [file 44319_2025_671_MOESM13_ESM.zip › SD figure 5/5D WT 80ms.tif]

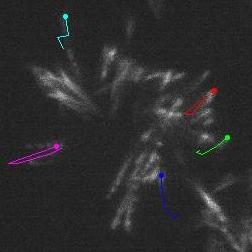

Supplement: Supplementary file 13 — Source data Fig. 5 [file 44319_2025_671_MOESM13_ESM.zip › SD figure 5/5D WT 40ms.tif]

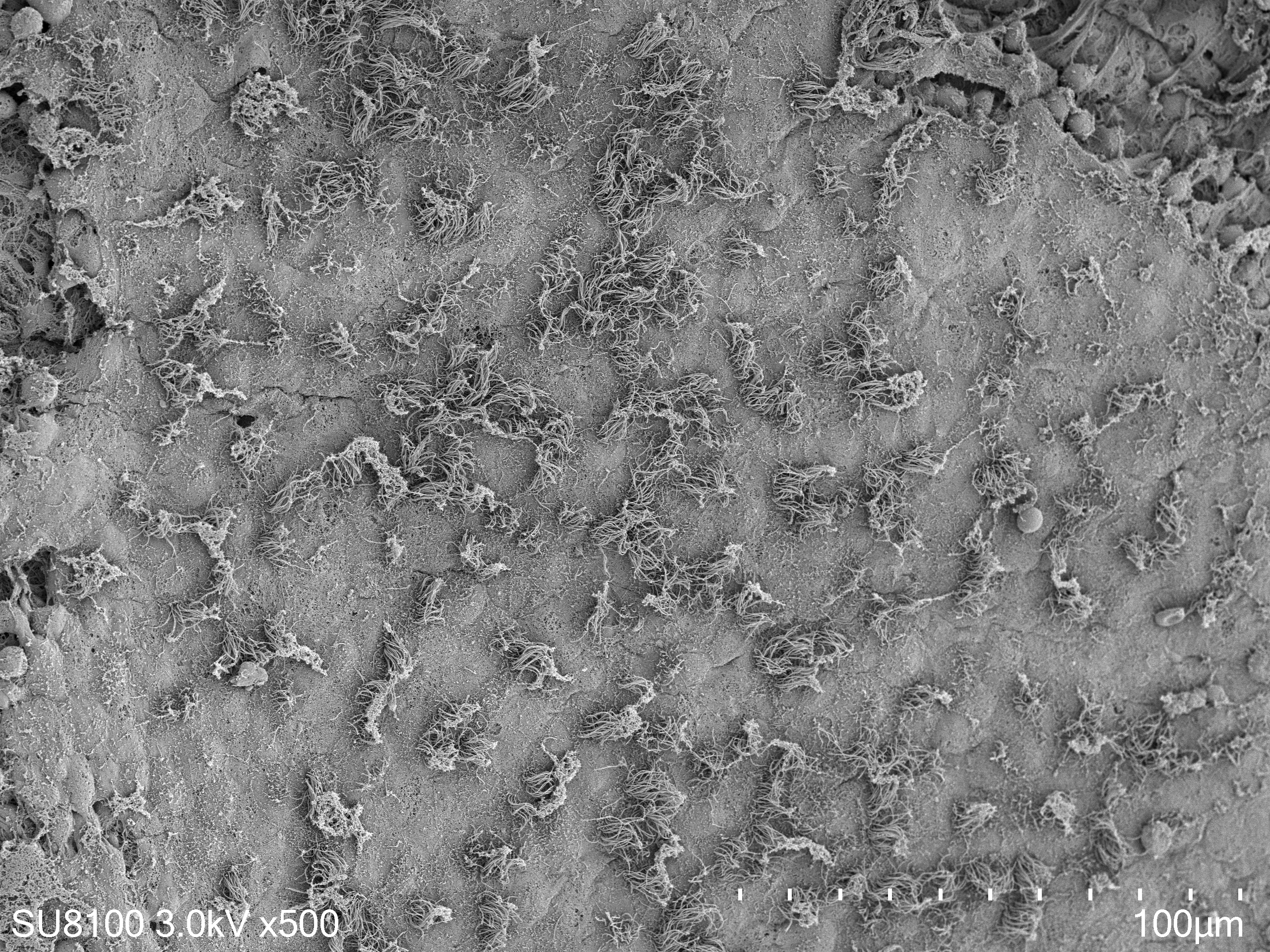

Supplement: Supplementary file 13 — Source data Fig. 5 [file 44319_2025_671_MOESM13_ESM.zip › SD figure 5/5A WT SEM P5 Left.tif]

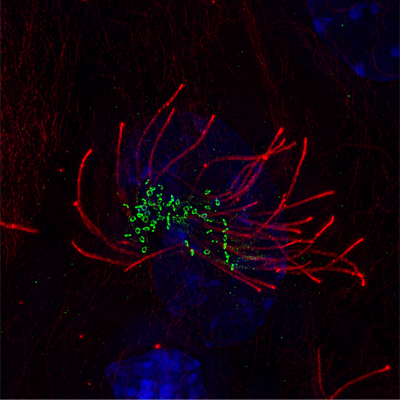

Supplement: Supplementary file 13 — Source data Fig. 5 [file 44319_2025_671_MOESM13_ESM.zip › SD figure 5/5B WT CEP164.tif]

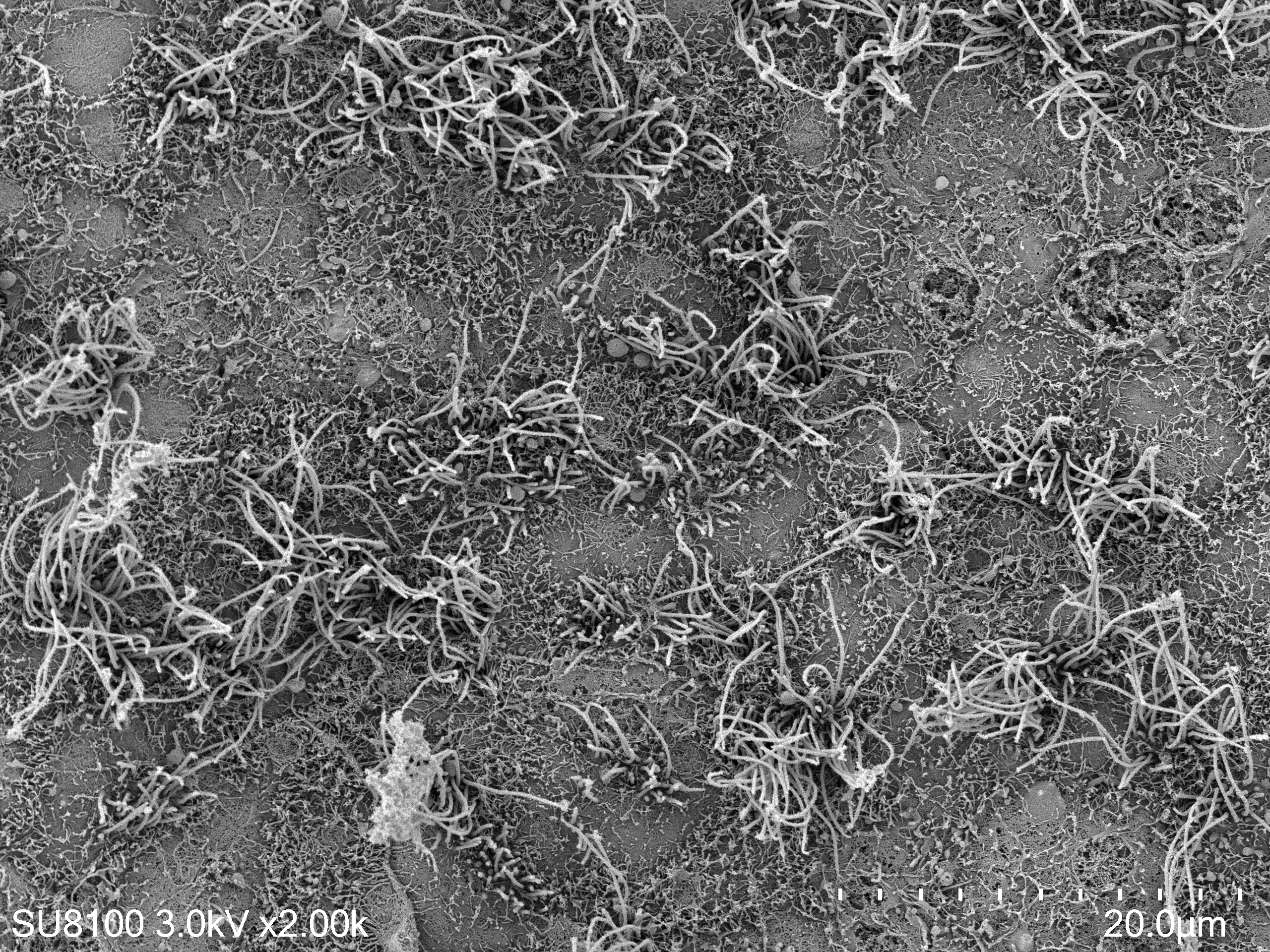

Supplement: Supplementary file 13 — Source data Fig. 5 [file 44319_2025_671_MOESM13_ESM.zip › SD figure 5/5A KO SEM P5 Right.tif]

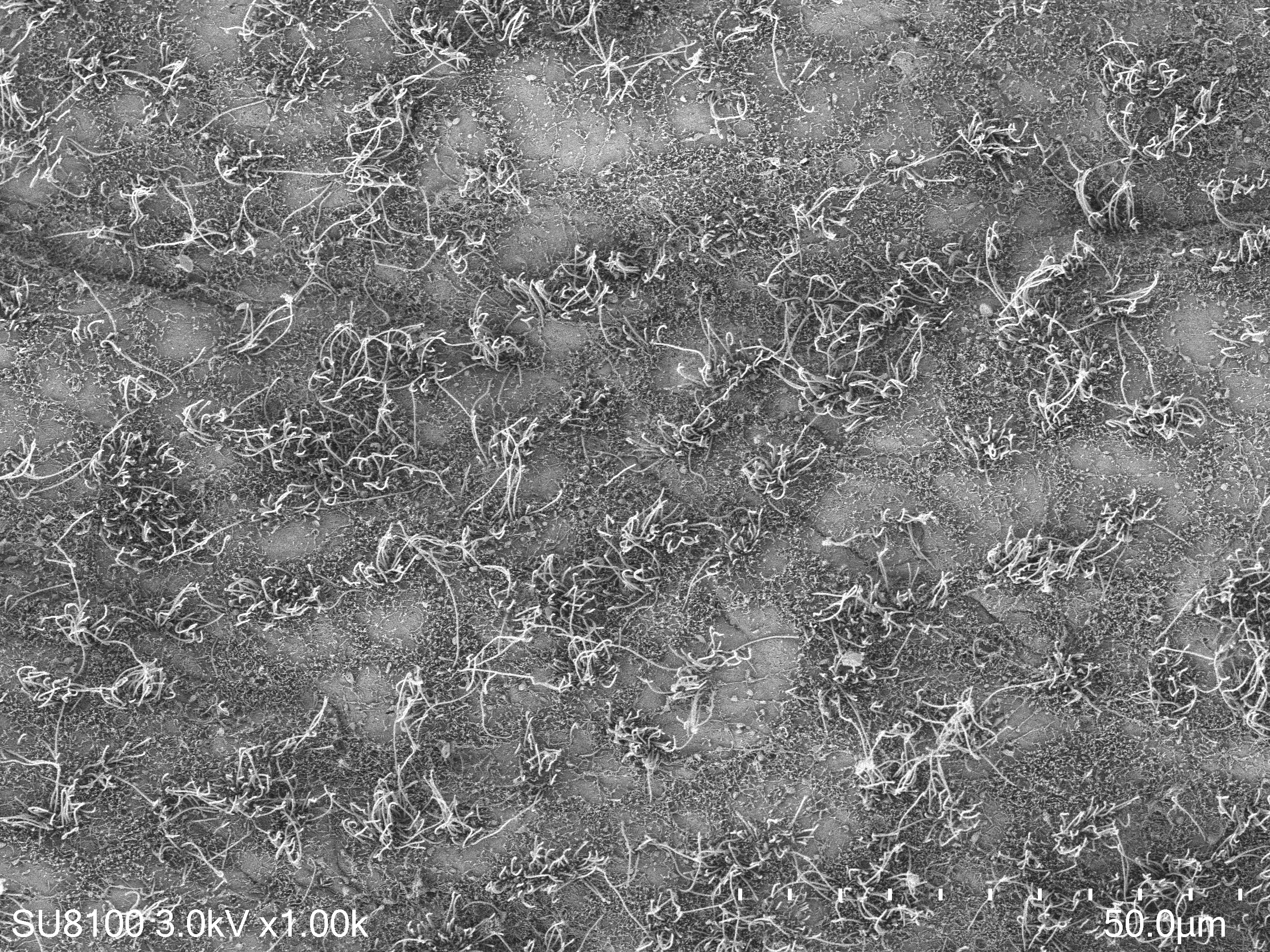

Supplement: Supplementary file 13 — Source data Fig. 5 [file 44319_2025_671_MOESM13_ESM.zip › SD figure 5/5A KO SEM P10 Left.tif]

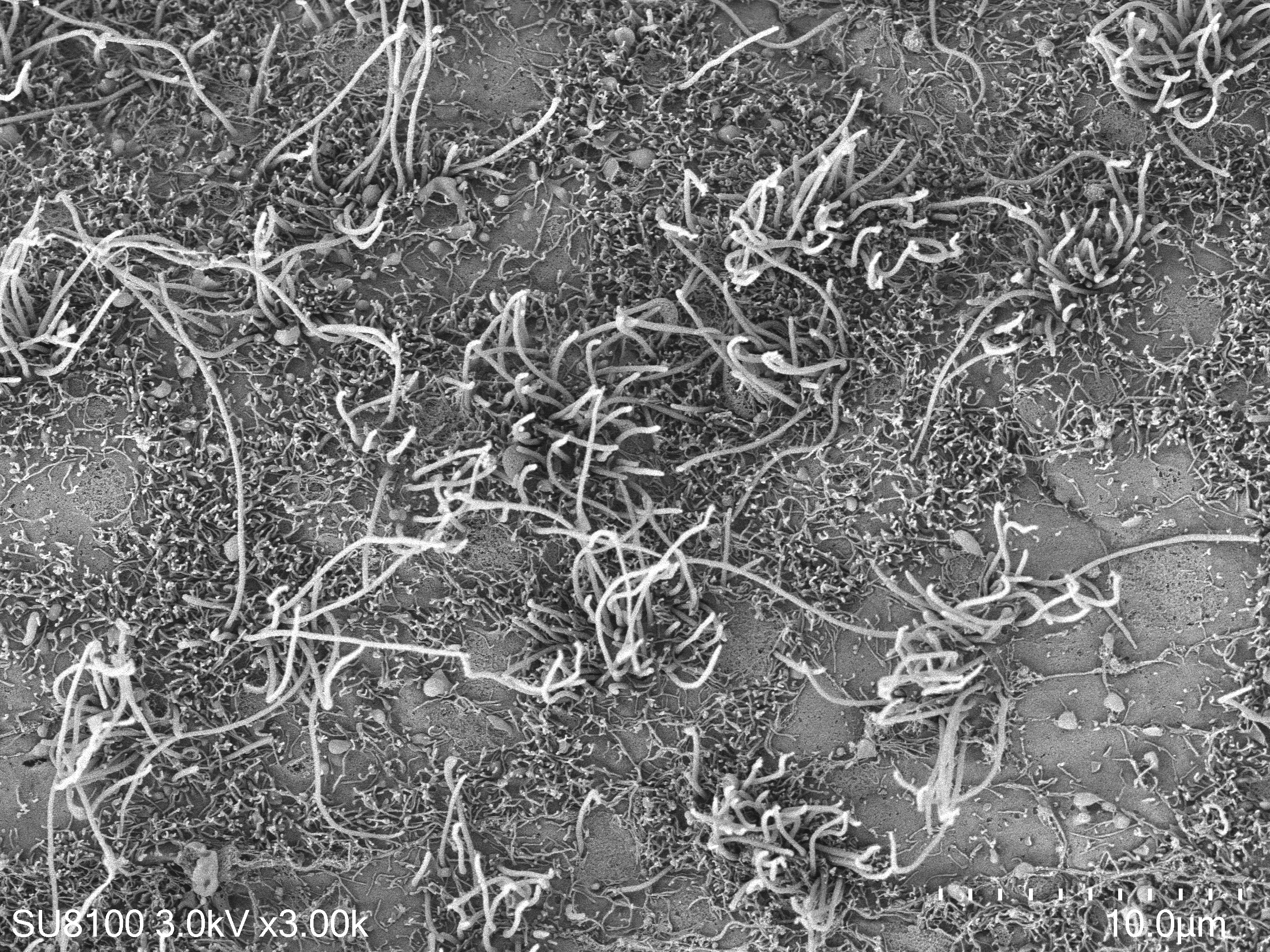

Supplement: Supplementary file 13 — Source data Fig. 5 [file 44319_2025_671_MOESM13_ESM.zip › SD figure 5/5A KO SEM P10 Right.tif]

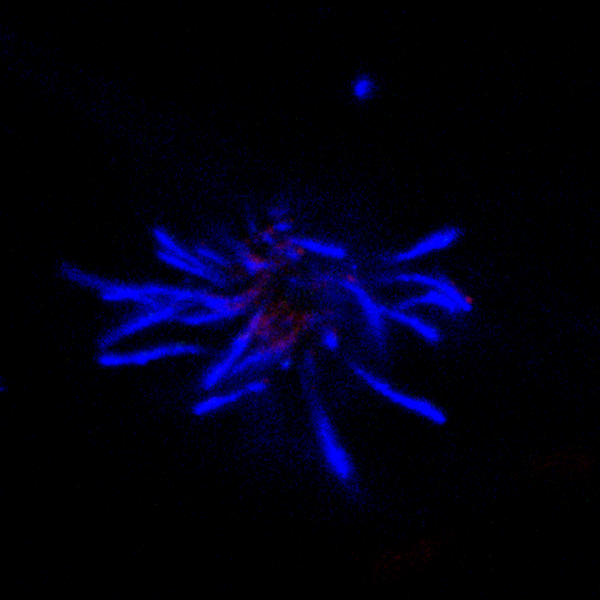

Supplement: Supplementary file 13 — Source data Fig. 5 [file 44319_2025_671_MOESM13_ESM.zip › SD figure 5/5F KO JHY HYDIN.tif]

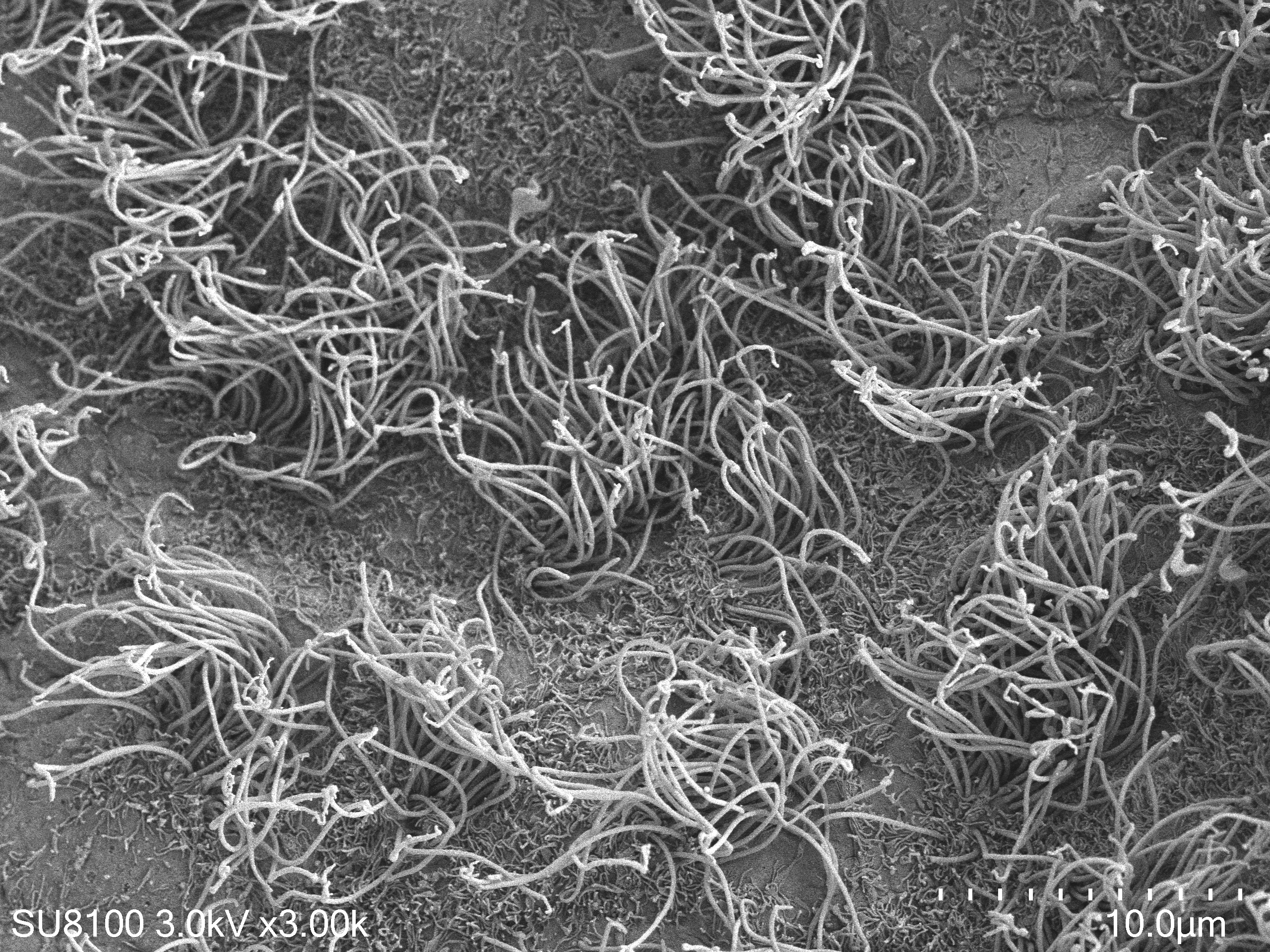

Supplement: Supplementary file 13 — Source data Fig. 5 [file 44319_2025_671_MOESM13_ESM.zip › SD figure 5/5A WT SEM P10 Right.tif]

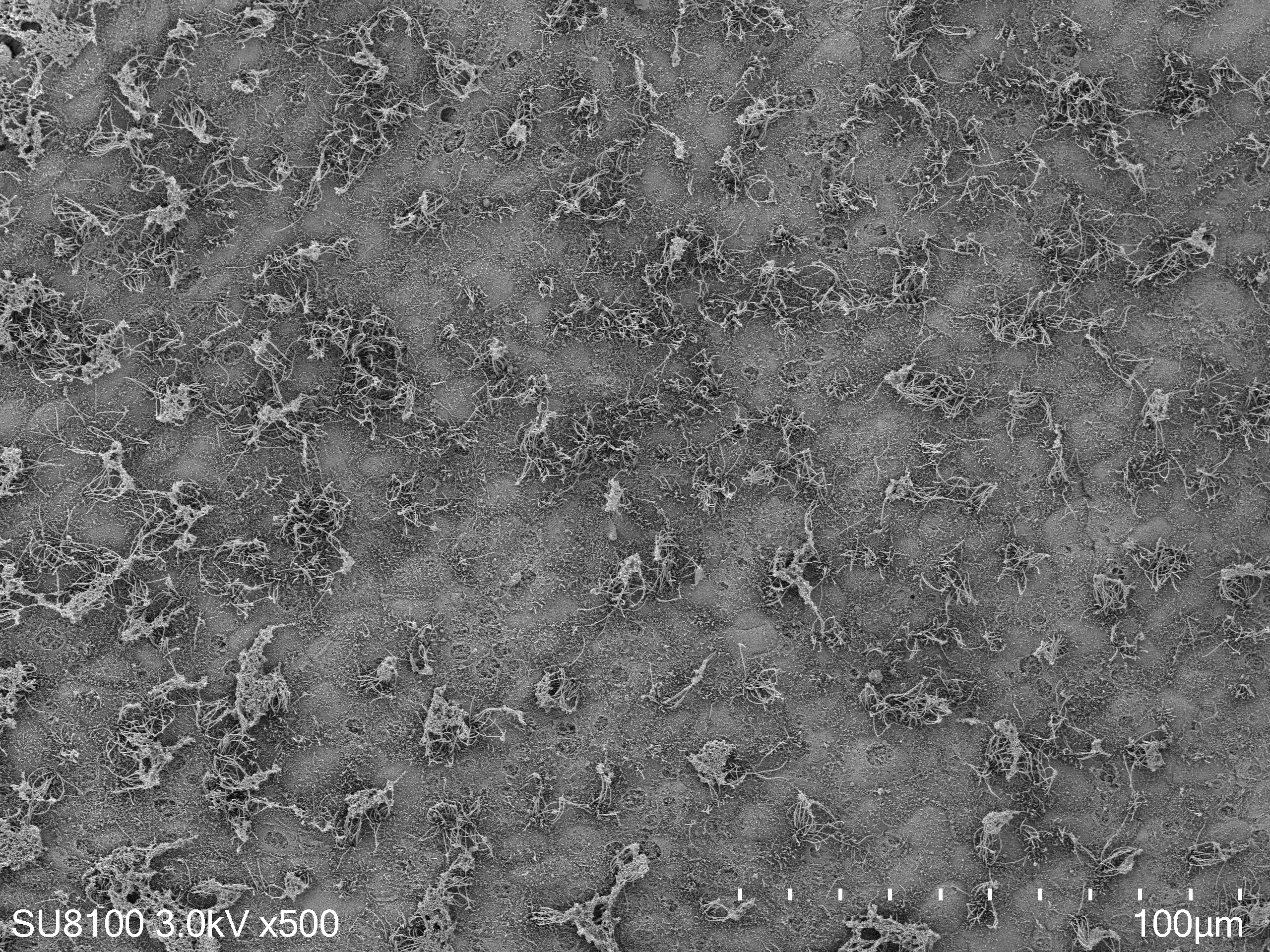

Supplement: Supplementary file 13 — Source data Fig. 5 [file 44319_2025_671_MOESM13_ESM.zip › SD figure 5/5A KO SEM P5 Left.tif]

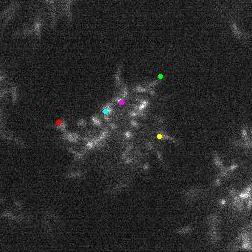

Supplement: Supplementary file 13 — Source data Fig. 5 [file 44319_2025_671_MOESM13_ESM.zip › SD figure 5/5D KO 0ms.tif]

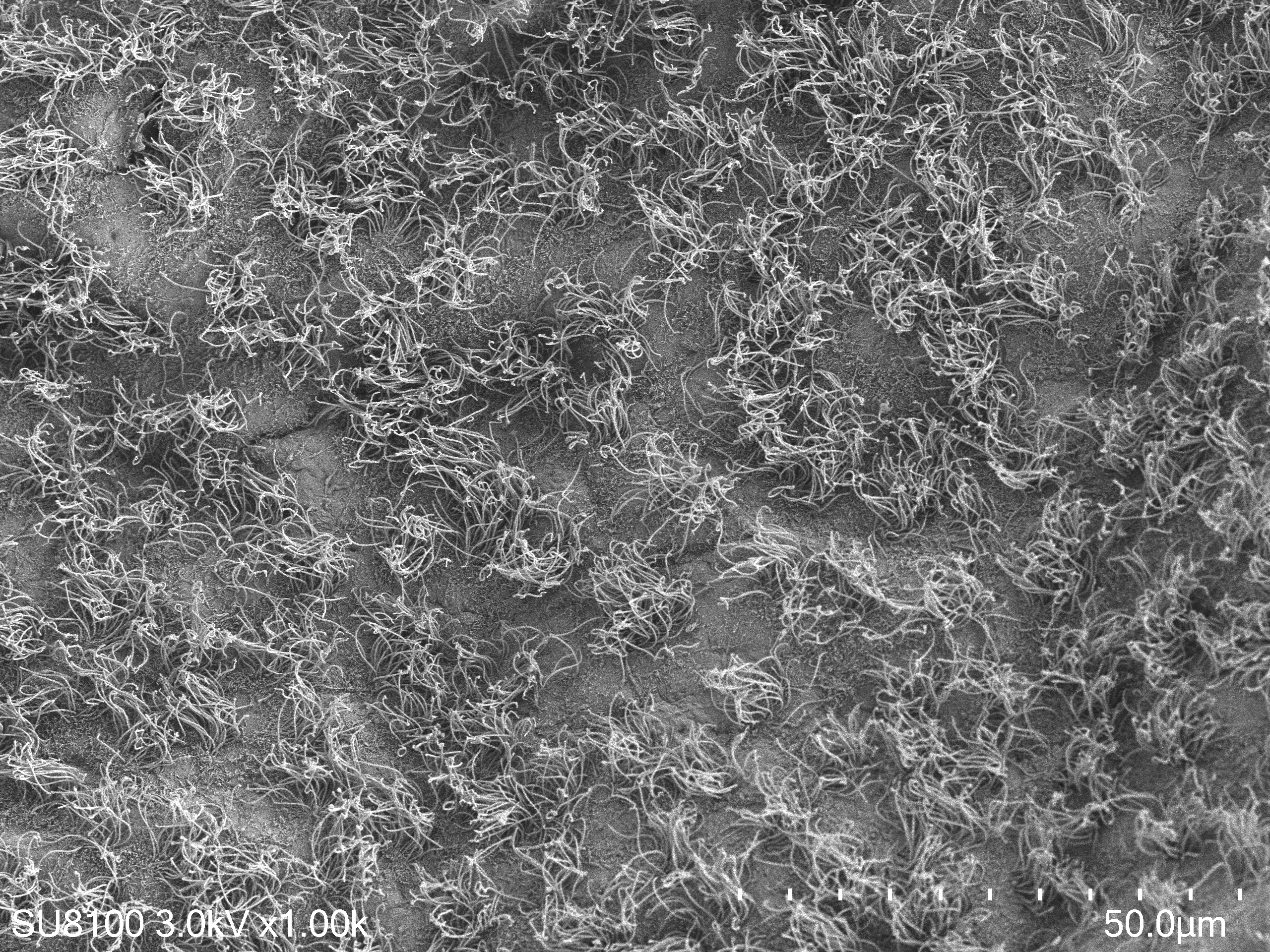

Supplement: Supplementary file 13 — Source data Fig. 5 [file 44319_2025_671_MOESM13_ESM.zip › SD figure 5/5A WT SEM P10 Left.tif]

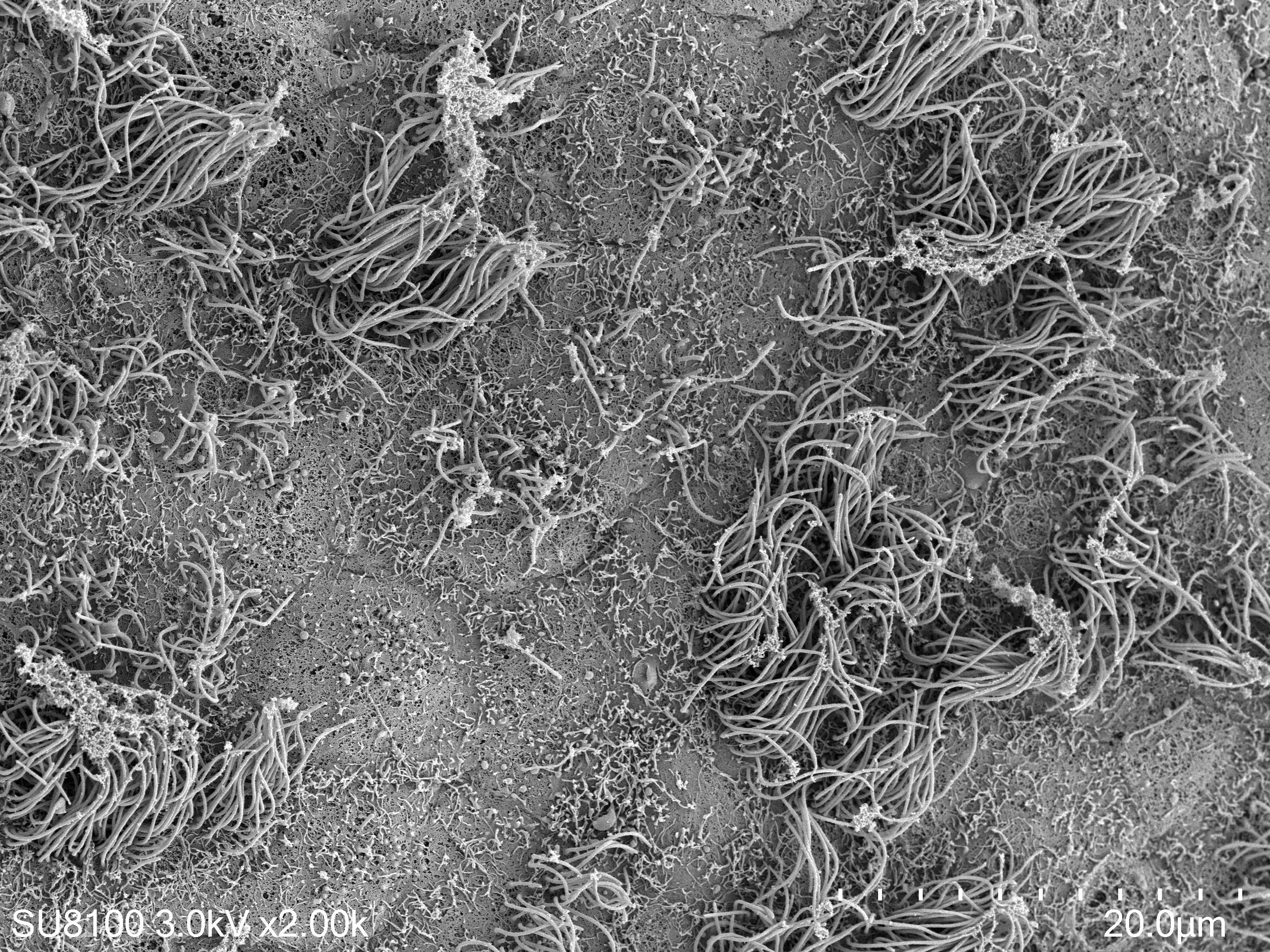

Supplement: Supplementary file 13 — Source data Fig. 5 [file 44319_2025_671_MOESM13_ESM.zip › SD figure 5/5A WT SEM P5 Right.tif]

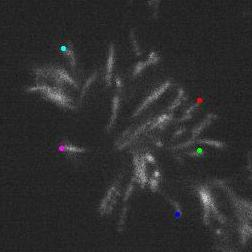

Supplement: Supplementary file 13 — Source data Fig. 5 [file 44319_2025_671_MOESM13_ESM.zip › SD figure 5/5D WT 0ms.tif]

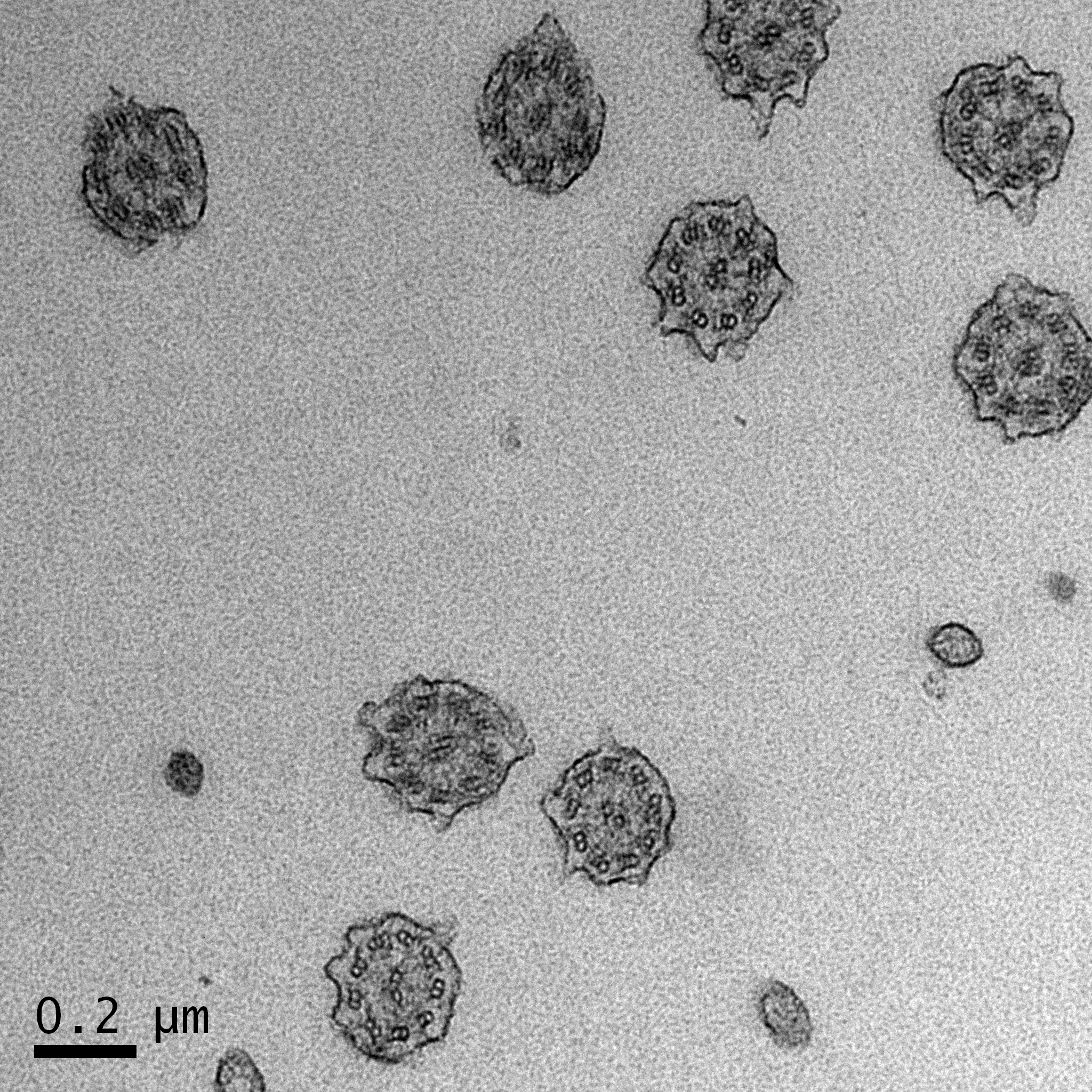

Supplement: Supplementary file 13 — Source data Fig. 5 [file 44319_2025_671_MOESM13_ESM.zip › SD figure 5/5G WT 9+2 bottom.tif]

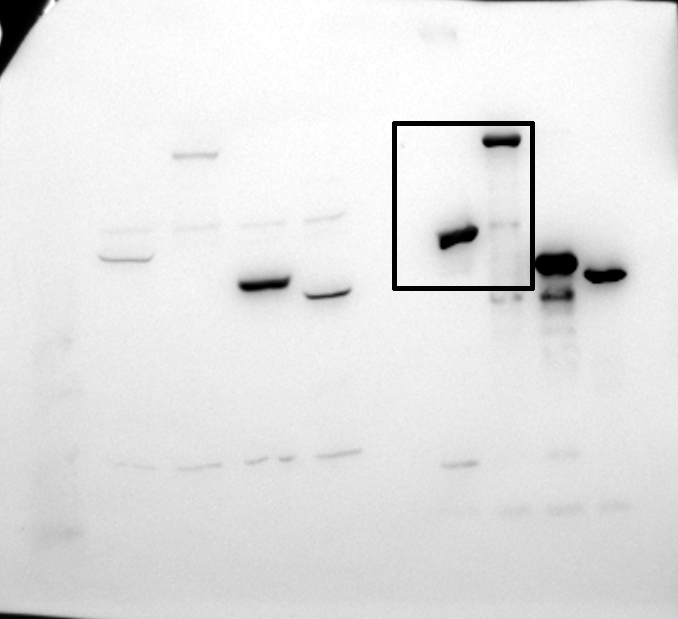

Supplement: Supplementary file 14 — Source data Fig. 6 [file 44319_2025_671_MOESM14_ESM.zip › SD figure 6/6H FLAG IP.tif]

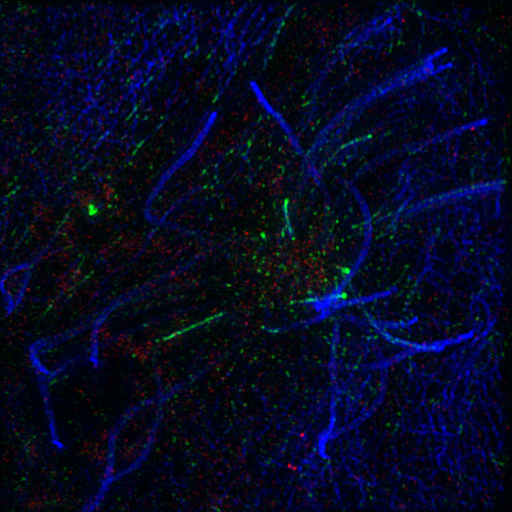

Supplement: Supplementary file 14 — Source data Fig. 6 [file 44319_2025_671_MOESM14_ESM.zip › SD figure 6/6C KO WDR47 SIM.tif]

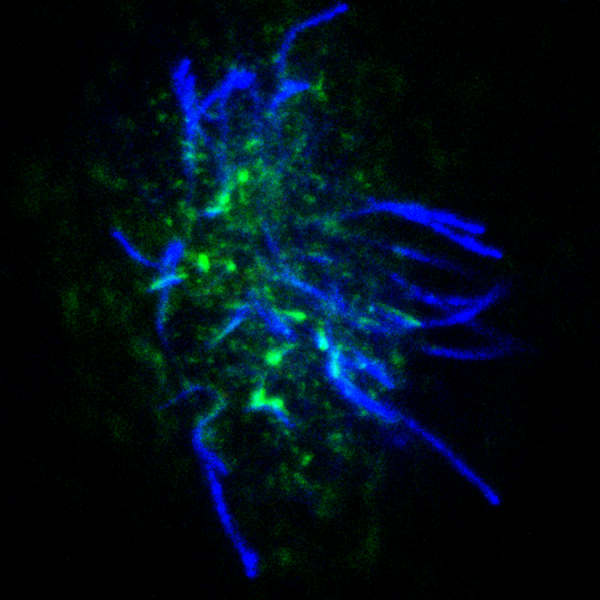

Supplement: Supplementary file 14 — Source data Fig. 6 [file 44319_2025_671_MOESM14_ESM.zip › SD figure 6/6A KO WDR47 confocal.tif]

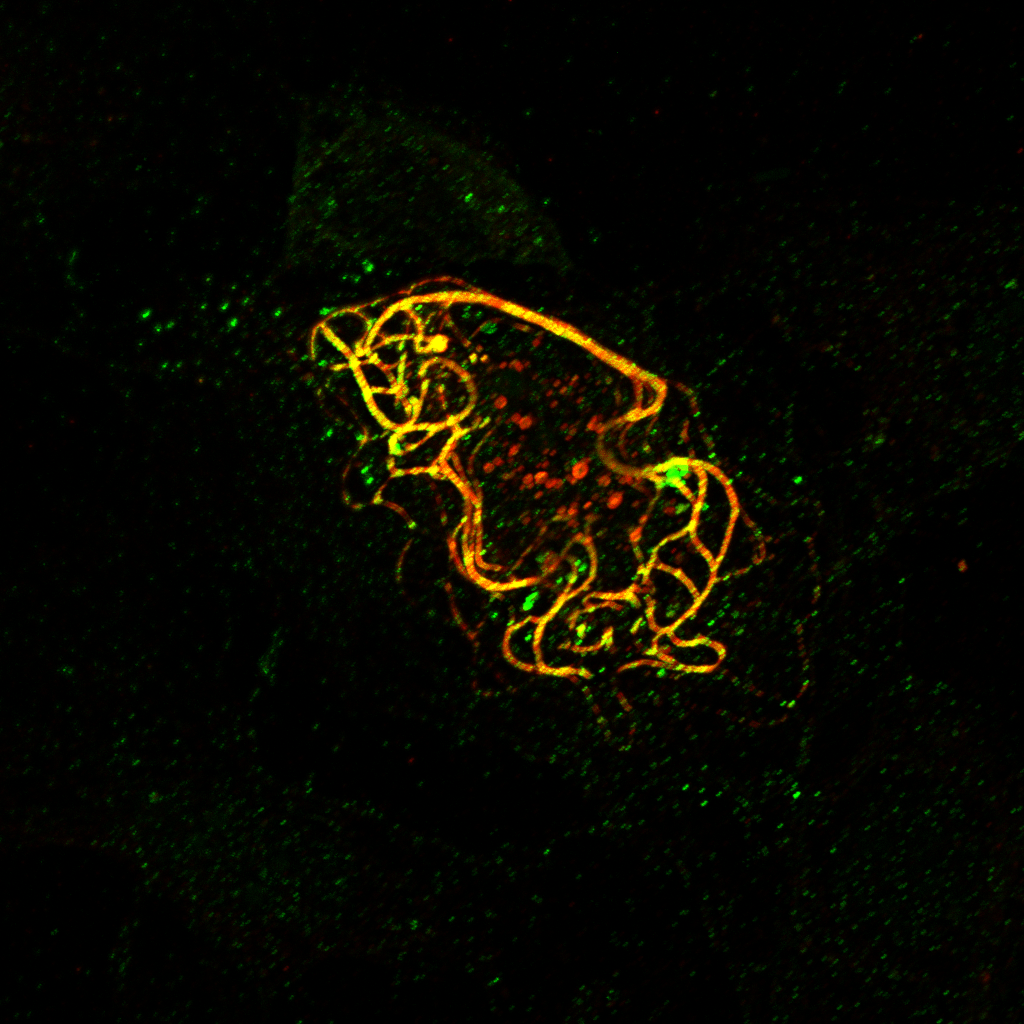

Supplement: Supplementary file 14 — Source data Fig. 6 [file 44319_2025_671_MOESM14_ESM.zip › SD figure 6/6E GFP-WDR47 FLAG-SPEF1.tif]

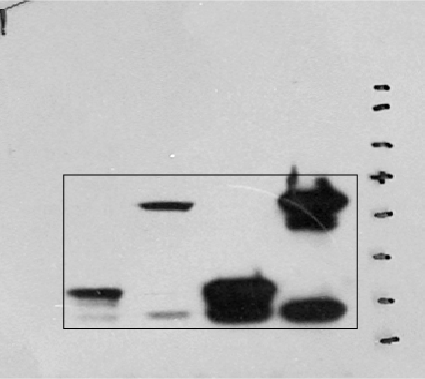

Supplement: Supplementary file 14 — Source data Fig. 6 [file 44319_2025_671_MOESM14_ESM.zip › SD figure 6/6F IP a-GFP.tif]

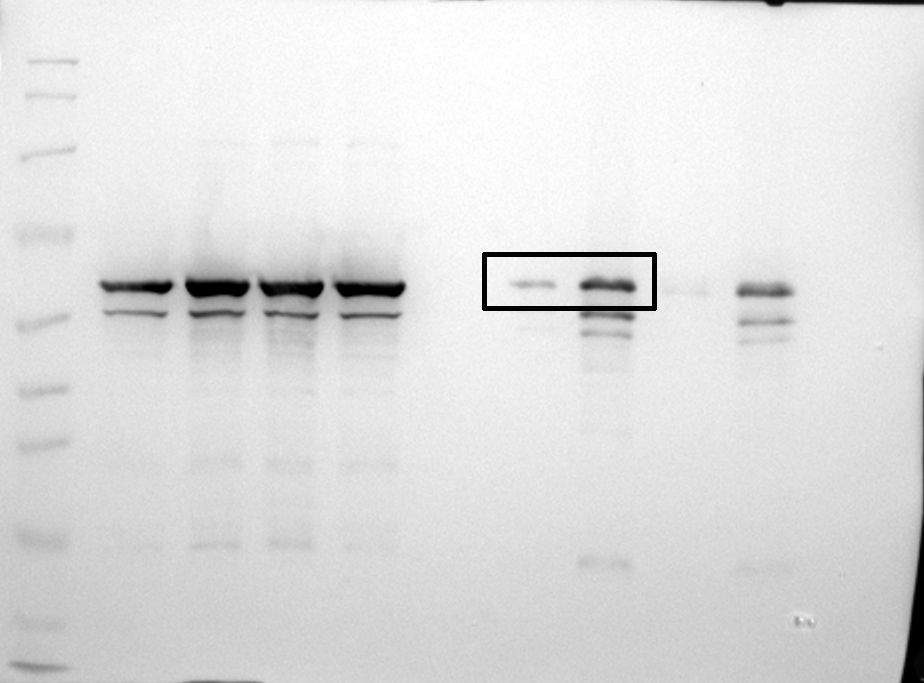

Supplement: Supplementary file 14 — Source data Fig. 6 [file 44319_2025_671_MOESM14_ESM.zip › SD figure 6/6H GFP IP.tif]

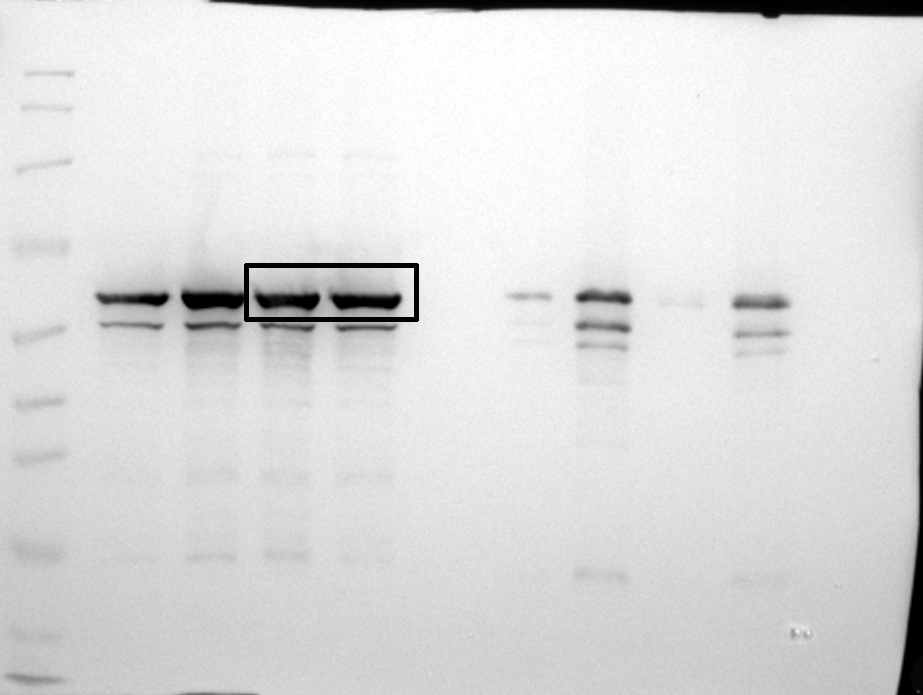

Supplement: Supplementary file 14 — Source data Fig. 6 [file 44319_2025_671_MOESM14_ESM.zip › SD figure 6/6I GFP input.tif]

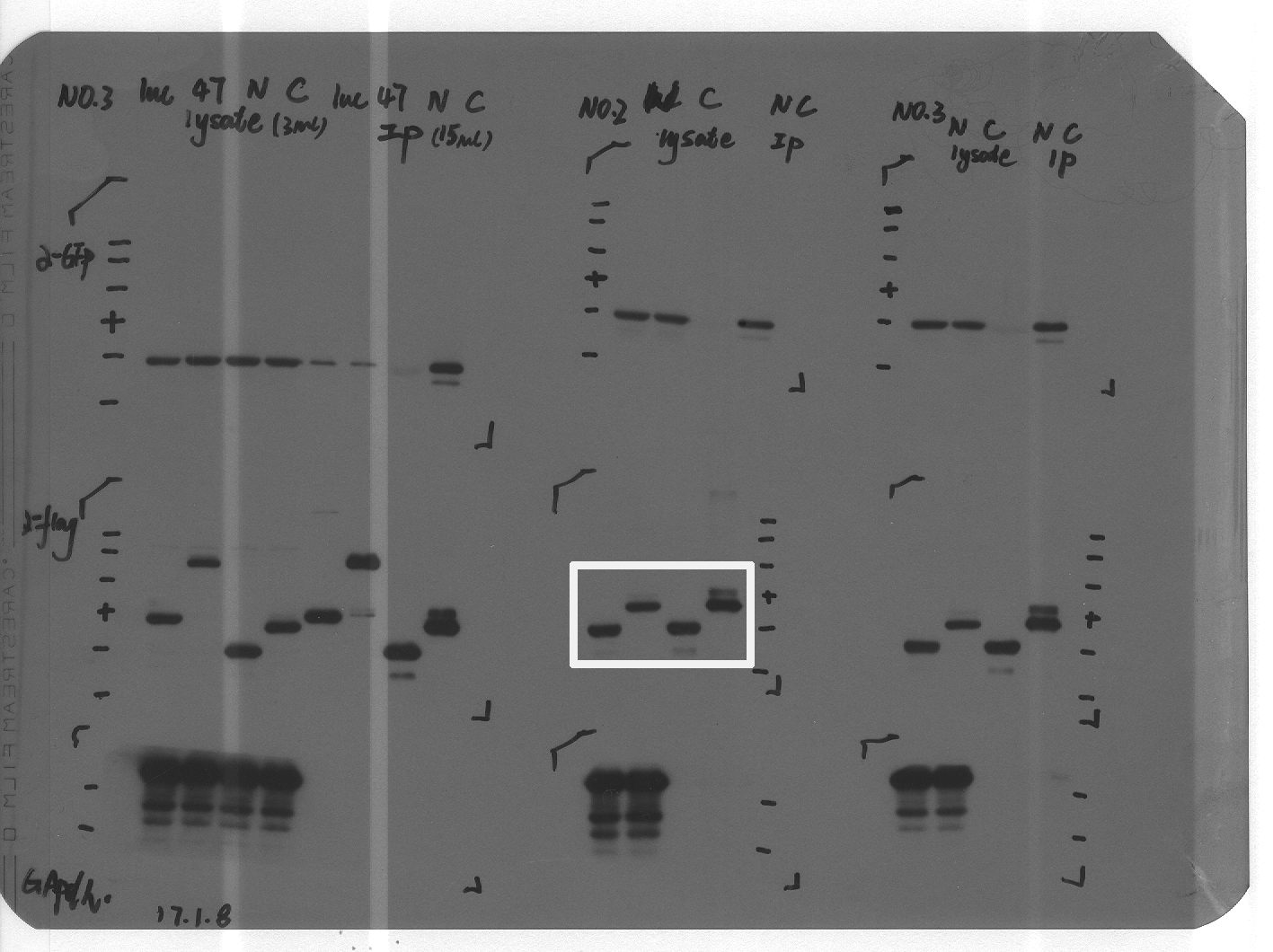

Supplement: Supplementary file 14 — Source data Fig. 6 [file 44319_2025_671_MOESM14_ESM.zip › SD figure 6/6G FLAG.tif]

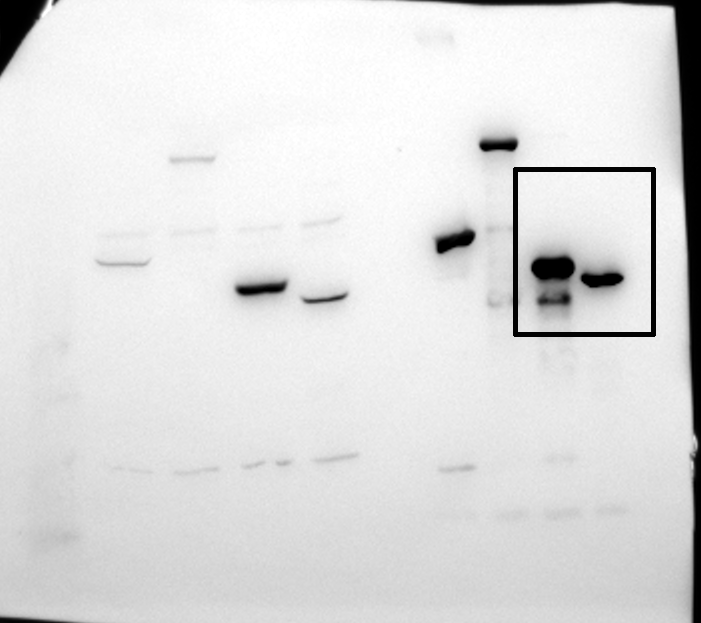

Supplement: Supplementary file 14 — Source data Fig. 6 [file 44319_2025_671_MOESM14_ESM.zip › SD figure 6/6I FLAG IP.tif]

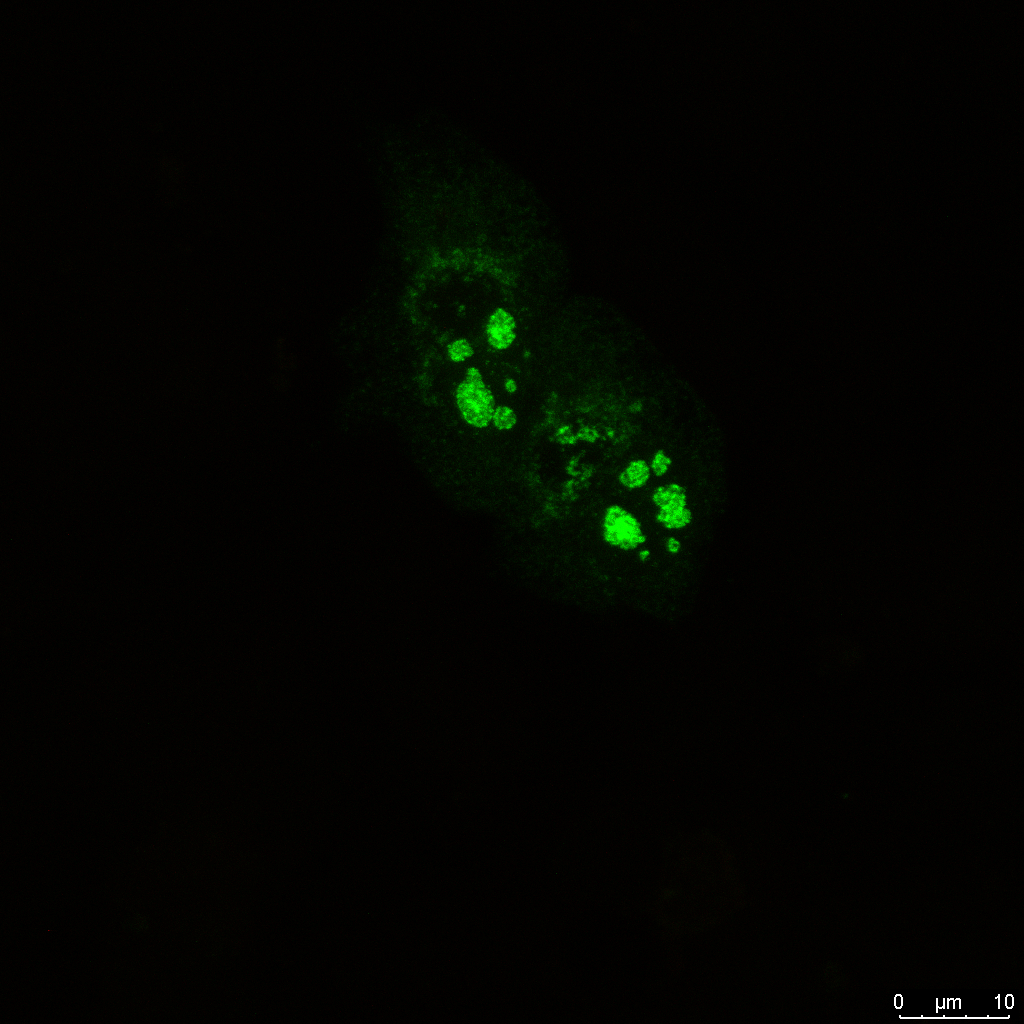

Supplement: Supplementary file 14 — Source data Fig. 6 [file 44319_2025_671_MOESM14_ESM.zip › SD figure 6/6D SNAP GFP-JHY.tif]

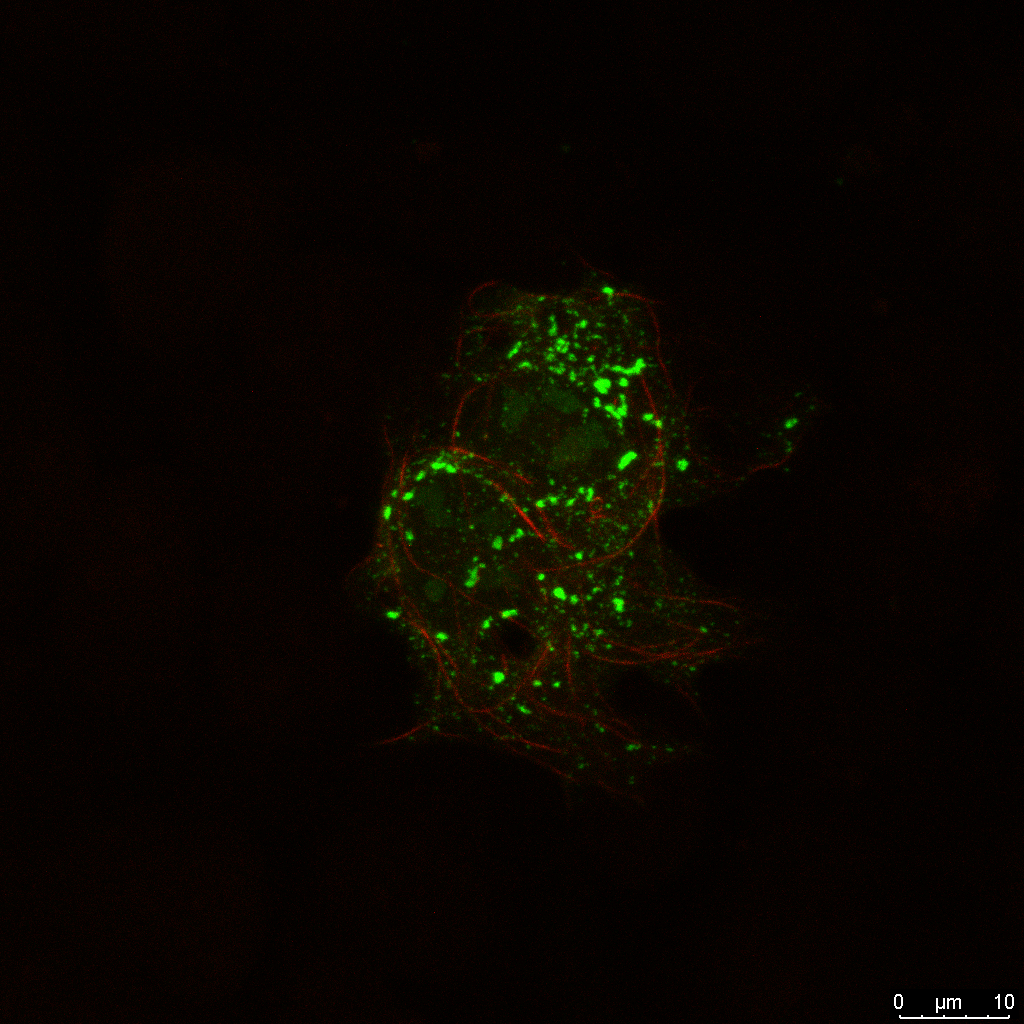

Supplement: Supplementary file 14 — Source data Fig. 6 [file 44319_2025_671_MOESM14_ESM.zip › SD figure 6/6D SNAP-CAMSAP1 GFP-JHY.tif]

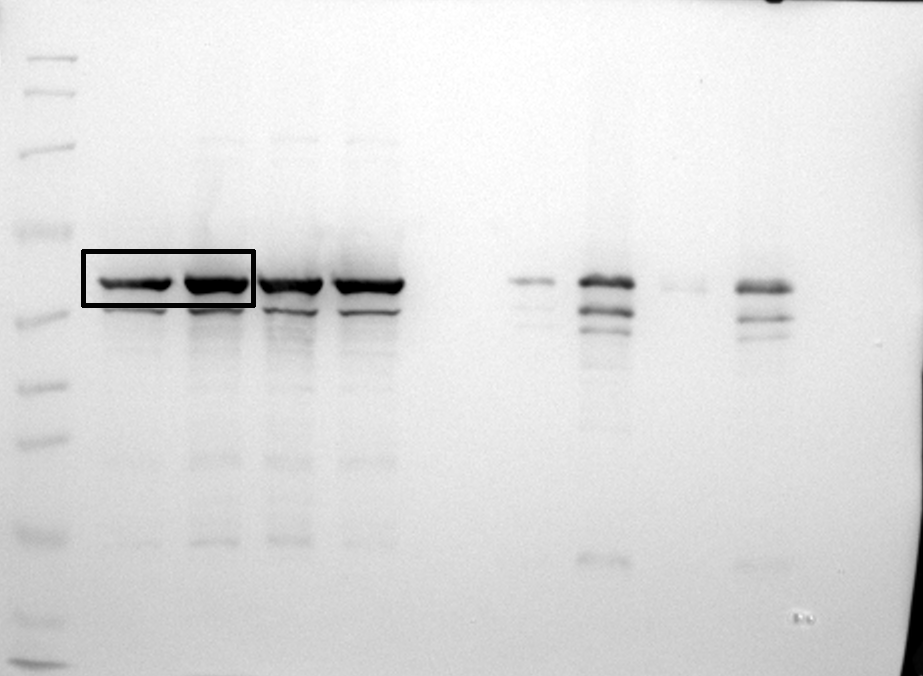

Supplement: Supplementary file 14 — Source data Fig. 6 [file 44319_2025_671_MOESM14_ESM.zip › SD figure 6/6H GFP input.tif]

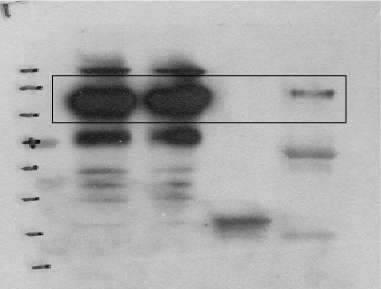

Supplement: Supplementary file 14 — Source data Fig. 6 [file 44319_2025_671_MOESM14_ESM.zip › SD figure 6/6F coIP a-Flag.tif]

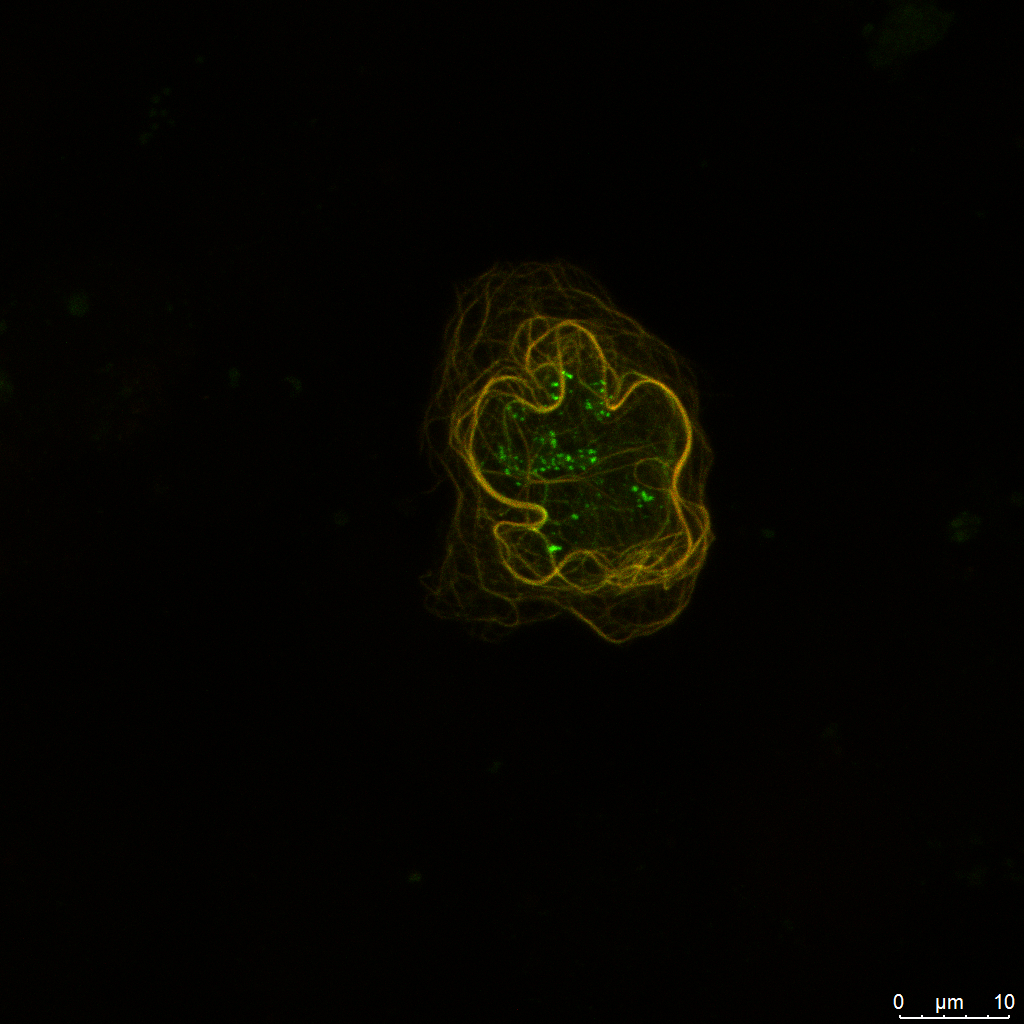

Supplement: Supplementary file 14 — Source data Fig. 6 [file 44319_2025_671_MOESM14_ESM.zip › SD figure 6/6D SNAP-SPEF1 GFP-JHY.tif]

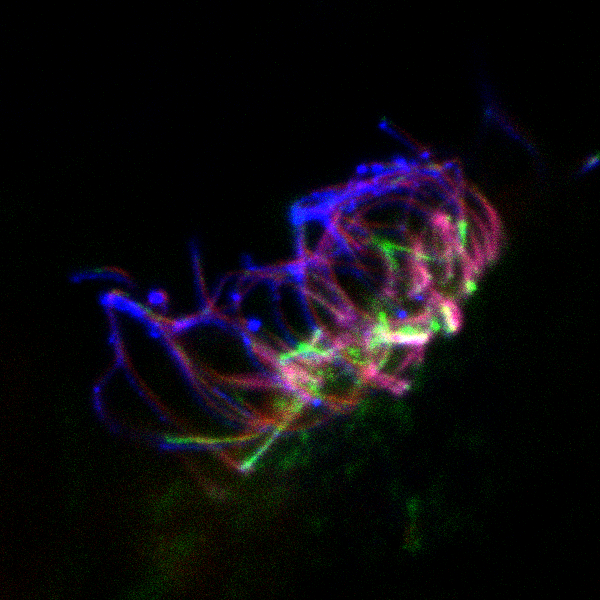

Supplement: Supplementary file 14 — Source data Fig. 6 [file 44319_2025_671_MOESM14_ESM.zip › SD figure 6/6A WT WDR47 confocal.tif]

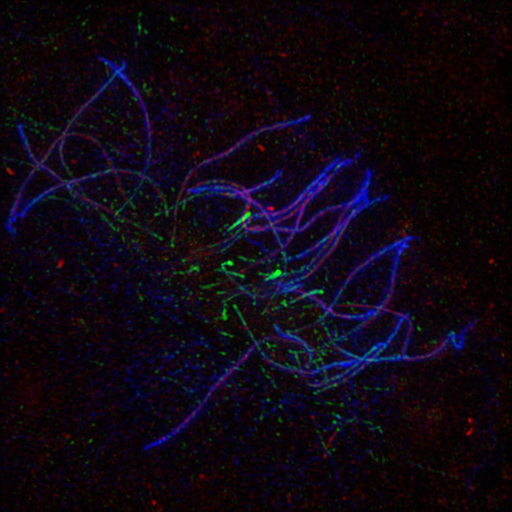

Supplement: Supplementary file 14 — Source data Fig. 6 [file 44319_2025_671_MOESM14_ESM.zip › SD figure 6/6C WT WDR47 SIM.tif]

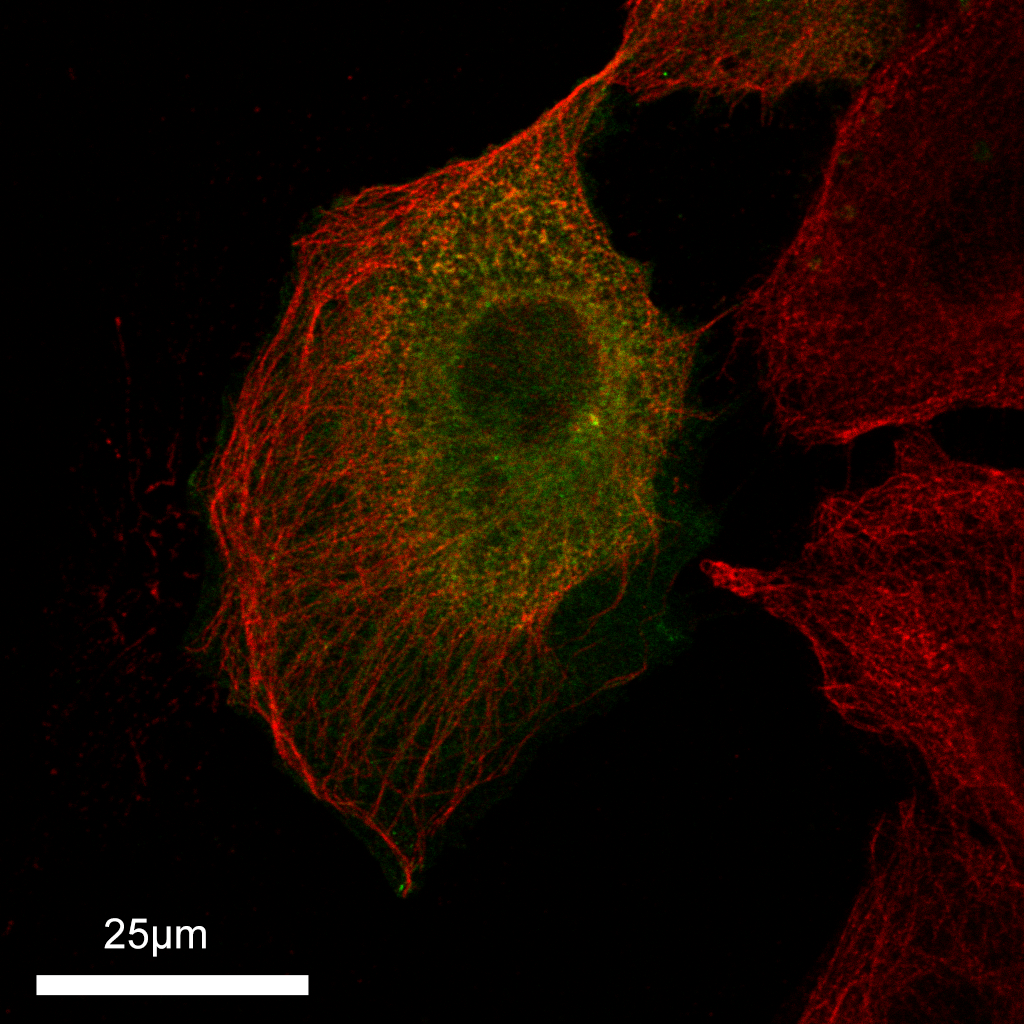

Supplement: Supplementary file 14 — Source data Fig. 6 [file 44319_2025_671_MOESM14_ESM.zip › SD figure 6/6E GFP-WDR47 aTub.tif]

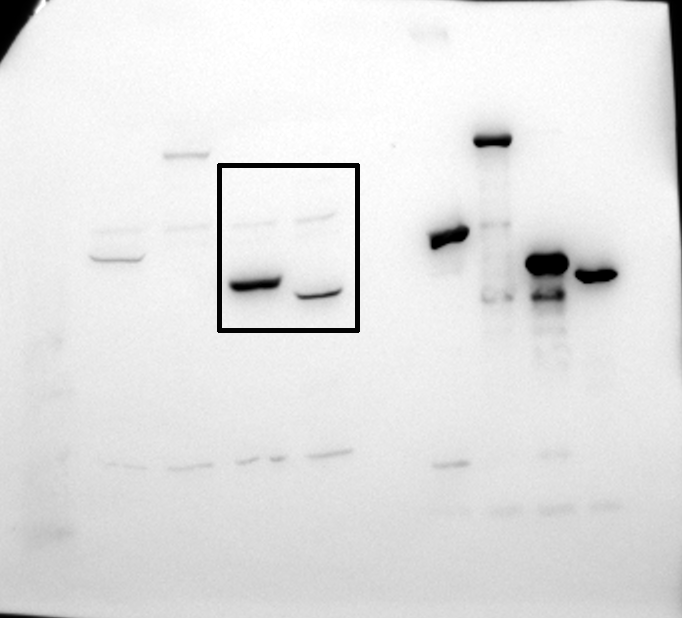

Supplement: Supplementary file 14 — Source data Fig. 6 [file 44319_2025_671_MOESM14_ESM.zip › SD figure 6/6I FLAG input.tif]

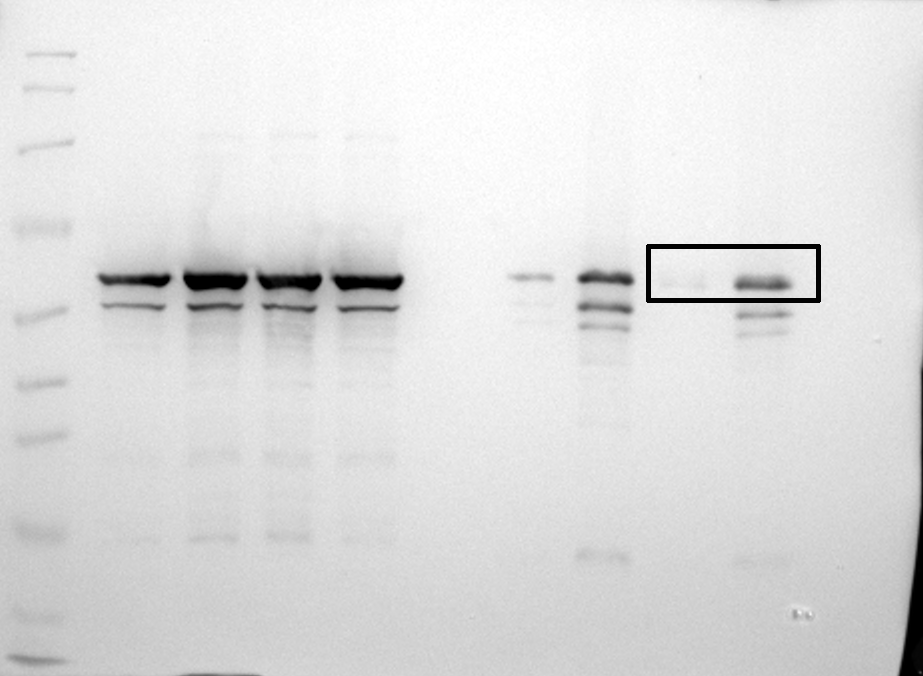

Supplement: Supplementary file 14 — Source data Fig. 6 [file 44319_2025_671_MOESM14_ESM.zip › SD figure 6/6I GFP IP.tif]

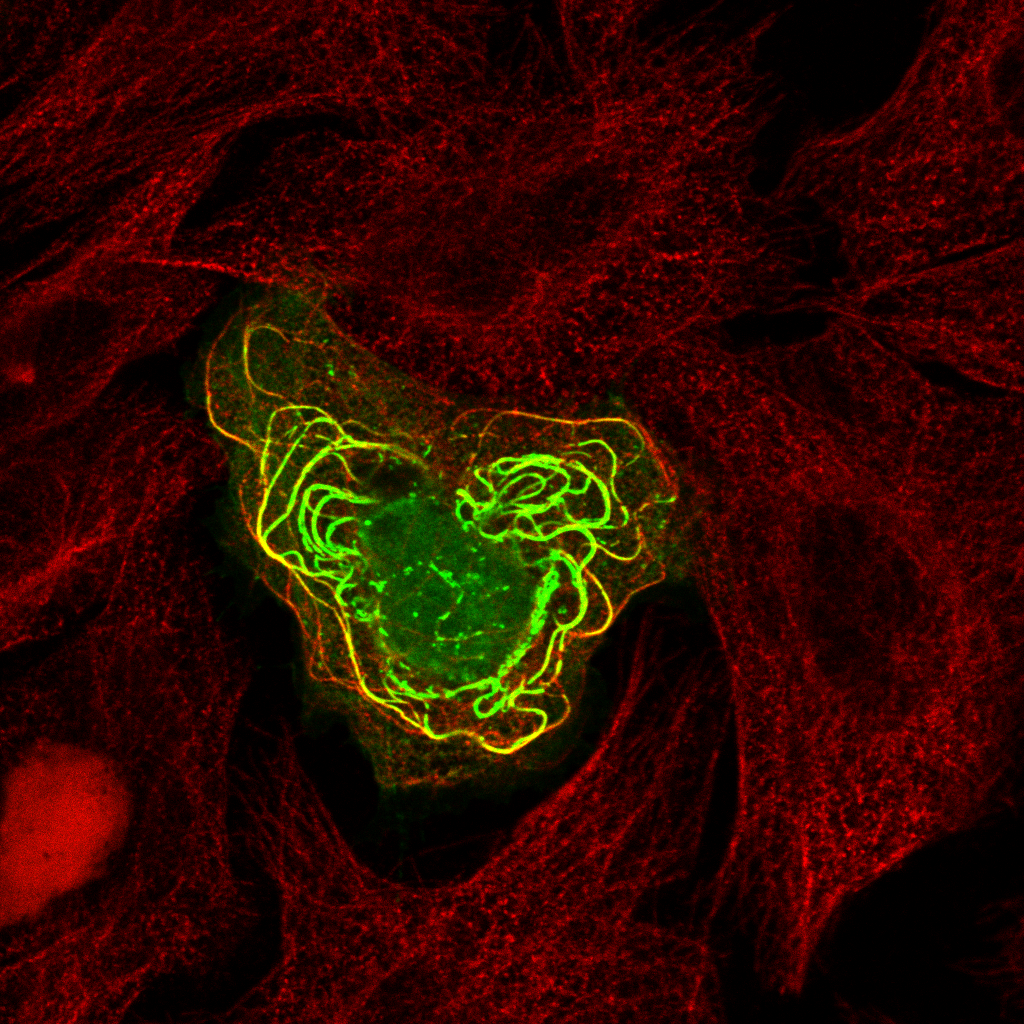

Supplement: Supplementary file 14 — Source data Fig. 6 [file 44319_2025_671_MOESM14_ESM.zip › SD figure 6/6E GFP-SPEF1 aTub.tif]

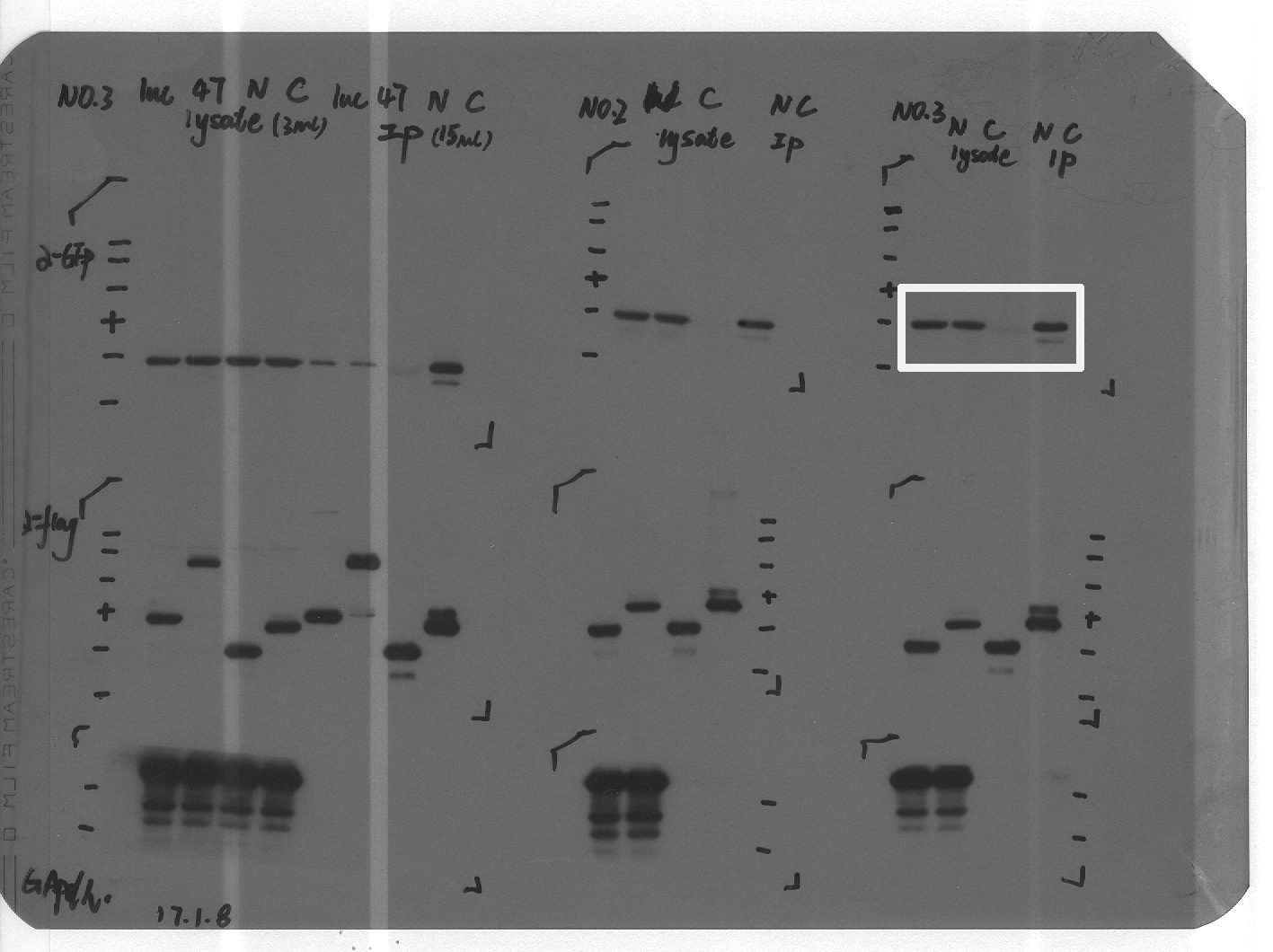

Supplement: Supplementary file 14 — Source data Fig. 6 [file 44319_2025_671_MOESM14_ESM.zip › SD figure 6/6G GFP.tif]

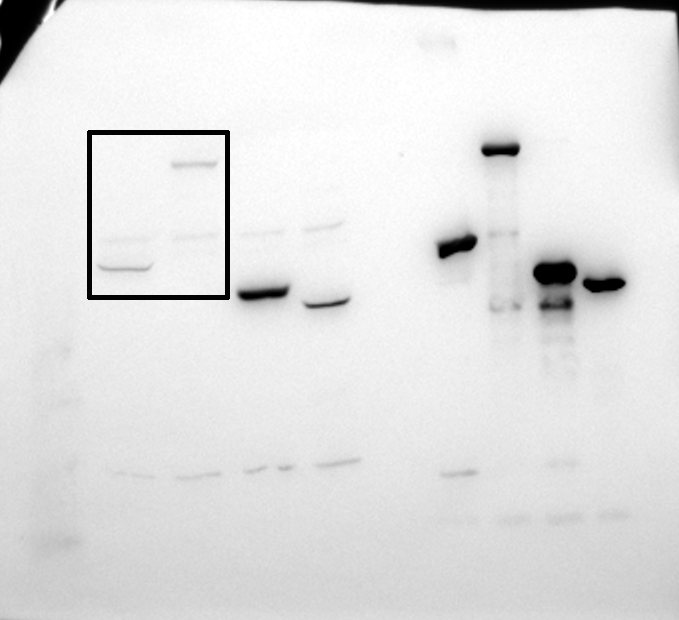

Supplement: Supplementary file 14 — Source data Fig. 6 [file 44319_2025_671_MOESM14_ESM.zip › SD figure 6/6H FLAG input.tif]

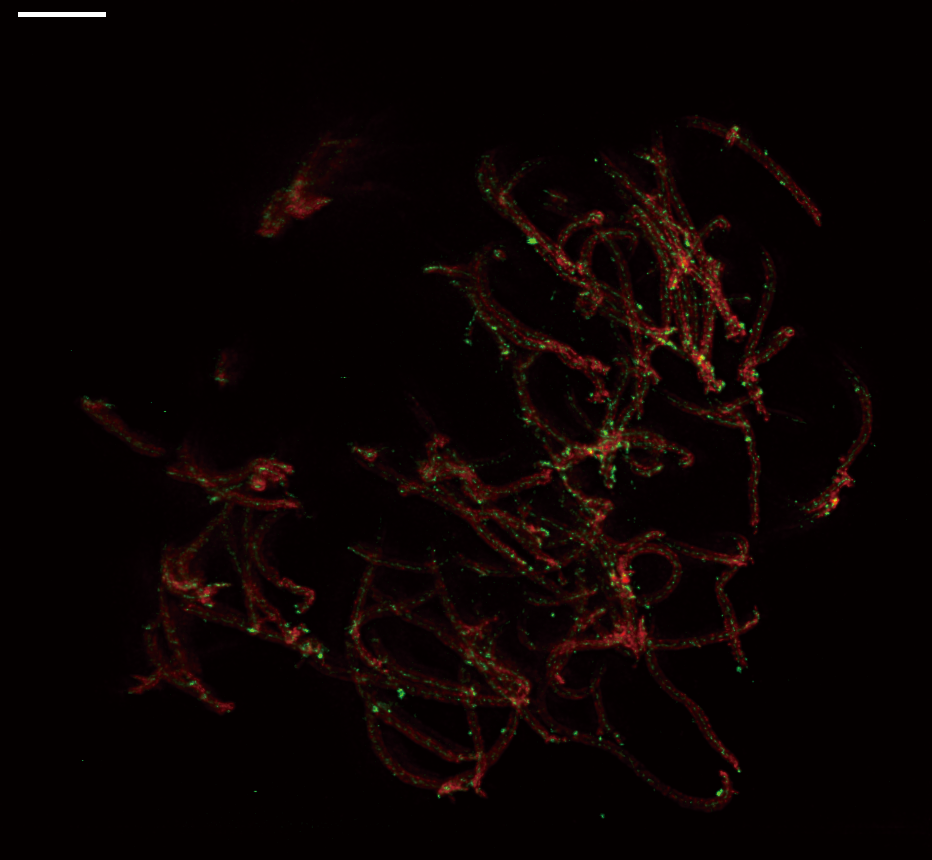

Supplement: Supplementary file 15 — Figure EV1 Source Data [file 44319_2025_671_MOESM15_ESM.zip › SD EV/EV1B SPEF1.tif]

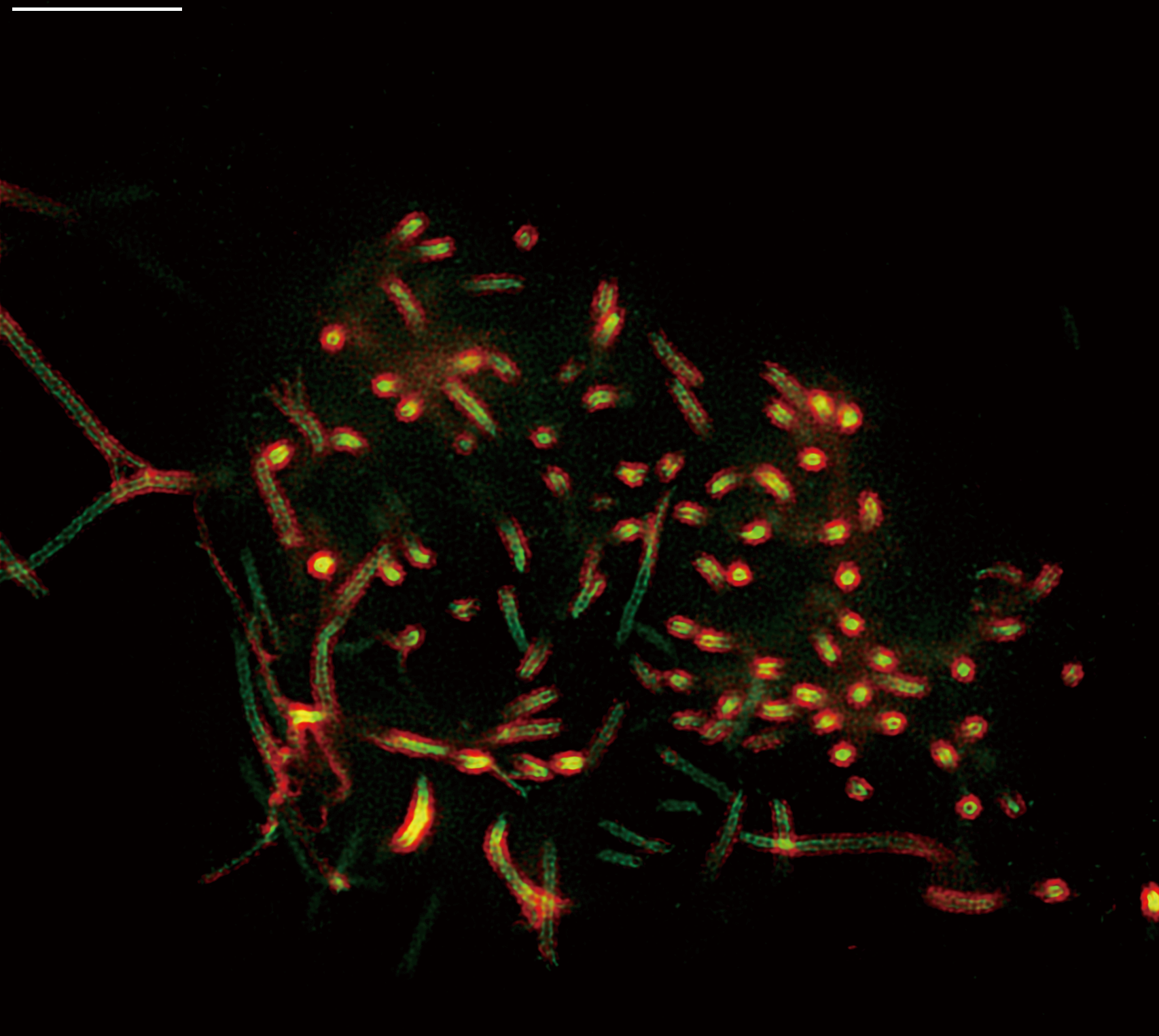

Supplement: Supplementary file 15 — Figure EV1 Source Data [file 44319_2025_671_MOESM15_ESM.zip › SD EV/EV1A RSPH4.tif]
