# Supplementary material for: JHY enables the transition from switchable to fixed ciliary waveforms in metazoan evolution
Source: EMBO Rep. 2025 Dec 10;27(5):1161–79. doi: 10.1038/s44319-025-00671-7 (PMC12979858; doi:10.1038/s44319-025-00671-7)
Supplement: Supplementary file 16 — Expanded View Figures [file 44319_2025_671_MOESM16_ESM.pdf]

Expanded View Figures

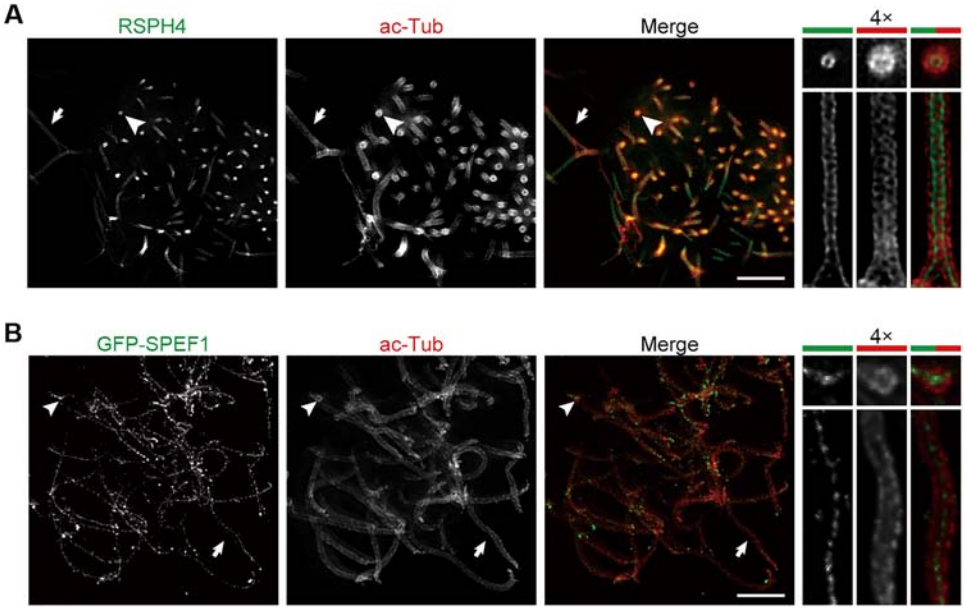

Figure EV1. Discovery of new central lumen proteins with super-resolution microscopy.

(**A**) STED images of mEPCs immunostained with the indicated antibodies. Cells were serum-starved to induce multiciliogenesis and labeled with antibodies against acetylated α-tubulin (ac-Tub) and RSPH4. Magnified images on the right show the longitudinal and transverse views, indicated by arrows and arrowheads, respectively. Note that RSPH4 displays a ring-like distribution in the central lumen. Scale bar, 2 μm. (**B**) STED images of mEPCs exogenously expressing GFP-SPEF1. Cells were serum-starved to induce multiciliogenesis and labeled with an acetylated α-tubulin (ac-Tub) antibody. Magnified images on the right show the longitudinal and transverse views indicated by arrows and arrowheads. Scale bar, 2 μm. Source data are available online for this figure.

© The Author(s)
